# Modeling the Distribution of Diprotic Basic Drugs in Liposomal Systems: Perspectives on Malaria Nanotherapy

**DOI:** 10.3389/fphar.2019.01064

**Published:** 2019-09-25

**Authors:** Ernest Moles, Maria Kavallaris, Xavier Fernàndez-Busquets

**Affiliations:** ^1^Children’s Cancer Institute, Lowy Cancer Research Centre, UNSW Sydney, Randwick, NSW, Australia; ^2^School of Women’s and Children’s Health, UNSW Sydney, Sydney, NSW, Australia; ^3^ARC Centre of Excellence in Convergent Bio-Nano Science and Technology, Australian Centre for NanoMedicine, UNSW Sydney, Sydney, NSW, Australia; ^4^Nanomalaria Group, Institute for Bioengineering of Catalonia (IBEC), The Barcelona Institute of Science and Technology, Barcelona, Spain; ^5^Barcelona Institute for Global Health (ISGlobal, Hospital Clínic-Universitat de Barcelona), Barcelona, Spain; ^6^Nanoscience and Nanotechnology Institute (IN2UB), University of Barcelona, Barcelona, Spain

**Keywords:** partition coefficient, distribution coefficient, polyprotic drug, pH-controlled drug encapsulation, targeted drug delivery, liposomal systems, malaria therapy, nanomedicine

## Abstract

Understanding how polyprotic compounds distribute within liposome (LP) suspensions is of major importance to design effective drug delivery strategies. Advances in this research field led to the definition of LP-based active drug encapsulation methods driven by transmembrane pH gradients with evidenced efficacy in the management of cancer and infectious diseases. An accurate modeling of membrane-solution drug partitioning is also fundamental when designing drug delivery systems for poorly endocytic cells, such as red blood cells (RBCs), in which the delivered payloads rely mostly on the passive diffusion of drug molecules across the cell membrane. Several experimental models have been proposed so far to predict the partitioning of polyprotic basic/acid drugs in artificial membranes. Nevertheless, the definition of a model in which the membrane-solution partitioning of each individual drug microspecies is studied relative to each other is still a topic of ongoing research. We present here a novel experimental approach based on mathematical modeling of drug encapsulation efficiency (EE) data in liposomal systems by which microspecies-specific partition coefficients are reported as a function of pH and phospholipid compositions replicating the RBC membrane in a simple and highly translatable manner. This approach has been applied to the study of several diprotic basic antimalarials of major clinical importance (quinine, primaquine, tafenoquine, quinacrine, and chloroquine) describing their respective microspecies distribution in phosphatidylcholine-LP suspensions. Estimated EE data according to the model described here closely fitted experimental values with no significant differences obtained in 75% of all pH/lipid composition-dependent conditions assayed. Additional applications studied include modeling drug EE in LPs in response to transmembrane pH gradients and lipid bilayer asymmetric charge, conditions of potential interest reflected in our previously reported RBC-targeted antimalarial nanotherapeutics.

## Introduction

A wide range of therapeutic nanoparticles in the form of nanocarrier-based delivery systems have been developed so far for the management of several medical conditions aiming to improve treatment outcomes while minimizing drug dosages (Anselmo and Mitragotri, 2016; Bobo et al., 2016). Among these, liposomes (LPs) have proven to be notably effective in the treatment of cancer, fungal infections, and age-related disorders, among other therapeutic purposes, as well as in analgesia and vaccine formulations (Allen and Cullis, 2013; Bulbake et al., 2017). Given their biphasic character analogous to biological membranes, LPs are capable of entrapping both lipophilic and hydrophilic compounds as well as buffer solutions (Torchilin, 2005; Pattni et al., 2015; Sercombe et al., 2015). Such particular features have been exploited for the generation of transmembrane pH and chemical gradients, which in turn drive the encapsulation of water-soluble, amphiphilic polyprotic drugs as a result of pH-driven variations in drug microspecies abundance (Madden et al., 1990; Cullis et al., 1991) and/or following the formation of drug precipitates in complexation with multivalent salts (Haran et al., 1993; Clerc and Barenholz, 1995; Wei et al., 2018).

**Figure 1:**
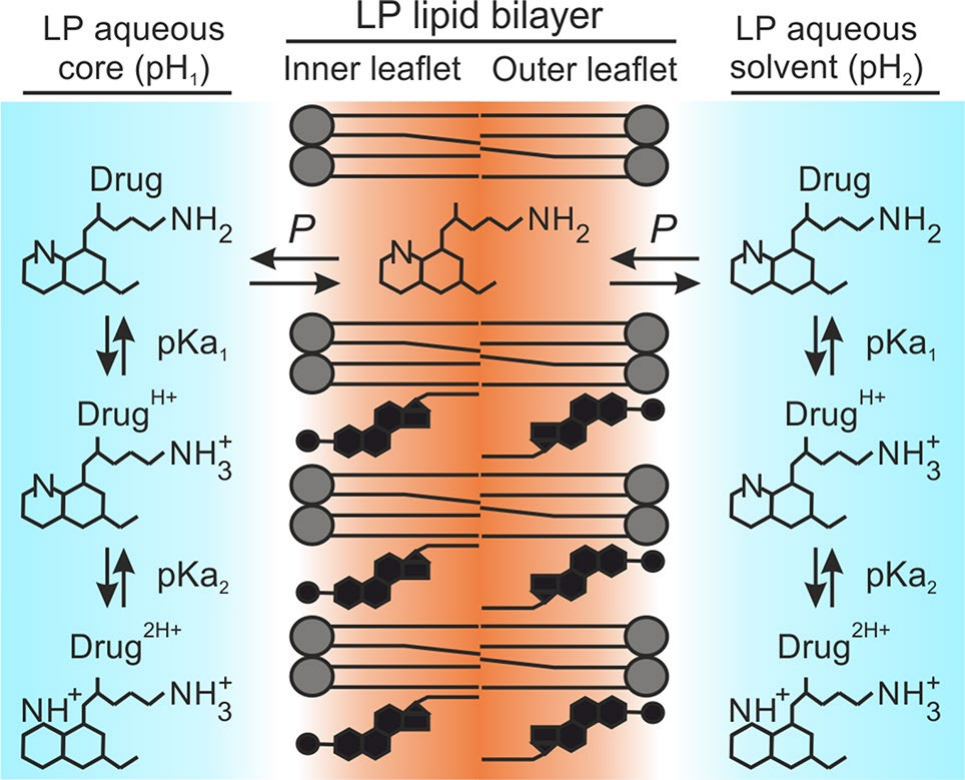
*D*_P_ distribution model. Illustration adapted for amphiphilic diprotic basic drugs in liposomal systems. Primaquine is used as drug example.

Aforesaid encapsulation strategies rely on the selective partitioning and passive diffusion of unionized drug molecules, i.e., unionized microspecies, across the LP membrane. Such migratory process is triggered in response to drug concentration gradients between LP compartments (Gubernator, 2011; Sercombe et al., 2015) and persists until an equilibrium concentration is reached between the LP membrane (organic) and solvent (aqueous) fractions. Such ratio is indicative of drug lipophilicity and is generally expressed in the literature in the form of partition or distribution coefficients (*P* and *D*), depending on the respective absence or presence of ionized drug molecules, i.e., ionized microspecies, in solution (Leo et al., 1971; Scherrer and Howard, 1977; Hansch et al., 1995; Warhurst et al., 2003; Warhurst et al., 2007). An example of *P* and *D* calculation for a diprotic basic drug is illustrated in Equations 1–5. Furthermore, coefficient *D* is utilized to calculate LP encapsulated amounts for polyprotic drugs in the presence of predefined transmembrane pH gradients (Cullis et al., 1991), as exemplified in Equation 6. The resulting distribution model is referred here as *D*_P_ given the initial assumption of unionized drug molecules as sole microspecies able to diffuse into the lipid bilayer (Hansch et al., 1995; Warhurst et al., 2003; Tetko and Poda, 2004; Warhurst et al., 2007; Omodeo-Salè et al., 2009). This assumption has been theorized in a large number of works and is represented in **Figure 1** as an adaptation for liposomal systems.

(1) $$P=\frac{{\mathrm{mol Drug}}_{\mathrm{org}}}{{\mathrm{Vol}}_{\mathrm{org}}}/\frac{{\mathrm{mol Drug}}_{\mathrm{aq}}}{{\mathrm{Vol}}_{\mathrm{aq}}}$$

(2) $$D=\frac{{\mathrm{mol Drug}}_{\mathrm{org}}}{{\mathrm{Vol}}_{\mathrm{org}}}/\frac{{\mathrm{mol Drug}}_{\mathrm{aq}}+{\mathrm{mol Drug}}_{\mathrm{aq}}^{H+}+{\mathrm{mol Drug}}_{\mathrm{aq}}^{2H+}}{{\mathrm{Vol}}_{\mathrm{aq}}}$$

(3) $$D=\left(\frac{{\mathrm{mol Drug}}_{\mathrm{org}}\times {\mathrm{Vol}}_{\mathrm{aq}}}{{\mathrm{Vol}}_{\mathrm{org}}\times {\mathrm{mol Drug}}_{\mathrm{aq}}}\right)/\left(1+\frac{{\mathrm{mol Drug}}_{\mathrm{aq}}^{H+}}{{\mathrm{mol Drug}}_{\mathrm{aq}}}+\frac{{\mathrm{mol Drug}}_{\mathrm{aq}}^{2H+}}{{\mathrm{mol Drug}}_{\mathrm{aq}}}\right)$$

(4) $$\begin{array}{l} \mathrm{pH}={\mathrm{pKa}}_{1}+\log\left(\frac{\mathrm{mol Drug}}{{\mathrm{mol Drug}}^{H+}}\right);\\ \mathrm{pH}={\mathrm{pKa}}_{2}+\log\left(\frac{{\mathrm{mol Drug}}^{H+}}{{\mathrm{mol Drug}}^{2H+}}\right) \end{array}$$

(5) $$\log D=\log P-\log(1+10^{\left({\mathrm{pKa}}_{1}-\mathrm{pH}\right)}+10^{\left({\mathrm{pKa}}_{1}+{\mathrm{pKa}}_{2}-2\times \mathrm{pH}\right)})$$

(6) [Drugaq+DrugaqH++Drugaq2H+]in[Drugaq+DrugaqH++Drugaq2H+]out=1+10(pKa1−pHin)+10(pKa1+pKa2−2×pHin)1+10(pKa1−pHout)+10(pKa1+pKa2−2×pHout)

However, amphiphilic polyprotic drugs, which indeed account for most of the therapeutic agents used in the clinic (Charifson and Walters, 2014), exhibit complex structural and physicochemical properties with multiple ionization states being present in physiological conditions, each one displaying a different degree of lipophilicity depending on (i) the overall number of ionized groups, as well as (ii) their position within the drug structure. In particular, some degree of interaction with biological membranes would be expected for drugs with size and 3D configuration similar to phospholipid molecules and, above all, those microspecies with the lowest ionization state and/or in which ionized moieties are excluded from the most lipophilic regions. Some examples of drugs fulfilling these requisites include quinoline derivatives, such as the 4-/8-aminoquinoline and naphtoquinoline compounds, aminoalcohols, acridine derivatives, and anthracyclines, among many other drugs utilized for malaria and cancer therapy (Vennerstrom et al., 1999; Kaschula et al., 2002; Bawa et al., 2010; Shaul et al., 2013; Soares et al., 2015).

Variations in the interaction of polyprotic drugs with lipid bilayers have been reported as a function of pH and phospholipid charge, feature reflected by experimental changes in coefficient *D* (Ottiger and Wunderli-Allenspach, 1997; Krämer et al., 1998; Xia et al., 2005; Nair et al., 2012). Nevertheless, the separate contribution of each single microspecies to define the overall distribution of drugs in liposomal systems has never been studied. A detailed understanding of the partitioning behavior of all microspecies present in the system is essential to accurately model drug interactions with biological and synthetic membranes, and for the development of more effective drug delivery strategies.

Based on the above considerations and our experimentally collected data, we present here novel distribution models that accurately predict the distribution in LP suspensions of various diprotic basic antimalarials of major clinical significance. Using experimental encapsulation data in phosphatidylcholine-LPs, and the knowledge of *P* and pKa values for antimalarial drugs, microspecies-specific partition coefficients were estimated as a function of pH and phospholipid compositions that simulate the membrane properties of the red blood cell (RBC), the host cell for *Plasmodium falciparum* blood stages. With the aim to improve the design of our previously developed RBC-targeted LP models for severe malaria therapy (Moles et al., 2015; Moles et al., 2017), the distribution models described here were applied to estimate antimalarial drug encapsulation and release in response to transmembrane pH gradients along with their distribution within RBC compartments.

## Materials and Methods

### Reagents and Chemicals

Except where otherwise indicated, reagents were purchased from Merck and Co., Inc. (Kenilworth, NJ, USA), and reactions were performed at room temperature (22 to 24°C). Anhydrous quinine (QN, ≥98% purity, 173–175°C mp), primaquine diphosphate salt (PQ, ≥98% purity, 205–206°C mp), tafenoquine succinate (TQ, ≥95% purity, 146–149°C mp), quinacrine dihydrochloride (QC, ≥90% purity, 248–250°C mp), and chloroquine diphosphate salt (CQ, ≥98% purity, 200°C mp) were purchased in solid form and used without further purification. The lipids 1,2-dioleoyl-*sn*-glycero-3-phosphocholine (PC), 1,2-dioleoyl-*sn*-glycero-3-phosphoethanolamine (PE), 1,2-distearoyl-sn-glycero-3-phosphoethanolamine-N-[methoxy(polyethylene glycol)-2000] (PE-PEG), and 1,2-dioleoyl-*sn*-glycero-3-phosphoethanolamine-N-[lissamine rhodamine B sulfonyl] (PE-Rho, used for LP tracking purposes) were purchased as solid material from Avanti Polar Lipids, Inc. (Alabaster, AL, USA). Phosphatidylserine (PS, average relative molar mass of 788) was obtained from bovine spinal cord, and was supplied in solid form by Lipid Products, Ltd. (South Nutfield, Redhill, UK). Purity for purchased lipids (≥95%), cholesterol (≥99%, 360°C bp), and drugs is reported by the respective suppliers according to HPLC analysis.

### LP Phospholipid Compositions, LP Preparation, and Drug Partitioning Analysis

Phospholipid compositions (mole ratios) for the LP suspensions assayed in this work were: 1) PC-LPs (cholesterol:PE-Rho:PC, 20:0.5:77.5); 2) PC:PS-LPs (cholesterol:PE-Rho:PC:PS, 20:0.5:53.1:26.4); 3) PC:PS:PE-LPs (cholesterol:PE-Rho:PC:PS:PE, 40:0.5:13.9:19.8:25.8); and 4) PC:PS:PE:PEG-LPs (cholesterol:PE-Rho:PC:PS:PE:PE-PEG, 40:0.5:13.9:19.8:20.8:5).

LP suspensions were prepared by the lipid film hydration method in combination with particle extrusion through polycarbonate membranes (MacDonald et al., 1991). Briefly, stock lipids in chloroform were mixed and dissolved in chloroform:methanol (2:1 v/v) in a round-bottom flask, and the organic solvents were subsequently removed by rotary evaporation under reduced pressure at 37°C. The resulting dry lipid film was then hydrated in phosphate-buffered saline (PBS) (pH 7.4), or alternatively in citrate-/phosphate-/tris-buffered saline solutions at pH 4.0/6.5/9.0 when studying drug partitioning in non-physiological pH conditions. PBS was used as solvating buffer for drug partitioning analysis in the presence of negatively charged, PS-containing LPs. Unilamellar vesicles at 10 mM lipid, ca. 135 to 185 nm in diameter, were obtained upon lipid film hydration by four cycles of constant vortexing coupled to bath sonication (3 min each), followed by extrusion through 200-nm polycarbonate membranes in an extruder device (Avanti Polar Lipids, Inc.). Throughout the lipid film hydration and downsizing processes, samples were maintained above the lipids’ transition temperature. Sterility of LP suspensions was preserved by rinsing all material in 70% ethanol and working in a laminar flow hood. For the characterization of LP surface charge and size by ζ-potential determination and dynamic light scattering, samples were diluted 1:30 in deionized water (Milli-Q^®^ system; Millipore) and PBS, respectively, and analyzed in a Zetasizer NanoZS90 instrument (Malvern Ltd, Malvern, UK). Electrolyte concentration in diluted samples was sufficient for ζ-potential measurement.

When studying antimalarial partitioning in LP suspensions, 5 mM drug stocks were initially prepared in water and subsequently mixed with LPs at a 1:40 drug to lipid mole ratio (0.25 mM drug for 10 mM lipid), followed by 24-h incubation under orbital stirring. According to previous works, this incubation time led drugs to reach partition equilibrium upon their passive entrapment in LP organic and aqueous fractions, i.e., LP lipid bilayer plus aqueous core (Moles et al., 2015). As expected, given the drug:lipid ratio used, particle size and ζ-potential remained minimally affected after the addition of drugs with <10% percentual differences obtained (**Table S1**). Similar variations were also obtained at the different pH values studied here (data not shown).

To quantify entrapped drug amounts in LPs, these were pelleted by ultracentrifugation (150,000*g*, 4°C, 1 h) and treated with 1% sodium dodecyl sulphate coupled to 60°C bath sonication as previously reported (Moles et al., 2017). Drug extracts were analyzed by UV-visible spectroscopy using an Epoch^TM^ spectrophotometer (BioTek Instruments, Inc., Winooski, VT, USA) in 96-well plate mode. Drug standards for quantification were prepared in 1% sodium dodecyl sulphate, and the same solvent was used as blank control for absorbance subtraction. Standard curves were obtained by linear regression from at least three independent measurements (**Figure S1**). Unencapsulated drug amounts were determined by UV-visible spectroscopy from LP supernatants. Drug encapsulation efficiency (EE) was finally determined as the percentual amount of drug retained in LPs relative to the total amount present in the sample (LPs + external solution).

### Antimalarial Drug Theoretical Distribution Modeling

The modeling of antimalarial drug distribution in liposomal systems was performed through the design of a sequential experimental method that comprises the following steps: i) an initial construction of a vesicular-like multicompartment system with aqueous/organic volumetric fractions corresponding to the experimental conditions used (e.g., lipid concentration, LP size, pH); ii) the subsequent theorization of a distribution model (*D* *_P_*, *D* *_P_*_,_ *_PH+_*, and *D* *_P_*_,_ *_PH+_*_,_ *_P2H+_* defined here) which will delineate the abundance of drug microspecies present in aqueous solution along with their partitioning behavior, dependent on drug pKa and microspecies-specific partition coefficients (e.g., *P*, *P* *^H+^*, and *P* *^2H+^* for diprotic basic drugs); iii) estimation of hypothetical *P* *^H+^*, *P* *^2H+^* values (log_10_ units) and computing of theoretical EE values for the predefined vesicular system and distribution model; iv) isolation of *P* *^H+^*, *P* *^2H+^* values with least experimental versus theoretical EE variance; v) a final analysis of the goodness of the distribution model considered along with fitted *P* *^H+^*, *P* *^2H+^* values to describe experimental data. All calculations and graphical representations were performed using an algorithm created for Wolfram Mathematica 8.0 computing software (The Wolfram Centre, Oxford, UK). The complete set of algorithms used in this work are reported in **Supplementary Material**.

#### Construction of LP- and RBC-Like Vesicular Systems and Antimalarial Drug Distribution Modeling

For the pH- and lipid charge-dependent modeling of antimalarial drug distribution in our liposomal systems (LPs at 10 mM lipid in aqueous solution), we constructed a vesicular-like multicompartment system using the following parameters reported elsewhere (Lewis and Engelman, 1983; Petrache et al., 2000; Rawicz et al., 2000; Maurer et al., 2001; Leitmannova Liu, 2006): i) vesicles of 100 nm diameter; ii) lipid bilayer thickness of 5 nm, which includes phospholipid fatty acid chains (ca. 2.6–3.0 nm), glycerol (0.3 nm), and phosphocholine head group (0.7–1 nm); iii) vesicle internal aqueous versus membrane volume ratio of 2.7; iv) vesicle internal aqueous volume relative to total lipid molecules in the system of 2.4 µl solution/µmol lipid; and v) lipid molecule ratio between outer and inner lipid bilayer leaflets of 54/46. Resulting volumetric ratios for all organic/aqueous fractions present in the system at 10-mM lipid concentration are as follows: 2.40 (total LP aqueous cargo), 0.48 and 0.41 (outer and inner LP lipid bilayer leaflets), and 96.71 (solution external to LPs).

For the modeling of antimalarial distribution in RBC suspensions, a second vesicular multicompartment system was constructed replicating human RBC dimensions, physiological hematocrit and phospholipid charge asymmetry, i.e., i) total volume of the system occupied by vesicles of 40%; ii) vesicle volume and surface area of, respectively, 90 fl and 140 µm^2^ (McLaren et al., 1987; Schrier, 2012; Parisio et al., 2013); iii) lipid bilayer thickness of 5 nm (Maurer et al., 2001; Leitmannova Liu, 2006), which makes an organic volume of 0.45 fl for each single vesicle; and iv) phosphate buffer at pH 7.4 as extravesicular solution. Resulting volumetric ratios for all organic/aqueous fractions present in the system at 40% hematocrit are as follows: 39.8 (total RBC aqueous cargo), 0.1/0.1 (outer and inner RBC plasma membrane leaflets), and 60.0 (extracellular solution).

Antimalarial distribution in aforesaid LP- and RBC-like vesicular systems was subsequently computed through the following sequential steps: i) determination of the molecular abundance of major drug microspecies present in aqueous fractions for the experimental pH range studied (pH 4.0–9.0), providing previous knowledge of drug pKas (Equation 4); ii) drug microspecies partitioning between aqueous solution and organic (LP and RBC lipid bilayer leaflets) fractions was thereafter determined as a function of the distribution model considered (*D* *_P_*, *D* *_P_*_,_ *_PH+_*, or *D* *_P_*_,_ *_PH+_*_,_ *_P2H+_*) and microspecies-respective partition coefficients (*P*, *P* *^H+^* and *P* *^2H+^*). The percentual amount of drug molecules retained within LP and RBC fractions (lipid bilayer leaflets and aqueous cargo) relative to their total amount present in the system (LP and RBC fractions plus extravesicular solution) was finally expressed as theoretical EE (EEt). More information about the volumetric fractions considered in our LP- and RBC-like vesicular systems, along with examples of drug microspecies distribution and resulting EEt in the abovementioned systems, can be found in **Supplementary Material** (**Figures S2**–**S4** and **Tables S3**–**S6**).

### Statistical Methods

All reported experimental data are defined as mean ± standard deviation from at least three independent sample replicates. Significant differences (*p* values <0.05) in drug theoretical versus experimental EE were determined by *Z*-test estimation comparing theoretical EE values (single value for each condition studied) with the corresponding mean ± standard deviation of experimentally retrieved EE (population mean). The significance of variations among drug experimental EE for the conditions studied here, comparing mean ± standard deviation of pH- and lipid composition-dependent retention yields, was analyzed by *t*-test score; differences were considered significant for *p* values <0.05.

## Results and Discussion

### Rationale Behind LP Formulation and Drug Partitioning Analysis

The initial aim of this work was to study the effect of differences in pH and RBC membrane-analogous phospholipid compositions over the distribution of polyprotic antimalarials in LP suspensions. To do so and following previous works (Moles et al., 2015; Moles et al., 2016), a neutrally charged LP formulation based on phosphatidylcholine (PC):cholesterol at mole ratios 80:20 (PC-LPs), pH 7.4, was selected as standard physiological condition. Choline-containing phospholipids, mainly PC and phosphatidylethanolamine (PE), account for half of all lipids found in mammalian cell membranes, followed by 30% to 40% cholesterol (Chabanel et al., 1983; Virtanen et al., 1998; Arbustini, 2007; Leventis and Grinstein, 2010). Variations in solution pH and phospholipid composition were applied during LP preparation by, respectively, i) PC-LP lipid film hydration in different pH buffer solutions, and ii) the replacement of stock phospholipids in organic lipid mixtures followed by their subsequent hydration at pH 7.4 buffer.

Considering our ultimate goal to advance in the design of RBC-targeted, LP-based nanotherapeutics with improved prophylactic activity against *P. falciparum* intraerythrocytic stages, an alternative lipid composition consisting of 40% cholesterol and 60% phospholipid, mainly PC:phosphatidylserine (PS):PE at mole ratios 23:33:44 (PC:PS:PE-LPs), was studied reproducing the RBC inner membrane leaflet (Maguire et al., 1991; Virtanen et al., 1998; Ingólfsson et al., 2014). Similar to other works using RBCs as vascular supercarriers (Muzykantov, 2010; Villa et al., 2016), this strategy relies on the selective intracellular loading of therapeutic agents into non-infected RBCs, inhibiting parasite growth upon cell invasion (Moles and Fernàndez-Busquets, 2015; Moles et al., 2015; Moles et al., 2017).

Two additional LP formulations were considered to assess i) the influence on PC-based bilayers of PS (PC:PS-LPs) as major anionic phospholipid responsible for the asymmetric charge properties found in mammalian cell membranes (Fadeel and Xue, 2009; Marquardt et al., 2015), and ii) the effect of LP surface steric stabilization in RBC-like lipid bilayers by including 5% PEG-derivatized PE (PC:PS:PE:PEG-LPs).

Moreover, drug partitioning in LP suspensions was assessed in terms of Encapsulation Efficiency (EE), the percentual amount of drug retained within the LP fraction (LP internal aqueous core + lipid bilayer) relative to its total amount present in the system (LP fraction + external solvent). To prevent LP membrane saturation by supplemented antimalarials, these were assayed at 1:40 drug:lipid mole ratio, well below the maximum 1:20 reported for lipophilic compounds not disturbing the lipid bilayer structure (Fahr et al., 2005).

### pH-Dependent Partitioning of Antimalarial Drugs in PC-LP Suspensions

**Figure 2:**
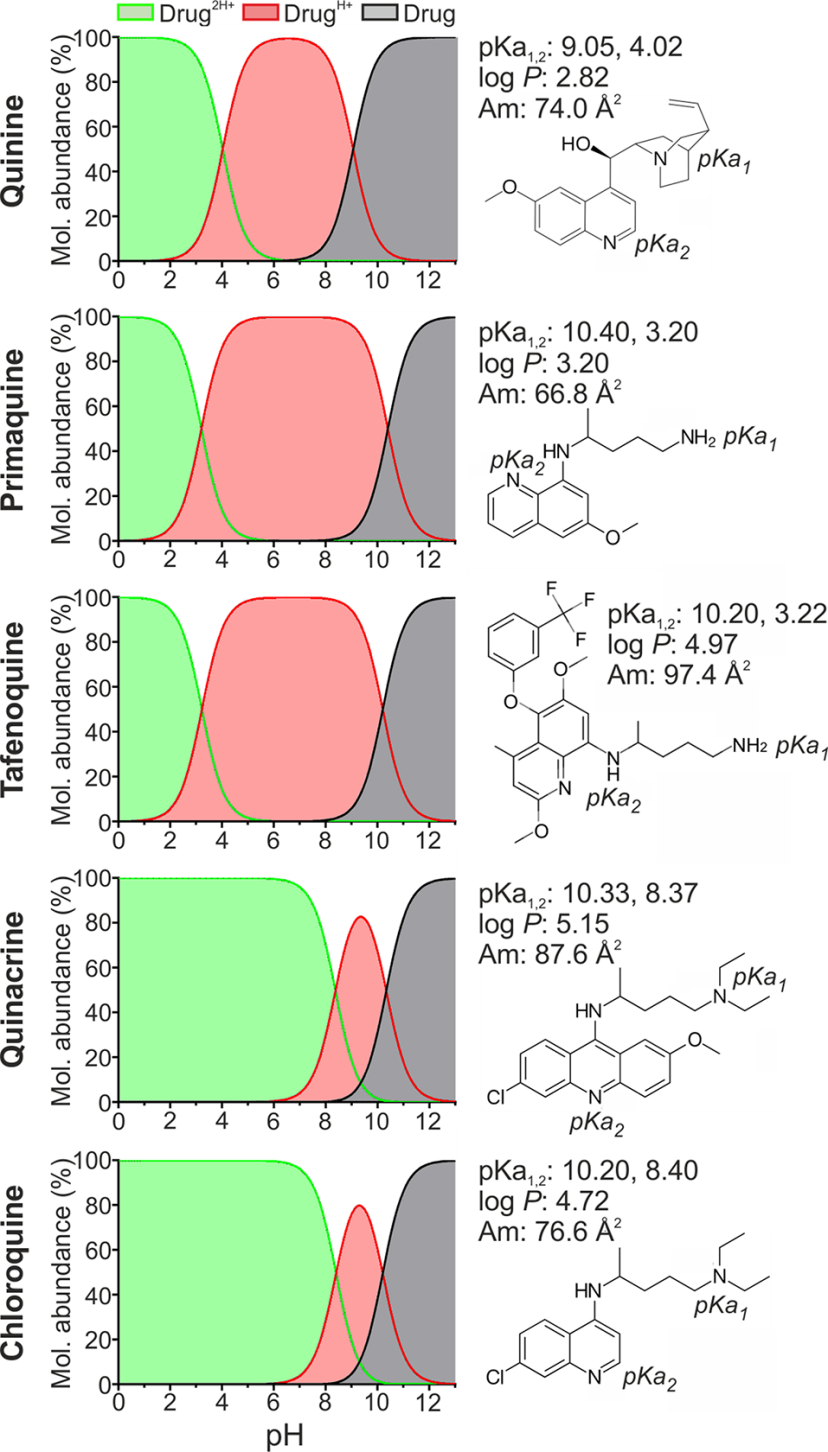
Structure and physicochemical properties of the antimalarial drugs studied here. Molecular abundances in solution at pH 0 to 13 are illustrated for major drug microspecies: unionized (Drug), monoprotonated (Drug^H+^) and diprotonated (Drug^2H+^). pKa, molecular mean projection area (Am), and log *P* values were determined using the Chemicalize software developed by ChemAxon Ltd., except for CQ and PQ, whose pKa and log *P* have been experimentally determined elsewhere (Omodeo-Salè et al., 2009; Nair et al., 2012).

The polyprotic antimalarials studied in this work were all diprotic weak bases and belong to the amino-alcohol (quinine, QN), 8-aminoquinoline (primaquine, PQ, and tafenoquine, TQ), 4-aminoquinoline (chloroquine, CQ), and 9-aminoacridine (quinacrine, QC) drug classes (**Figure 2**). These agents were selected considering: i) their clinical and translational significance (World Health Organization, 2012; World Health Organization, 2015); ii) applicability for LP-based nanotherapeutics relying on pH gradient and ammonium sulfate active encapsulation systems (Haran et al., 1993; Moles et al., 2017); iii) the presence of at least two ionizable groups at a pH range of 4.0 to 9.0, selected to avoid causing harmful effects on phospholipid stability while maintaining LP charge (Tsui et al., 1986; Moncelli et al., 1994), and iv) their lipophilic nature, reflected by drug *P* values >2 log_10_ units. All the antimalarials tested here exhibit sizes similar to phospholipid molecules, which can reach approximately 60 to 70 Å^2^ average area for choline phospholipids (Petrache et al., 2000). This feature was prioritized to facilitate stable drug diffusion across lipid bilayers.

**Figure 3:**
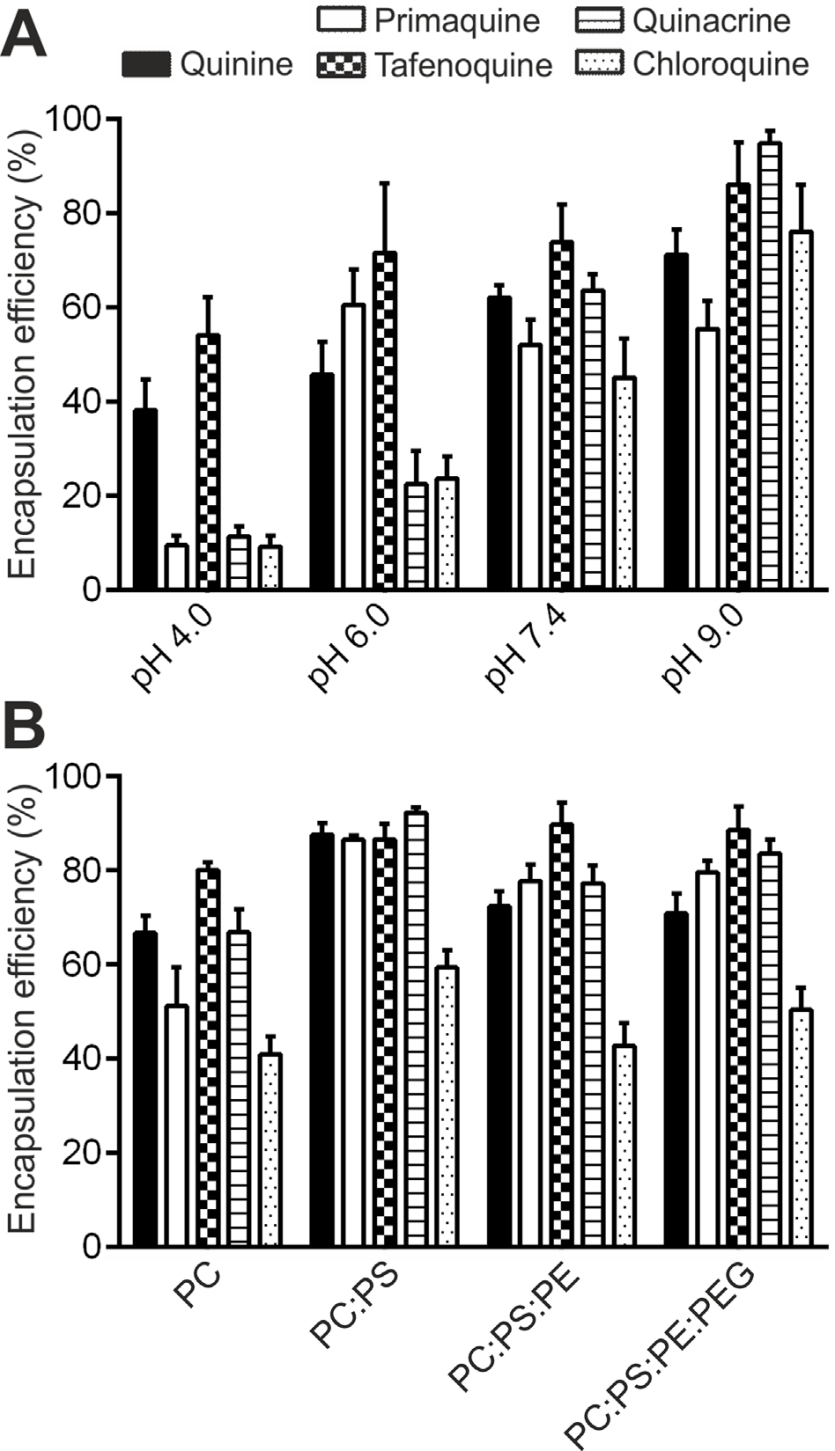
Experimental EEs of antimalarials in LP suspensions. EE was determined as a function of **(A)** solution pH for PC-LP suspensions, and **(B)** phospholipid composition at pH 7.4.

In accordance with antimalarial lipophilicity and basic nature, a positive correlation between experimental EE (EEe) and pH was found for all drugs when incubated for 24 h in PC-LP suspensions (**Figure 3A**). EEe rates exceeding 40% were reached at pH ≥7.4, which highlights the increased capacity of antimalarials to accumulate in LPs in basic conditions. Analogously, lowest EEe values were obtained at the most acidic condition assayed, pH 4.0. Due to the noticeable differences in drug microspecies abundance at pH 4.0 to 9.0 (**Figure 2**), we classified the tested compounds into two main groups: monoprotonated (QN, PQ, TQ; pKa_1,2_ ca. 10, 3.2–4.0) and diprotonated types (CQ, QC; pKa_1,2_ ca. 10.2, 8.4) with the respective presence of Drug^H+^ and Drug^2H+^ as major microspecies for the pH range studied. Significantly larger pH-dependent variations in drug EE were likewise found to be associated with diprotonated-type antimalarials (**Table S2**), such differences reflecting the major role of pKa_2_ 8.4 in defining CQ and QC partitioning at pH ≥4.0.

Moreover, all five antimalarials exhibited EEe values far higher than the theoretical EE (EEt) that would be expected when considering *D* *_P_* as preliminary distribution model, in which the drug unionized form is postulated as the sole microspecies capable of interacting with the lipid bilayer (**Figure 1**). EEt (*D* *_P_*) data are reported in **Table 1** and were calculated as detailed in *Materials and Methods*. Variations in EEe versus EEt (*D* *_P_*) were expressed as percentual difference, or ΔEE (Equation 7). In overall, median |ΔEE|, or $\tilde{∆\mathrm{EE}}$, ranging 55.7% to 85.9% were obtained for all antimalarials considering all four pH conditions studied (**Table 1**). Largest increases in EE were remarkably obtained for monoprotonated-type antimalarials at pH 6.0 (EEe >45% vs. EEt (*D* *_P_*) ≤7.3%), condition in which Drug^H+^ microspecies are dominant with abundances close to 100% (**Figure 2**). This observation evidenced the likely existence of ionized microspecies being stably incorporated in the LP membrane in mild acidic conditions.

(7) $$∆\mathrm{EE}\%=\left(\frac{\mathrm{EEt}-\mathrm{EEe}}{\mathrm{EEe}}\right)\times 100$$

**Table 1:** Experimental vs. theoretical EEs for PC-LP suspensions as a function of pH and *D_P_*, *D_P,PH+_*, *D_P,PH+,P2H+_* drug distribution models.

Drug	pH	%EEe	%EEt (*D* *_P_*)	*ΔEE* (%)/*p* value	%EEt (*D* *_P,PH+_*)	*ΔEE* (%)/*p* value	%EEt (*D* *_P,PH+,P2H+_*)	*ΔEE* (%)/*p* value
**Quinine**	4.0	38.2 ± 6.6	2.4	−93.7/**<10^−5^**	37.1	−2.9/**0.87**	–	–
	6.0	45.7 ± 7.0	2.9	−93.7/**<10^−5^**	54.0	+18.2/**0.23**	–	–
	7.4	62.0 ± 2.7	13.6	−78.1/**<10^−5^**	56.3	−9.2/**0.03**	–	–
	9.0	71.2 ± 5.2	74.3	+4.4/**0.55**	77.8	+9.3/**0.21**	–	–
$\Sigma |∆\mathrm{EE}|/\tilde{∆\mathrm{EE}}$			–	269.9/85.9	–	39.6/9.3	–	
**Primaquine**	4.0	9.5 ± 2.0	2.4	−74.7/**4.0×10^−4^**	39.6	+316.8/<**1×10^−5^**	–	–
	6.0	60.5 ± 7.6	2.5	−95.9/**<10^−5^**	43.0	−28.9/**0.02**	–	–
	7.4	52.0 ± 5.3	3.8	−92.7/**<10^−5^**	43.5	−16.3/**0.11**	–	–
	9.0	55.4 ± 5.9	36.8	−33.6/**1.5×10^−3^**	56.2	+1.4/**0.89**	–	–
$\Sigma |∆\mathrm{EE}|/\tilde{∆\mathrm{EE}}$			–	296.9/83.7	–	363.4/22.6	–	
**Tafenoquine**	4.0	54.0 ± 8.1	2.5	−95.4/**<10^−5^**	61.5	+13.9/**0.35**	–	–
	6.0	71.6 ± 14.8	7.3	−89.8/**1.4×10^−5^**	65.6	−8.4/**0.69**	–	–
	7.4	73.9 ± 8.0	58.0	−21.5/**0.04**	76.3	+3.2/**0.76**	–	–
	9.0	86.1 ± 8.9	98.1	+13.9/**0.18**	98.1	+13.9/**0.18**	–	–
$\Sigma |∆\mathrm{EE}|/\tilde{∆\mathrm{EE}}$			–	220.6/55.7	–	39.4/11.2	–	
**Quinacrine**	4.0	11.3 ± 2.1	2.4	−78.8/**1.7×10^−5^**	2.5	−77.9/**2.1×10^−5^**	14.6	+29.2/**0.12**
	6.0	22.5 ± 6.9	2.4	−89.3/**3.9×10^−3^**	8.0	−64.4/**0.04**	18.8	−16.4/**0.60**
	7.4	63.5 ± 3.5	14.7	−76.9/**<10^−5^**	61.3	−3.5/**0.52**	63.1	−0.6/**0.90**
	9.0	94.8 ± 2.7	97.9	+3.3/**0.25**	98.3	−3.7/**0.20**	98.3	+3.7/**0.20**
$\Sigma |∆\mathrm{EE}|/\tilde{∆\mathrm{EE}}$			–	248.3/77.9	–	149.5/34.1	–	49.9/10.1
**Chloroquine**	4.0	9.2 ± 2.3	2.4	−73.9/**2.9×10^−3^**	2.4	−73.9/**2.9×10^−3^**	14.6	+58.7/**0.02**
	6.0	23.7 ± 4.6	2.4	−89.9/**<10^−5^**	5.1	−78.5/**6.3×10^−5^**	16.6	−30.0/**0.13**
	7.4	45.1 ± 8.3	8.6	−80.9/**1.1×10^−5^**	43.1	−4.4/**0.81**	47.1	+4.4/**0.80**
	9.0	76.1 ± 10.0	95.9	+26.0/**0.04**	96.6	+26.9/**0.04**	96.6	+26.9/**0.04**
$\Sigma |∆\mathrm{EE}|/\tilde{∆\mathrm{EE}}$			–	270.7/77.4	–	183.7/50.4	–	120/28.5

Variations in EE (ΔEE, %) and Z-test p values were obtained when comparing experimental (reference condition) vs. theoretical EE values.

By contrast, lower EEe values (<25%) were obtained for diprotonated-type antimalarials at pH 6.0 (**Figure 3A**), a condition in which Drug^2H+^ is the dominant microspecies reaching molecular abundances >95% (**Figure 2**). A higher ionization state and consequent decrease in lipophilicity for Drug^2H+^ in comparison with Drug^H+^ microspecies would explain such variations in EEe. Increases in QC and CQ EEe in the pH range 6.0 to 9.0 further highlighted the role of Drug^2H+^ to Drug^H+^ conversion in modulating their interaction with LPs (**Figure 3A**). Similarly, the observation of ca. fourfold higher EEe values than those theoretically expected at pH 4.0 hinted at the existence of additional partitioning events involving the effective incorporation of diprotonated microspecies into the LP membrane.

### Antimalarial Drug Partitioning in LPs Simulating RBC-Like Phospholipid Compositions

Significant EEe increases of >30% (**Figure 3B** and **Table S2**) were obtained at pH 7.4 for all antimalarials (except TQ, 8%), in the presence of PS (PC:PS-LPs, −61 ± 1.2 mV, **Table S1**) when compared to neutrally charged lipid bilayers (PC-LPs, −6.49 ± 4.7 mV, **Table S1**). As previously observed for PC-LPs comparing distinct pH values, significantly higher experimental EE values than those theoretically expected according to *D* *_P_* were obtained for all antimalarials when assayed in all three PS-containing lipid formulations: PC:PS-LPs, PC:PS:PE-LPs, and PC:PS:PE:PEG-LPs (**Table 2**). These results additionally revealed the interaction and possible internalization of cationic microspecies into LP bilayers and, particularly, the role of PS negative charge in stabilizing such interactions.

**Table 2:** Experimental vs. theoretical EEs for LP suspensions at pH 7.4 as a function of phospholipid composition and *D* *_P_* *, D* *_P,PH+_* drug distribution models.

Drugs	LP formulation	EEe (%)	%EEt (*D* *_P_*)	*ΔEE* (%)/*p* value	%EEt (*D* *_P,PH+_*)	*ΔEE* (%)/*p* value
**Quinine**	PC	66.7 ± 3.7	13.6	−79.6/**<10^−5^**	66.1	−0.9/**0.86**
	PC:PS	87.6 ± 2.4	13.6	−84.5/**<10^−5^**	87.9	+0.3/**0.91**
	PC:PS:PE	72.4 ± 3.1	13.6	−81.2/**<10^−5^**	70.7	−2.3/**0.59**
	PC:PS:PE:PEG	70.9 ± 4.2	13.6	−80.8/**<10^−5^**	70.7	−0.3/**0.97**
**Primaquine**	PC	51.2 ± 8.2	3.8	−92.6/**<10^−4^**	48.9	−4.5/**0.78**
	PC:PS	86.5 ± 0.9	3.8	−95.6/**<10^−5^**	85.4	−1.3/**0.20**
	PC:PS:PE	77.8 ± 3.4	3.8	−95.1/**<10^−5^**	78.7	+1.2/**0.79**
	PC:PS:PE:PEG	79.6 ± 2.4	3.8	−95.2/**<10^−5^**	78.7	−1.1/**0.70**
**Tafenoquine**	PC	80.1 ± 1.7	58.0	−27.6/**<10^−5^**	74.6	−6.9/**<10^−5^**
	PC:PS	86.6 ± 3.3	58.0	−33.0/**<10^−5^**	82.3	−5.0/**0.18**
	PC:PS:PE	89.8 ± 4.6	58.0	−35.4/**<10^−5^**	88.0	−2.0/**0.70**
	PC:PS:PE:PEG	88.6 ± 4.9	58.0	−34.5/**<10^−5^**	85.4	−3.6/**0.52**
**Quinacrine**	PC	66.9 ± 4.8	14.7	−78.0/**<10^−5^**	66.1	−1.2/**0.86**
	PC:PS	92.3 ± 1.1	14.7	−84.1/**<10^−5^**	91.9	−0.4/**0.70**
	PC:PS:PE	77.3 ± 3.8	14.7	−81.0/**<10^−5^**	78.8	+1.9/**0.70**
	PC:PS:PE:PEG	83.6 ± 3.0	14.7	−82.4/**<10^−5^**	82.2	−1.7/**0.64**
**Chloroquine**	PC	40.9 ± 3.8	8.6	−79.0/**<10^−5^**	43.1	+5.4/**0.57**
	PC:PS	59.4 ± 3.6	8.6	−85.5/**<10^−5^**	58.7	−1.2/**0.84**
	PC:PS:PE	42.8 ± 4.8	8.6	−79.9/**<10^−5^**	43.1	+0.7/**0.94**
	PC:PS:PE:PEG	50.4 ± 4.7	8.6	−82.9/**<10^−5^**	51.8	+2.8/**0.76**

Variations in EE (ΔEE, %) and Z-test p values were obtained when comparing experimental (reference condition) vs. theoretical EE values.

The remarkable drug EEe obtained in LPs simulating the RBC membrane inner leaflet (PC:PS:PE-LPs), which ranged from 60% (CQ) to >86% (rest of drugs), further highlighted the potential role of RBCs as drug carriers for malaria therapy and validated their capacity to stably encapsulate weakly basic antimalarials (**Figure 3B**). Finally, non-significant differences in antimalarial EEes were detected in RBC-like LPs upon surface steric stabilization (PC:PS:PE-LPs versus PC:PS:PE:PEG-LPs, **Figure 3B** and **Table S2**). This observation likely suggested the stable internalization into the LP membrane of ionized drug microspecies, which account for ca. 100% of all microspecies found at pH 7.4 (**Figure 2**), rather than the existence of transitory ionic interactions at the LP surface.

### Determination of Partition Coefficients for Ionized Drug Microspecies in PC-LP Suspensions

**Figure 4:**
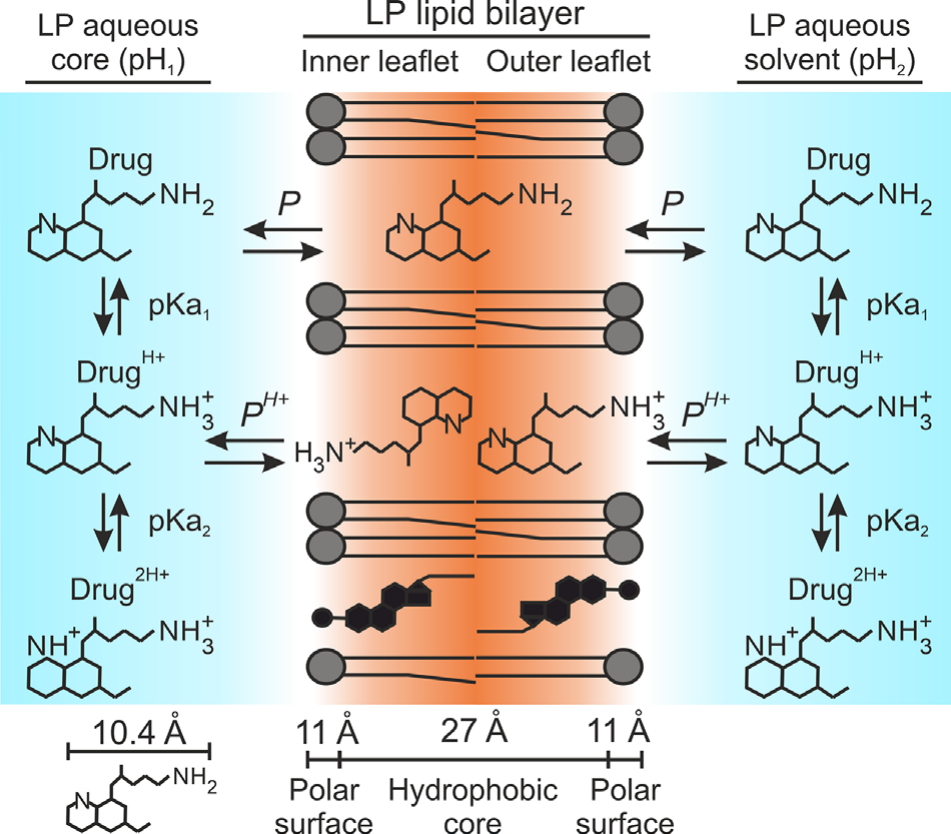
*D_P,PH+_* distribution model. Illustration adapted for amphiphilic diprotic basic drugs in liposomal systems. Primaquine is used as drug example. Dimensions of the bilayer hydrophobic core and associated headgroup polar surface considering 18-carbon PC have been retrieved from previous publications (Petrache et al., 2000; Maurer et al., 2001). Primaquine molecular dimensions were determined using the Chemicalize software developed by ChemAxon Ltd.

The discrepancies observed between EEe and EEt (*D* *_P_*) for PC-LPs (pH 4.0–9.0, **Table 1**) called for the definition of a revised distribution model that envisages the existence of partitioning events for antimalarial drug ionized microspecies in the LP bilayer. As an initial approximation, we first studied the simultaneous participation of *P* (Equation 1) together with a second partition coefficient defined here as *P* *^H+^* and relative to monoprotonated drug microspecies. The resulting *D_P,PH+_* distribution model is represented in **Figure 4**.

**Figure 5:**
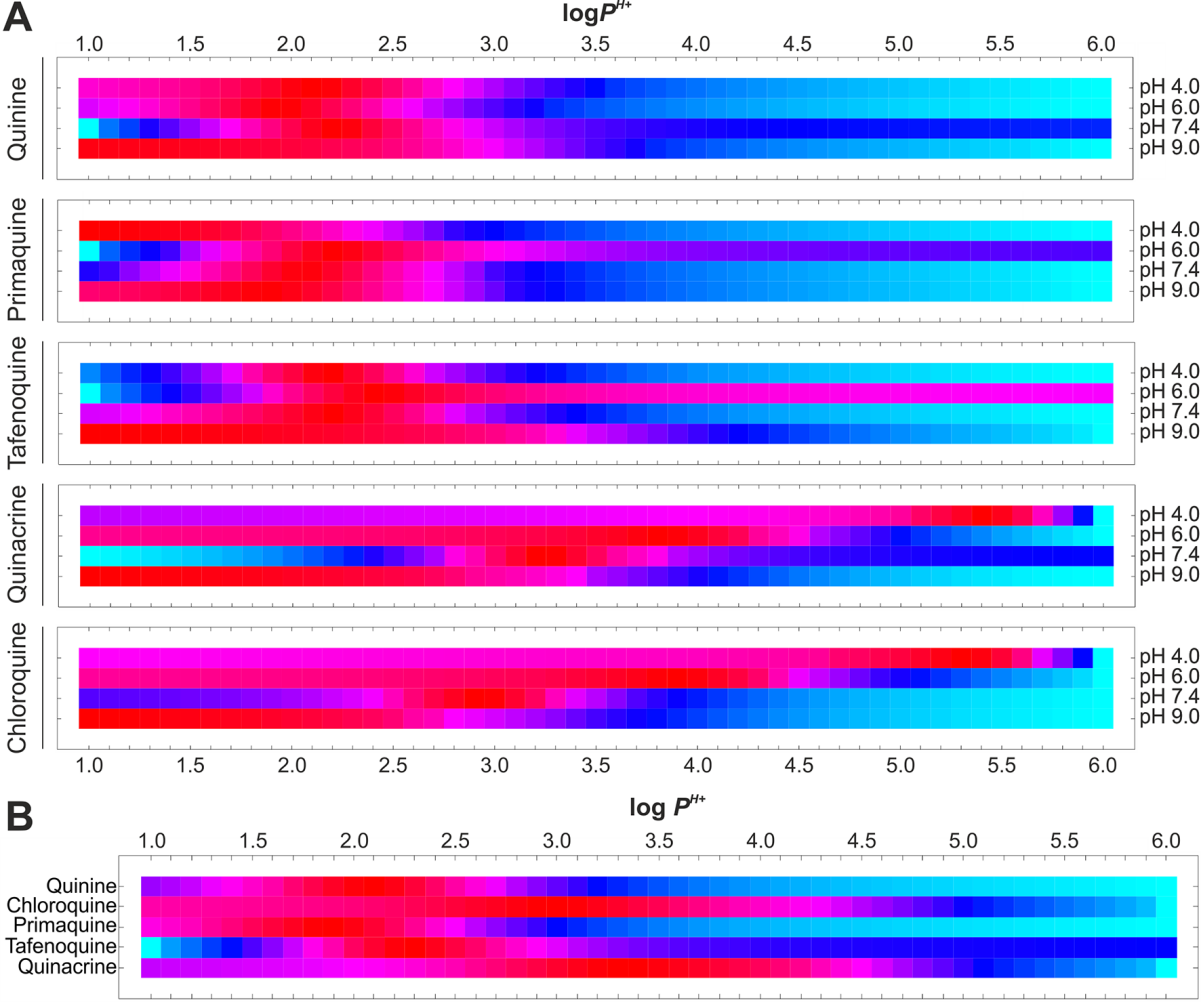
Estimation of antimalarial *P^H+^* coefficient values for PC-based LPs. **(A)** *P^H+^*-dependent drug EEt vs. EEe variances for all experimental pH values studied in accordance with the *D* *_P,PH+_* distribution model. **(B)** Isolation of *P^H+^* coefficient exhibiting least cumulative variances. Normalized variances are individually illustrated for each drug and pH value using the Hue color function (light blue and dark red as maximum and minimum variance levels, respectively).

Given this second distribution model, hypothetical log *P^H+^* values were considered in the range of 1.0 to 6.0 units (0.1 interval) a range comprising 90% of marketed drugs (Mandić, 2014), and the resulting EEt data were calculated as detailed in *Materials and Methods*. Variances in estimated EEt versus EEe were then determined for all pH conditions assayed (**Figure 5A**) and added up for each given *P^H+^* value (**Figure 5B** and Equation 8). Finally, the *P^H+^* value exhibiting least cumulative variance was independently retrieved for each antimalarial (**Table 3** and Equation 9).

(8) $$\begin{array}{c} \mathrm{VAR}(P^{H+})=\Sigma_{\mathrm{pH}〚i〛;\;i=1}^{\mathrm{pH}〚i〛;\;i=4}{\left({\mathrm{EEe}}_{\mathrm{pH}〚i〛}-{\mathrm{EEt}}_{\mathrm{pH}〚i〛}(D_{p,p^{H+}})\right)}^{2};\\ \mathrm{pH}=\langle 4.0,6.0,7.4,9.0\rangle \end{array}$$

(9) $$P^{H+}(\mathrm{Drug})=\underset{4.0\leq P^{H+}\leq 9.0}{\min}\mathrm{VAR}\;(P^{H+})$$

**Table 3:** Summary of antimalarial partitioning coefficients estimated in LP suspensions.

		pH-dependent distribution (PC-LPs)		Phospholipid composition-dependent distribution (log *P* *^H+^*, pH 7.4)			
**Drug**	**log *P***	**log *P*** ***^H+^***	**log *P^2H+^***	**PC**	**PC:PS**	**PC:PS:PE**	**PC:PS:PE:PEG**
Quinine	2.82	2.1	–	2.3	2.9	2.4	2.4
Primaquine	3.20	1.9	–	2.0	2.8	2.6	2.6
Tafenoquine	4.97	2.3	–	2.5	2.7	2.9	2.8
Quinacrine	5.15	3.2	1.2	3.3	4.1	3.6	3.7
Chloroquine	4.72	2.9	1.2	2.9	3.2	2.9	3.0
						RBC-like LPs	

Log P values were determined using the Chemicalize software developed by ChemAxon Ltd., except for CQ and PQ, whose log P has been experimentally determined elsewhere (Omodeo-Salè et al., 2009; Nair et al., 2012).

Calculated log *P* *^H+^* values for all antimalarials ranged between 1.9 and 3.2 units and resulted to be remarkably smaller than their log *P* counterpart, with an average of 1.7 ± 0.7 units lower (**Table 3**). The ionized amino group present in monoprotonated microspecies would be responsible in this case for their reduced lipophilicity and consequent decreased internalization into the LP lipid bilayer. Ionized moieties are nevertheless located away from the hydrophobic regions of aromatic rings and hydrocarbon chains (**Figure 2**) thereby enabling these regions to stably internalize into LP membrane leaflets. Such lipophilic character would be reflected by the modest log *P* *^H+^* values obtained.

The estimated theoretical drug EE values based on the *D* *_P_*_,_ *_PH+_* distribution model, EEt (*D* *_P_*_,_ *_PH+_*), and retrieved *P* *^H+^* are summarized in **Table 1**, and an example of drug microspecies overall abundance for all fractions present in the system is illustrated in **Table S3**. EEt values better fitting EEe data were obtained for all drugs in comparison to the *D*_P_ model (**Table 1**), i.e., $\tilde{∆\mathrm{EE}}$ (*D_P_*) = 55.7–85.9 versus $\tilde{∆\mathrm{EE}}$ (*D_P_*_,_ *_PH+_*) = 9.3–50.4, and especially at pH values ≤7.4, where ionized microspecies are predominantly found, i.e., $\tilde{∆\mathrm{EE}}$ (*D_P_*) = 78.8–93.7 vs. $\tilde{∆\mathrm{EE}}$ (*D* *_P_*_,_ *_PH+_*) = 8.4–30. The best fitting to experimental data with ΔEE <30%, along with an overall ca. 6-fold reduction in $\tilde{∆\mathrm{EE}}$ in comparison to *D* *_P_*, was obtained for monoprotonated-type antimalarials (QN, PQ, TQ), which illustrates the capacity of *D* *_P_*_,_ *_PH+_* to accurately model their distribution in PC-LP suspensions at pH 4.0 to 9.0.

**Figure 6:**
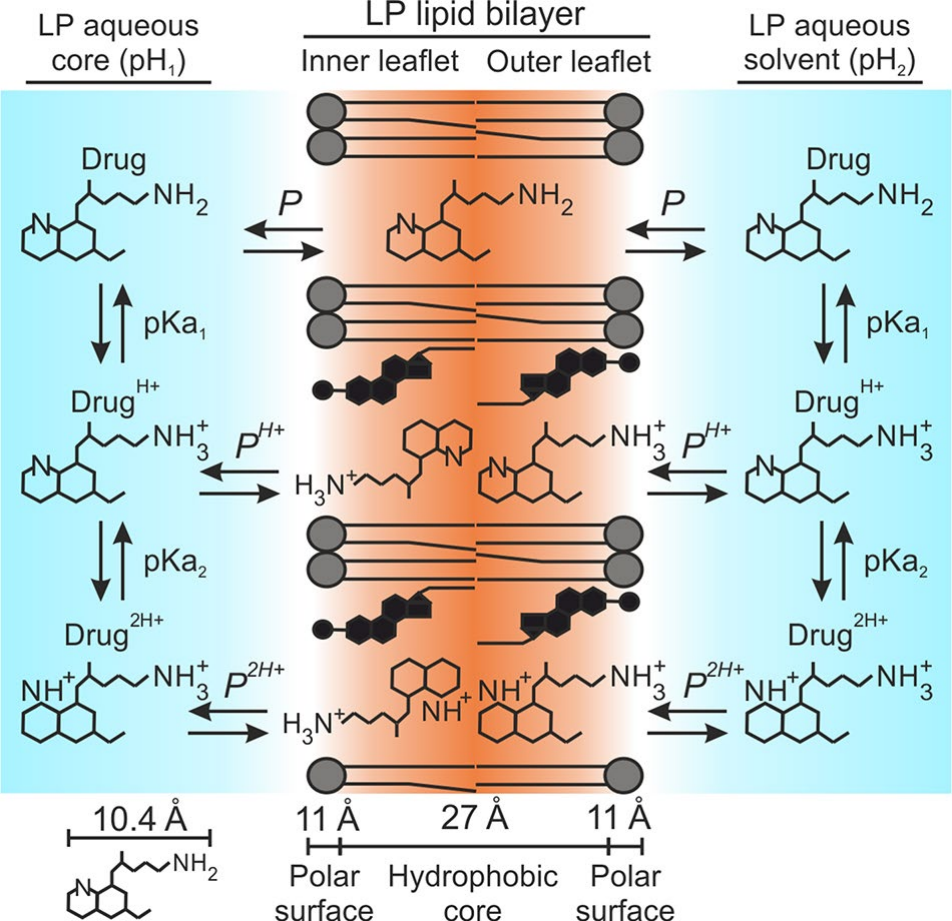
*D* *_P_*_,_ *_PH+_*_,_ *_P2H+_* distribution model. Illustration adapted for amphiphilic diprotic basic drugs in liposomal systems. Primaquine is used as drug example. Dimensions of the bilayer hydrophobic core and associated headgroup polar surface considering 18-carbon PC have been retrieved from previous publications (Petrache et al., 2000; Maurer et al., 2001). Primaquine molecular dimensions were determined using the Chemicalize software developed by ChemAxon Ltd.

Moreover, given the large variations in EEe versus EEt (*D* *_P_*_,_ *_PH+_*) of >64% obtained for diprotonated-type antimalarials (CQ, QC) at pH 4.0 and 6.0 (**Table 1**), an additional partition coefficient, defined here as *P* *^2H+^*, was contemplated describing the partitioning of diprotonated drug microspecies. The resulting *D* *_P_*_,_ *_PH+_*_,_ *_P2H+_* distribution model is represented in **Figure 6**.

**Figure 7:**
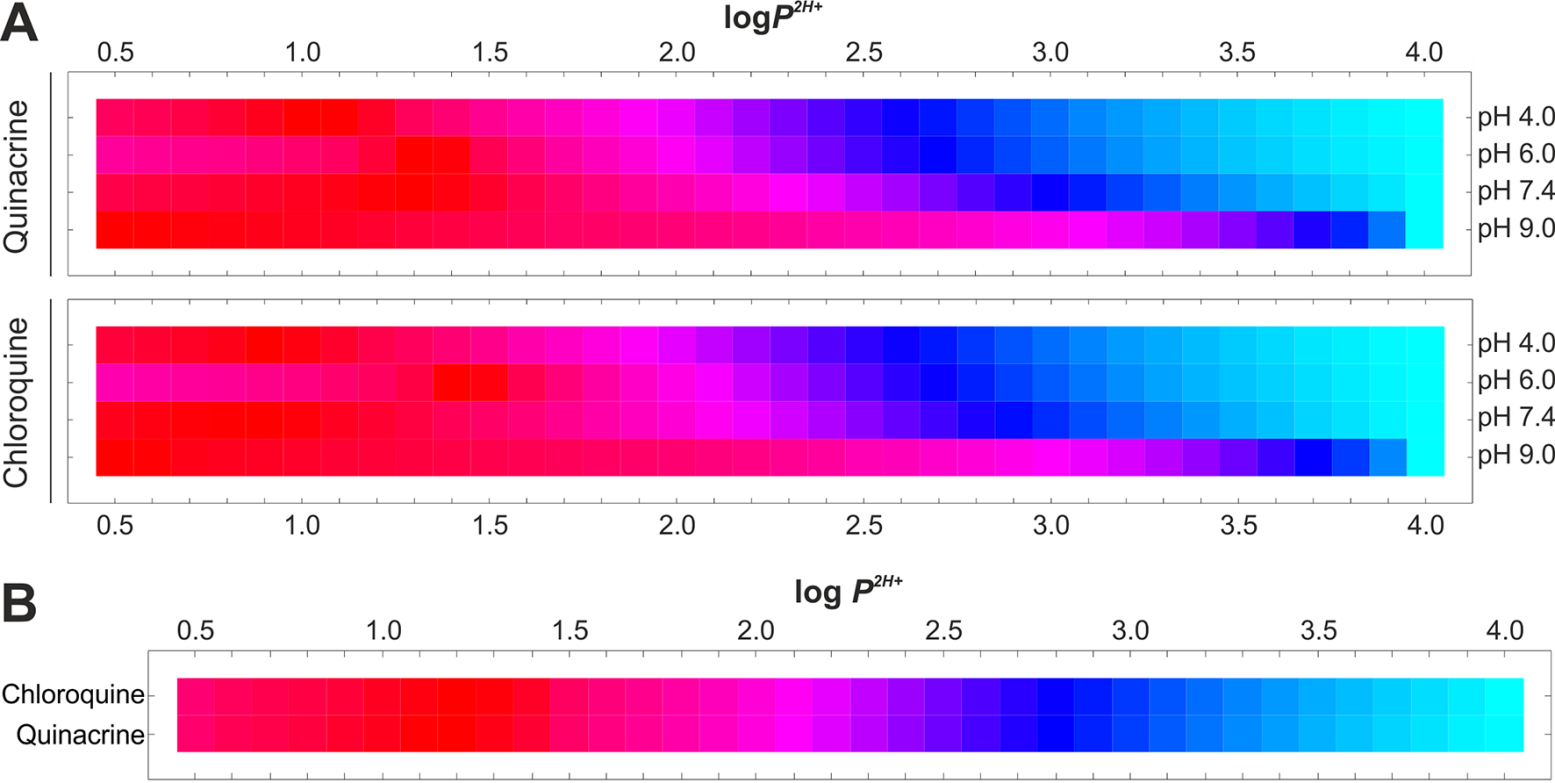
Estimation of *P* *^2H+^* coefficient values for diprotonated-type drugs in PC-based LPs. **(A)** *P* *^2H+^*-dependent drug EEt vs. EEe variances for all experimental pH values studied in accordance with *D* *_P_*_,_ *_PH+_*_,_ *_P2H+_* distribution model. **(B)** Isolation of *P* *^2H+^* coefficient exhibiting least cumulative variances. Normalized variances are individually illustrated for each drug and pH value using the Hue color function (light blue and dark red as maximum and minimum variance levels, respectively).

Similarly to *P* *^H+^* determination, log_10_ values ranging from 0.5 to 4.0 (0.1 interval) were considered for *P* *^2H+^* and the resulting EEt data were calculated as detailed in *Materials and Methods*. Predefined *P* *^H+^* values were maintained constant for EEt calculation. EEe versus EEt variances were determined for all pH conditions assayed (**Figure 7A**), subsequently added up for each given *P* *^2H+^* value (**Figure 7B** and Equation 10), and the *P* *^2H+^* value exhibiting least cumulative variance was individually retrieved for CQ and QC (**Table 3** and Equation 11). Calculated *P* *^2H+^* values (1.2 log_10_ units) were considerably lower than their associated *P* and *P* *^H+^* coefficients, a result that suggests the likely impaired solubility of diprotonated microspecies in the LP membrane due to their doubly ionized state and the consequent reduction in drug nonpolar surface area.

(10) $$\begin{array}{c} \mathrm{VAR}(P^{2H+})=\Sigma_{\mathrm{pH}〚i〛;\;i=1}^{\mathrm{pH}〚i〛;\;i=4}{\left({\mathrm{EEe}}_{\mathrm{pH}〚i〛}-{\mathrm{EEt}}_{\mathrm{pH}〚i〛}(D_{p,p^{H+},p^{2H+}})\right)}^{2};\\ \mathrm{pH}=\langle 4.0,6.0,7.4,9.0\rangle \end{array}$$

(11) $$P^{2H+}\left(\mathrm{Drug}\right)=\underset{0.5\leq P^{2H+}\leq 4.0}{\min}\mathrm{VAR}\;(P^{2H+})$$

Theoretical EE data for diprotonated-type antimalarials obtained according to the *D* *_P_*_,_ *_PH+_*_,_ *_P2H+_* distribution model, EEt (*D* *_P_*_,_ *_PH+_*_,_ *_P2H+_*), are summarized in **Table 1**. **Table S4** illustrates an example of CQ microspecies overall partitioning in our LP system. Using this model, an improved fitting to EEe data was obtained with respective ca. threefold and fivefold reductions in $\tilde{∆\mathrm{EE}}$ when compared to the *D* *_P_*_,_ *_PH+_* and *D* *_P_* distribution models (**Table 1**). Furthermore, the *D* *_P_*_,_ *_PH+_*_,_ *_P2H+_* model resulted in a better approximation to EEe data at pH ≤6.0 when compared to previous distribution models, i.e., $\tilde{∆\mathrm{EE}}$ (*D* *_P_*) = 81.9–84.1 versus $\tilde{∆\mathrm{EE}}$ (*D* *_P_*_,_ *_PH+_*) = 71.1–76.2 versus $\tilde{∆\mathrm{EE}}$ (*D* *_P_*_,_ *_PH+_*_,_ *_P2H+_*) = 22.8–44.3. The largest ΔEE of 58.7% was found for CQ at pH 4.0. Such improved fitting to EEe data provided by *D* *_P_*_,_ *_PH+_*_,_ *_P2H+_* evidences the important and combined role of both *P* *^H+^* and *P* *^2H+^* coefficients to more accurately model the distribution of diprotic basic antimalarials in LP suspensions.

### Determination of Partition Coefficients for Ionized Drug Microspecies in RBC-Like LPs

**Figure 8:**
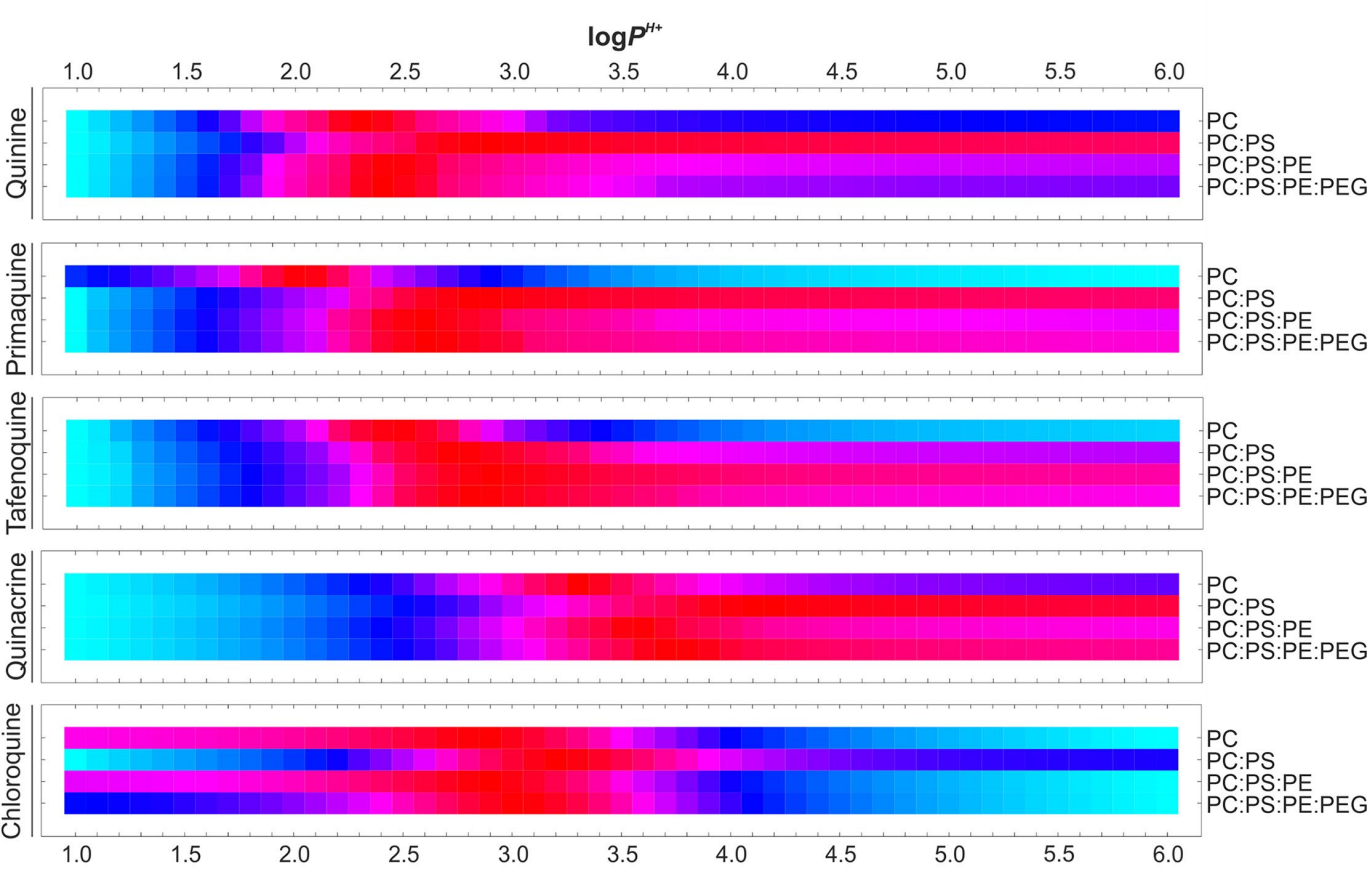
Estimation of antimalarial *P* *^H+^* coefficient values for RBC-like LPs. *P* *^H+^*-dependent drug EEt vs. EEe variances are illustrated for all the experimental phospholipid compositions studied in accordance with *D* *_P_*_,_ *_PH+_* distribution model. Normalized variances are individually illustrated for each drug and lipid composition using the Hue color function (light blue and dark red as maximum and minimum variance levels, respectively).

Additional partition coefficients were determined aiming to better describe the interaction of antimalarial drug ionized microspecies in physiological conditions with LP suspensions that contain RBC-like phospholipid compositions. To do so, *D* *_P_*_,_ *_PH+_* was considered as simplest distribution model of reference given its previously demonstrated capacity to model EEe data for all studied antimalarials at pH 7.4 (ΔEE = 3.5–16.3, **Table 1**). Analogously to *P* *^H+^* estimation related to pH changes, we considered log *P* *^H+^* values in the range of 1.0 to 6.0 units (0.1 interval), followed by EEt calculation as explained in *Materials and Methods*. EEt versus EEe variances were subsequently calculated for all lipid compositions studied (**Figure 8**) and the single least variant *P* *^H+^* value was finally extracted for each antimalarial (**Table 3** and Equations 12–13).

(12) Lipid composition=〈PC,PC/PS,PC/PS/PE,PC/PS/PE/PEG〉

(13) $$\begin{array}{l} P^{2H+}(\mathrm{Lipid composition}〚i〛)=\\ \underset{1.0\leq p^{H+}\leq 6.00}{\min}{\left({\mathrm{EEe}}_{\mathrm{Lipid composition}〚i〛}-\mathrm{EEt}(D_{p,p^{H+}})\right)}^{2} \end{array}$$

In an analogous manner to EEe data, a positive correlation between *P* *^H+^* and the presence of PS was observed for all antimalarials, with mean log *P* *^H+^* of 2.88 to 3.14 and 2.60 for all PS-containing LPs and PC-LPs, respectively (**Table 3**). These results are in accordance with the suggested role of PS in increasing the accumulation of cationic drug molecules in lipid bilayers. Overall decreases in ca. 1.4- and 1.6-fold log_10_ units were furthermore noticed for *P* *^H+^* in, respectively, all PS-containing LPs and PC-LPs when compared to drug-associated *P* values, which reflects again the function of PS in regulating antimalarial partitioning along with the effect of drug ionized groups on diminishing their stable internalization into LPs. Moreover, *P* *^H+^* small variations of <0.5 log_10_ units were obtained between PS-LPs and RBC-like LPs (**Table 3**), which could be caused by the increased cholesterol amounts present in the latter formulations along with the consequent reduced number of total phospholipid molecules. Undetectable differences in log *P* *^H+^*(<0.1 units) were obtained after LP steric stabilization (PC:PS:PE:PEG-LPs versus PC:PS:PE-LPs).

In summary, the definition of *D* *_P_*_,_ *_PH+_*, together with the estimation of *P* *^H+^* coefficients relative to phospholipid composition allowed for the calculation of EEt values properly fitting experimental data (**Table 2**). Non-significant differences (*p* value <0.05) in EEe versus EEt (*D* *_P_*_,_ *_PH+_*) were obtained in this regard for all antimalarials, with ΔEE ≤7% in all conditions assayed. All together, these results importantly stress the role of lipid composition and phospholipid charge in modulating the partitioning of polyprotic drugs in liposomal systems, as well as the potential role of RBCs as supercarriers for weakly basic antimalarials, which might potentially accumulate within the cell membrane inner leaflet as ion pair in association with PS.

### Determination of Antimalarial Drug Distribution Coefficient Based on *D* *_P_*_,_ *_PH+_* and *D* *_P_*_,_ *_PH+_*_,_ *_P2H+_*

**Figure 9:**
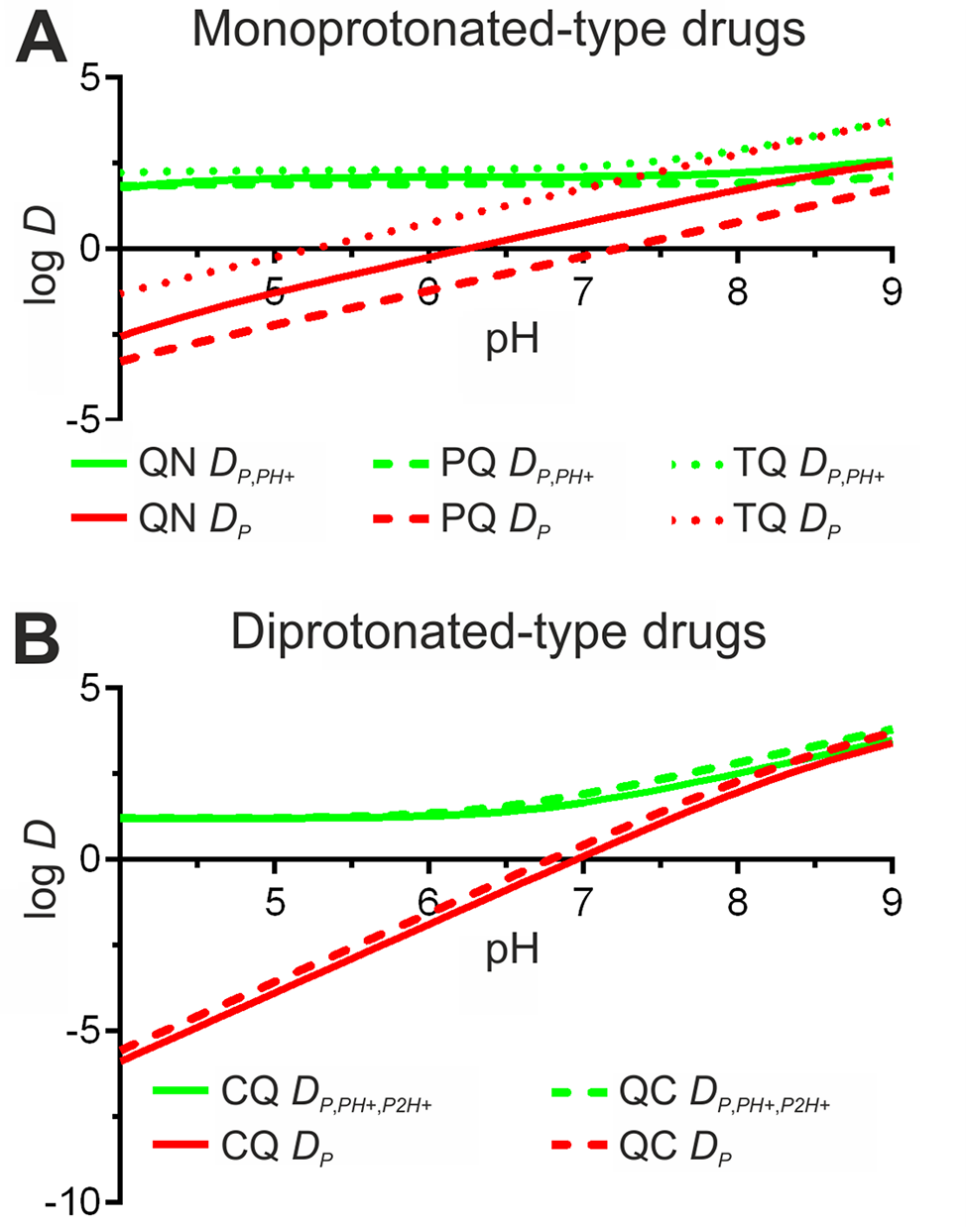
Reformulation of antimalarial *D* coefficient for PC-LPs according to the new drug distribution models reported here. The original *D*_P_ model is included for comparison. **(A)** Monoprotonated-type drugs quinine (QN), primaquine (PQ), and tafenoquine (TQ). **(B)** Diprotonated-type antimalarials chloroquine (CQ) and quinacrine (QC).

The new distribution models reported here enabled the reformulation of the initially considered *D* coefficient (Equations 2–5) to properly represent our experimental EE data. Modified *D* coefficients in accordance with *D* *_P_*_,_ *_PH+_* and *D* *_P_*_,_ *_PH+_*_,_ *_PH+_* models, i.e., *D*(*P*,*P* *^H+^*) and *D*(*P*,*P* *^H+^*,*P* *^2H+^*), Equations 14–17, were calculated for the pH range 4.0 to 9.0, using the previously estimated *P* *^H+^* and *P* *^2H+^* values relative to PC-LP suspensions (**Figure 9**). As expected, a sustained reduction in *D* was obtained when lowering pH, as a reflection of the increased drug ionization state and consequently reduced lipophilic character. In more detail, a drastic fall in *D* reaching negative log_10_ values below pH 7.0 (PQ, CQ, QC), pH 6.0 (QN), and pH 5.0 (TQ), was calculated for *D* *_P_*, whereas the application of *D* *_P_*_,_ *_PH+_* and *D* *_P_*_,_ *_PH+_*_,_ *_P2H+_* alternative models provided positive log *D* values still maintained below pH 4.0. Such differences in antimalarial lipophilicity in acidic conditions would be expected as a result of the predicted capacity of drug ionized microspecies in *D* *_P_*_,_ *_PH+_* and *D* *_P_*_,_ *_PH+_*_,_ *_P2H+_* models to stably internalize into the LP lipid bilayer.

(14) $$\begin{array}{c} D\;(P,P^{H+})=\left(\frac{{\mathrm{mol Drug}}_{\mathrm{org}}\times {\mathrm{Vol}}_{\mathrm{aq}}}{{\mathrm{Vol}}_{\mathrm{org}}\times {\mathrm{mol Drug}}_{\mathrm{aq}}}+\frac{{\mathrm{mol Drug}}_{\mathrm{org}}^{H+}\times {\mathrm{Vol}}_{\mathrm{aq}}}{{\mathrm{Vol}}_{\mathrm{org}}\times {\mathrm{mol Drug}}_{\mathrm{aq}}}\right)/\\ \;\left(1+\frac{{\mathrm{mol Drug}}_{\mathrm{aq}}^{H+}}{{\mathrm{mol Drug}}_{\mathrm{aq}}}+\frac{{\mathrm{mol Drug}}_{\mathrm{org}}^{2H+}}{{\mathrm{mol Drug}}_{\mathrm{aq}}}\right) \end{array}$$

(15) $$\begin{array}{l} \log D\;(P,P^{H+})=\log\left(P+\left(P^{H+}\times 10^{\left({\mathrm{pKa}}_{1}-\mathrm{pH}\right)}\right)\right)-\log(1+10^{\left({\mathrm{pKa}}_{1}-\mathrm{pH}\right)})\\ +\;10^{\left({\mathrm{pKa}}_{1}+{\mathrm{pKa}}_{2}-2\times \mathrm{pH}\right)}) \end{array}$$

(16) $$\begin{array}{l} D(P,P^{H+},P^{2H+})=\\ \left(\frac{{\mathrm{mol Drug}}_{\mathrm{org}}\times {\mathrm{Vol}}_{\mathrm{aq}}}{{\mathrm{Vol}}_{\mathrm{org}}\times {\mathrm{mol Drug}}_{\mathrm{aq}}}+\frac{{\mathrm{mol Drug}}_{\mathrm{org}}^{H+}\times {\mathrm{Vol}}_{\mathrm{aq}}}{{\mathrm{Vol}}_{\mathrm{org}}\times {\mathrm{Vol}}_{\mathrm{aq}}}+\frac{{\mathrm{mol Drug}}_{\mathrm{org}}^{2H+}\times {\mathrm{Vol}}_{\mathrm{aq}}}{{\mathrm{Vol}}_{\mathrm{org}}\times {\mathrm{mol Drug}}_{\mathrm{aq}}}\right)/\\ \left(1+\frac{{\mathrm{mol Drug}}_{\mathrm{aq}}^{H+}}{{\mathrm{mol Drug}}_{\mathrm{aq}}}+\frac{{\mathrm{mol Drug}}_{\mathrm{aq}}^{2H+}}{{\mathrm{mol Drug}}_{\mathrm{aq}}}\right) \end{array}$$

(17) $$\begin{array}{l} \log D\;(P,P^{H+},P^{2H+})=\log(P+(P^{H+}\times 10^{\left({\mathrm{pKa}}_{1}-\mathrm{pH}\right)})+(P^{2H+}\times \\ 10^{\left({\mathrm{pKa}}_{1}+{\mathrm{pKa}}_{2}-2\times \mathrm{pH}\right)}))-\log(1+10^{\left({\mathrm{pka}}_{1}-\mathrm{pH}\right)}+10^{\left({\mathrm{pKa}}_{1}+{\mathrm{pKa}}_{2}-2\times \mathrm{pH}\right)}) \end{array}$$

### Modeling Antimalarial Drug EE in Response to Transmembrane pH Gradients

**Figure 10:**
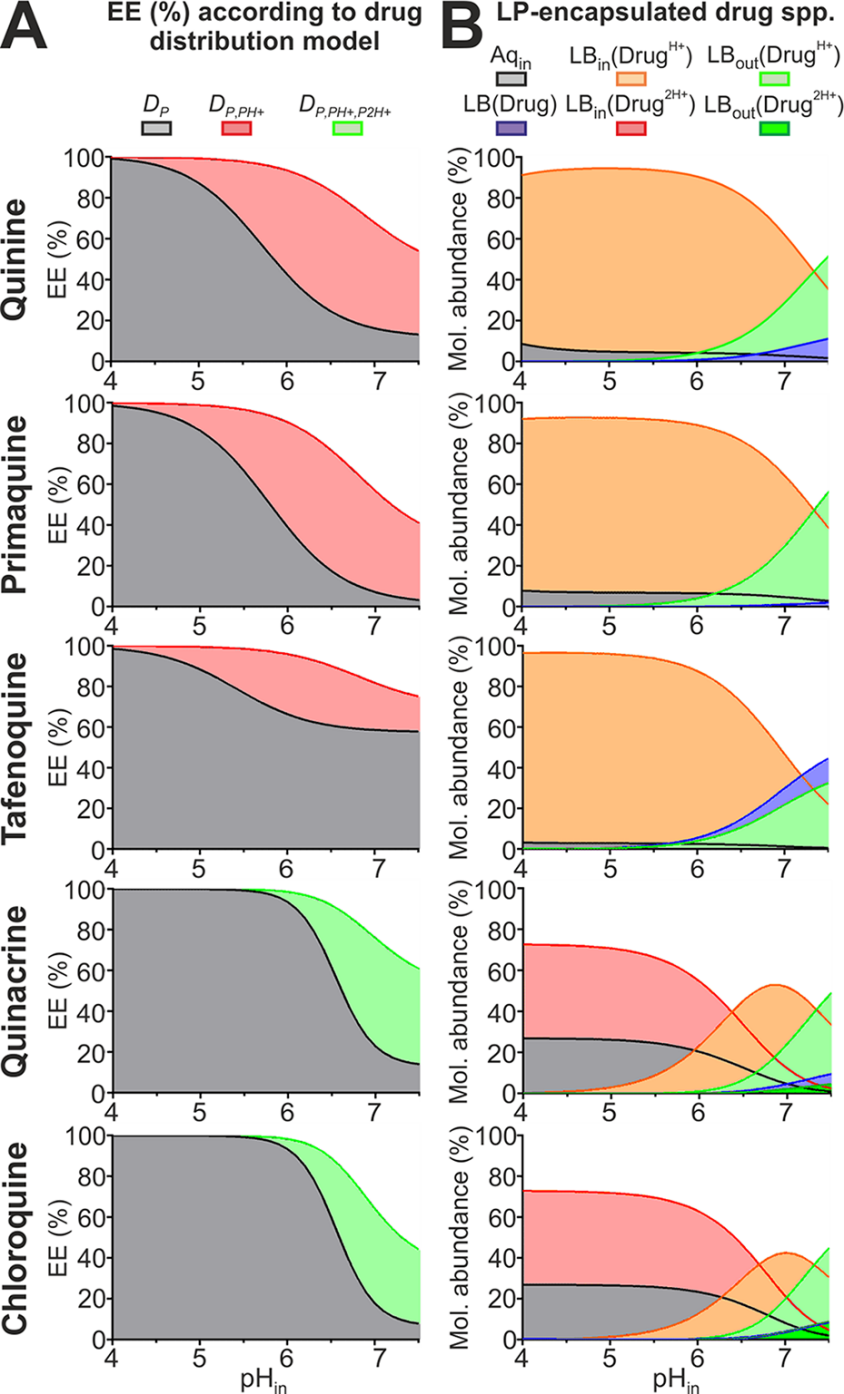
Modeling antimalarial EEs and intravesicular distribution for PC-LP suspensions in response to pH gradients. **(A)** EEs calculated considering all distribution models studied in this work for 10 mM lipid particles containing internal pH 4.0 to 7.4 values and suspended in aqueous solution at pH 7.4. **(B)** Molecular abundances for all monoprotonated- and diprotonated-type antimalarial encapsulated microspecies (spp.) according to, respectively, *D* *_P_*_,_ *_PH+_* and *D* *_P_*_,_ *_PH+_*_,_ *_P2H+_* distribution models. Drug spp. and intravesicular fractions comprise: (i) all spp. located inside the LP aqueous core (Aq_in_), (ii) monoprotonated (Drug^H+^) and diprotonated (Drug^2H+^) ionized spp. incorporated into outer and inner lipid bilayer leaflets (LB_out_, LB_in_), and (iii) membrane-solubilized unionized spp., LB(Drug).

Considering our previously reported use of a transmembrane pH 4.0_in_ to 7.4_out_ gradient for the active encapsulation of diprotic antimalarials into RBC-targeted PC-based LPs (Moles et al., 2015; Moles et al., 2017), we evaluated a possible application of the novel *D* *_P_*_,_ *_PH+_* and *D* *_P_*_,_ *_PH+_*_,_ *_P2H+_* distribution models in estimating drug EE and intravesicular distribution in LPs as a function of their internal pH (pH_in_ range, 4.0–7.4). Modeled EE (**Figure 10A**) along with drug microspecies molecular abundance within LP fractions (lipid bilayer leaflets and aqueous core, **Figure 10B**), were calculated considering 10 mM PC-LP suspensions at pH 7.4 together with estimated drug *P* *^H+^* and *P* *^2H+^* values (**Table 3**). An exemplification of microspecies overall abundance for all fractions present in the system is illustrated in **Table S5**.

Given the lipophilic character evidenced here for antimalarial ionized microspecies, larger EEs were predicted for *D* *_P_*_,_ *_PH+_* and *D* *_P_*_,_ *_PH+_*_,_ *_P2H+_* models when compared to *D* *_P_* (**Figure 10A**). Such differences were more pronounced for pH_in_ >4.0, and particularly in the range of pH 5.0 to 7.4, where monoprotonated microspecies abound (**Figure 2**). Drug EE ≥90% was estimated to be maintained up to pH_in_ 6.4 when following *D* *_P_*_,_ *_PH+_* and *D* *_P_*_,_ *_PH+_*_,_ *_P2H+_*, with >70% drug molecules being predicted to localize at the LP bilayer (**Figure 10B**). Ionized microspecies, in particular monoprotonated molecules, were estimated to be the dominant form in the LP bilayer at pH_in_ ≤7.4 with abundances reaching >50%.

The new distribution models further led to the hypothesis that whereas only minor amounts (<10%) of ionized microspecies are exposed at the LP surface at pH_in_ ≤6.0, a slight pH_in_ increase to 7.4 triggers their massive exposure to the extraliposomal environment reaching abundances of >50%. Such increase in LP internal pH could be triggered during *in vivo* conditions by the sustained leakage of the encapsulated buffering agent and/or as result of membrane destabilization by plasma components, as already demonstrated in the literature (Allen and Cleland, 1980; Silvander et al., 1998; Russell et al., 2018) and evidenced in our previous publications (Moles et al., 2015; Moles et al., 2017). Exposed antimalarial molecules would then become rapidly exchanged with other lipid bilayers found in solution (Moles et al., 2016; Moles et al., 2017), which represents an attractive operating mechanism for delivering drugs to cells lacking endocytic mechanisms and intracellular vesicle trafficking such as RBCs (Ji et al., 2011).

### Modeling Antimalarial Drug EE and Distribution in RBC-Like Vesicular Systems

In view of our dual purpose of using RBCs as nanotherapeutic target and ultimate vascular drug carrier against blood-borne pathogens (Anselmo et al., 2013; Moles et al., 2015; Moles et al., 2017), we envisaged the application of *D* *_P_*_,_ *_PH+_* and *D* *_P_*_,_ *_PH+_*_,_ *_P2H+_* to model antimalarial drug encapsulation and distribution within RBC fractions (plasma membrane and cytoplasm) at physiological pH 7.4. For this purpose, we considered a RBC-like vesicular system simulating the human RBC dimensions, its asymmetric lipid composition (100% PC and 67:33 PC:PS as phospholipid composition for outer and inner membrane leaflets, respectively), and a typical human blood hematocrit, as detailed in *Materials and Methods*. A similar vesicular system but entirely composed of PC, and therefore displaying an absence of lipid charge asymmetry, was considered for comparison. These vesicular systems are referred here as PC/PS and PC, and an example reproducing CQ distribution in PC/PS is reported in **Table S6**.

EEs exceeding 50% were predicted for all antimalarials in both PC and PC/PS systems (**Table 4**), which encourages once more the utilization of RBCs as effective drug carrier for polyprotic basic drugs (Muzykantov, 2010; Villa et al., 2016). Larger EEs were estimated in the presence of lipid charge asymmetry, with 4% to 6% increases for TQ and CQ and up to 20% to 30% for QN, PQ and QC, a result in good correlation with the previously noticed superior *P* *^H+^* values in PS-containing lipid mixtures (**Table 3**).

**Table 4:** Modeling antimalarial EEs and distribution in RBC-like PC/PS versus PC vesicular systems.

			% Drug molecule abundance within RBC fractions			
**Antimalarial**	**Vesicular system**	**EE (%)**	**Drug (LB)**	**Drug^+^ (LB_out_)**	**Drug^+^ (LB_in_)**	**Total molecules Aq_in_**
Quinine	PC	57.5	3.5	23.8	23.8	49.0
	PC/PS	69.8	2.0	13.9	55.4	28.7
	Δ (%)	+21.4	−42.3	−41.7	+132.7	−41.6
Primaquine	PC	50.0	0.6	16.4	16.4	66.4
	PC/PS	65.2	0.3	8.7	55.5	35.4
	Δ (%)	+30.4	−48.9	−46.7	+238.6	−46.6
Tafenoquine	PC	68.7	22.3	23.7	23.7	30.3
	PC/PS	71.4	19.5	20.9	33.1	26.6
	Δ (%)	+3.9	−12.6	−12.0	+39.3	−12.1
Quinacrine	PC	57.5	4.0	23.5	23.5	49.0
	PC/PS	75.3	1.7	10.5	66.0	21.8
	Δ (%)	+31.0	−56.8	−55.3	+181.1	−55.6
Chloroquine	PC	48.1	2.7	12.9	12.9	71.5
	PC/PS	51.1	2.3	11.4	22.7	63.4
	Δ (%)	+6.2	−13.1	−11.9	+76.1	−11.3

EE was calculated considering a 40% v/v suspension of vesicles simulating RBC dimensions in pH 7.4 solution. Antimalarial microspecies (spp.) and fractions studied included ionized (Drug+) molecules internalized into lipid bilayer inner (LBin) and outer (LBout) leaflets, unionized molecules (Drug) distributed throughout both bilayer leaflets (LB), and all antimalarial molecules retained inside the RBC aqueous core/cytoplasm (Aqin). The percentual increase (+) or decrease (−) in drug EE and spp. molecular abundance between PC (reference condition) and PC/PS vesicular systems is indicated as Δ (%).

Moreover, when looking at the distribution of antimalarials within RBC fractions, a clear shift in QN, PQ, and QC molecular abundance was determined for PC versus PC/PS vesicular systems, with drugs preferentially accumulating in the RBC cytoplasm (49–66% of all drug molecules) and the plasma membrane leaflets (65–78% of all drug molecules), respectively (**Table 4**). By contrast, TQ was found to be concentrated mostly within membrane leaflets in ≥70% and CQ exhibited a cytoplasmic preference (>63% molecules) regardless of the vesicular system considered. Additionally, the presence of lipid charge asymmetry in PC/PS resulted in two major compelling changes when compared to PC that encourage the use of RBCs as drug carriers: a remarkable boost in drug abundance within the plasma membrane inner leaflet (increases of 133–238% for QN, PQ and QC, and of 40–76% for TQ and CQ) and a mild decrease in the number of drug molecules exposed at the RBC surface (reductions of 41–55% for QN, PQ and QC, and of 12% for TQ and CQ).

Based on the distribution data obtained for the RBC-like PC/PS vesicular model and considering a clinical scenario, all diprotic basic antimalarials studied in this work are predicted to largely accumulate in circulating RBCs upon their intravenous administration (>50% EEs, **Table 4**). A preferential distribution would then be expected to take place within either the cell aqueous core (CQ), and/or the internal side of the RBC membrane (QN, PQ, QC, and to a lesser extent, TQ), whereas minor amounts of drugs would remain exposed at the cell surface. This particular subcellular distribution is attractive because it would contribute to preventing the easy exchange of drug molecules with circulating structures (e.g., other cells and high-/low-density lipoproteins) (Fahr et al., 2005; Hefesha et al., 2011; Loew et al., 2011), as well as a potential loss of drug function due to its degradation in both extracellular (blood plasma and body tissues) and cytoplasmic aqueous environments (Waterman et al., 2002). Our models omit the participation of other circulating organic bodies and particles that are known to affect drug distribution *in vivo*, such as plasma lipoproteins and albumin aggregates (Yamasaki et al., 2013; Sobansky and Hage, 2014), though point out at the remarkable potential of RBCs as vascular carriers and prompt the utilization of *ex vivo* RBC loading techniques for drug delivery-based therapeutics (Zhou et al., 2010; Biagiotti et al., 2011; Villa et al., 2016).

## Conclusion

Based on pH- and lipid composition-dependent experimental encapsulation data acquired using liposomal systems, we have theorized here novel distribution models (*D_P,PH+_* and *D_P,PH+,P2H+_*) that precisely describe the partitioning behavior of diprotic basic drugs in PC-based and RBC-analogous LP suspensions. Partition coefficients relative to monoprotonated (*P^H+^*) and diprotonated (*P^2H+^*) microspecies have been estimated in a simple and highly translatable manner for several diprotic antimalarials of clinical significance, for which theoretical EEs have been retrieved *in silico* closely fitting experimental data (non-significant differences were obtained in >75% of all pH/lipid composition conditions studied).

It is important to note that the distribution models, methods, and resulting partition coefficients have been determined for and are therefore applicable to diprotic drugs with differences in pK_a1_ versus pK_a2_ of >2 log_10_ units and to LP suspensions at a maximum of 1:40 drug:lipid ratio and within a pH range of 4.0 to 9.0 units. It is expected that higher drug:lipid ratios can lead to a saturation and consequent disturbance of the lipid bilayer structure by the supplemented drug with ultimate effects on altering LP morphology, membrane charge, size, and overall drug partition behavior. pH values below/above 4.0/9.0 units can additionally alter the lipid bilayer structure and overall particle charge due to phospholipid ionization at such strongly acidic/basic conditions. However, the reported approach has the potential to be adapted to study the distribution of both polyprotic acids and bases in any type of lipid-based vesicular system, and can be furthermore extended to polyprotic drugs with more than two acid dissociation constants.

Our data importantly stress the role of lipid composition and phospholipid charge in modulating the interaction of water-soluble polyprotic drugs with lipid bilayers as well as the remarkable potential of RBCs as vascular drug carriers against blood diseases. The results and models presented here further hint at the stable internalization of ionized drug microspecies into neutrally charged and anionic lipid bilayer leaflets as suggested in other works (de Souza Santos et al., 2014; Barroso et al., 2015), and likely in the latter in the form of ion pairs in association with anionic lipids.

Potential therapeutic applications for the distribution models and partition coefficients reported here include the accurate design of LP-based controlled drug delivery strategies relying on pH gradients, the study of RBCs as clinically safe long-circulating carrier for polyprotic drugs, and the optimization of antimalarial therapies using RBC-targeted liposomal drug formulations (Moles and Fernàndez-Busquets, 2015; Moles et al., 2015, Moles et al., 2017). The approach and methods reported here have demonstrated to accurately fit experimental encapsulation data and aim at serving as an experimental tool for researchers from different scientific disciplines. Our models can be nevertheless improved to better describe the distribution of ionized drugs and variations in local pH at the lipid–water interface, particularly in the case of ionized bilayers, by means of incorporating concepts from the classic Gouy–Chapman model and novel theories (Shapovalov and Brezesinski, 2006). A further optimization of the vesicular model presented here is required for a more accurate representation of a clinical scenario. Plasma components are known to interact with circulating drugs (Yamasaki et al., 2013; Sobansky and Hage, 2014), and these additionally present large biodistribution volumes broadly diffusing across animal tissues.

## Data Availability

All datasets generated for this study are included in the manuscript and the **Supplementary files**.

## Author Contributions

EM conceived the study, designed the methodology, and wrote the manuscript. XF-B acquired funding, contributed to resources, and supervised the study. MK contributed to resources and supervised the study.

## Funding

This work was supported by (i) *Ministerio de Ciencia, Innovación y Universidades*, Spain, grant numbers RTI2018-094579-B-I00 and PCIN-2017-100, which included FEDER funds, (ii) ERA-NET Cofund EURONANOMED, grant number 2017-178 (NANOpheles), and (iii) Generalitat de Catalunya, Spain, grant number 2017-SGR-908.

## Conflict of Interest Statement

The authors declare that the research was conducted in the absence of any commercial or financial relationships that could be construed as a potential conflict of interest.

## Supplementary Material

Additional figures (**Figures S1**-**S4**; reporting UV-visible absorption spectra used for drug quantification, along with illustrations summarizing the calculations and schemes used to determine EEt and drug microspecies abundance in liposomal fractions), tables (**Tables S1**-**S6**; including LP ζ-potential data, variations in experimental versus theoretical EE data, along with detailed drug distribution examples), and an Appendix section including the mathematical algorithms used in this work, all providing supporting information to the results presented in this manuscript, have been supplied into a single PDF file as **Supplementary Material**.
