# Supplementary material for: Modeling the Distribution of Diprotic Basic Drugs in Liposomal Systems: Perspectives on Malaria Nanotherapy
Source: Front Pharmacol. 2019 Sep 25;10:1064. doi: 10.3389/fphar.2019.01064 (PMC6773836; doi:10.3389/fphar.2019.01064)
Supplement: Supplementary file 1 [file DataSheet_1.pdf]

Supplementary Material

1. Supplementary Figures

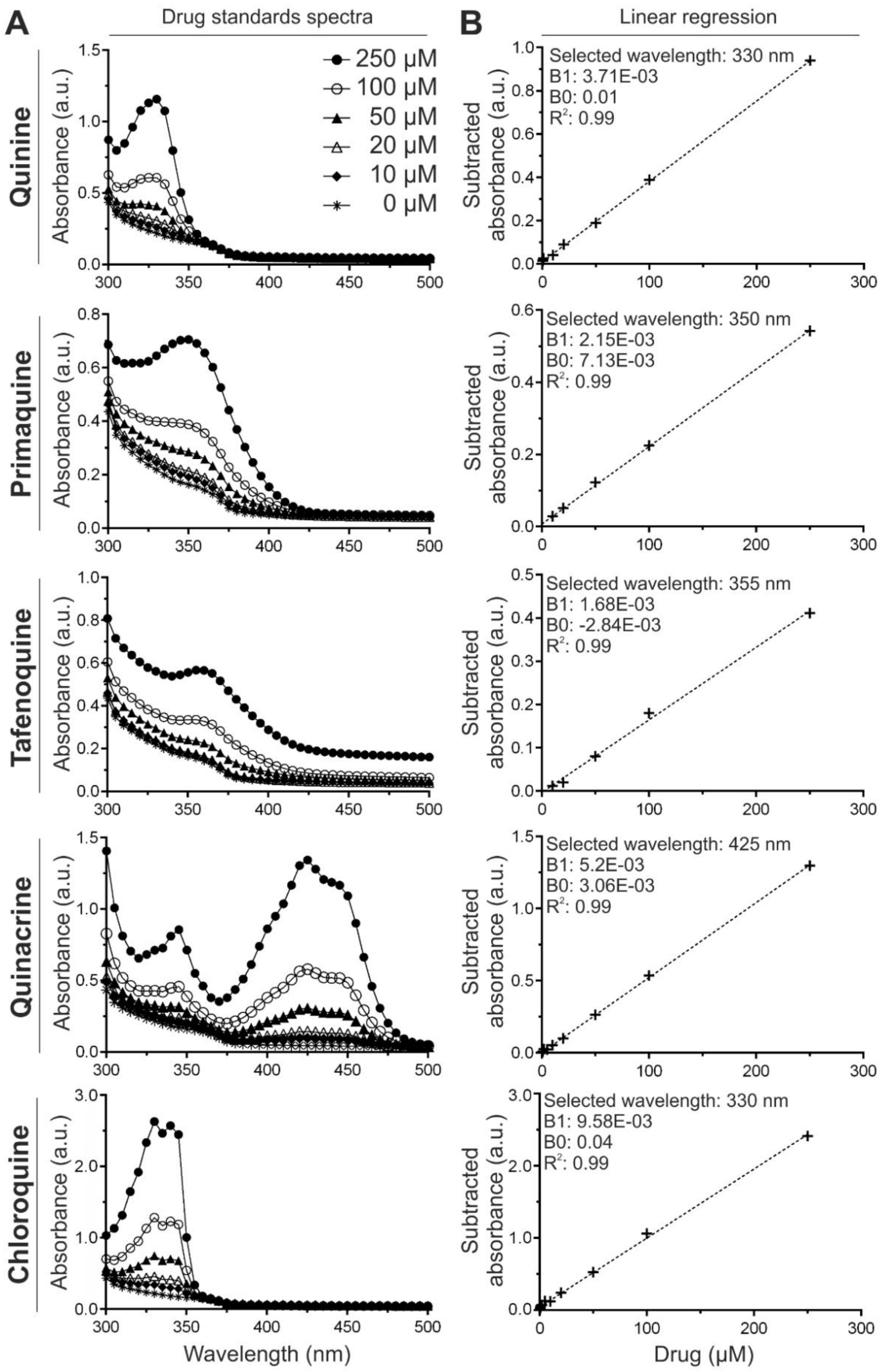

FIGURE S1 | Determination of UV-visible absorption spectra for the quantification of antimalarial drug encapsulation into LPs. (A) 300 to 500-nanometer wavelength range spectra (arbitrary units, a.u.) and (B) linear regression curves obtained from drug standards after blank absorbance subtraction using Epoch™ spectrophotometer in 96-well plate mode.

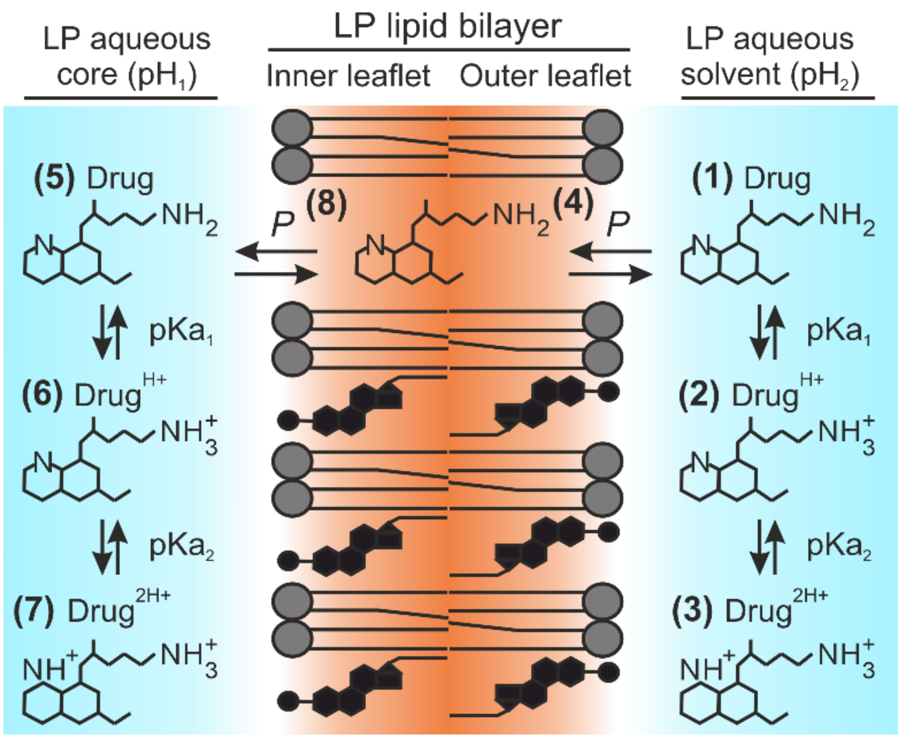

(1) $$mol\ Drug_{AqueousOut} = 1 \times Vol_{AqueousOut}$$

(2) $$mol\ Drug^{H+}_{AqueousOut} = mol\ Drug_{AqueousOut} \times 10^{(pKa_1 - pH_2)}$$

(3) $$mol\ Drug^{2H+}_{AqueousOut} = mol\ Drug^{H+}_{AqueousOut} \times 10^{(pKa_2 - pH_2)}$$

(4) $$mol\ Drug_{OrganicOut} = \frac{P \times Vol_{OrganicOut} \times mol\ Drug_{AqueousOut}}{Vol_{AqueousOut}}$$

(5) $$mol\ Drug_{AqueousIn} = 1 \times Vol_{AqueousIn}$$

(6) $$mol\ Drug^{H+}_{AqueousIn} = mol\ Drug_{AqueousIn} \times 10^{(pKa_1 - pH_1)}$$

(7) $$mol\ Drug^{2H+}_{AqueousIn} = mol\ Drug^{H+}_{AqueousIn} \times 10^{(pKa_2 - pH_1)}$$

(8) $$mol\ Drug_{OrganicIn} = \frac{P \times Vol_{OrganicIn} \times mol\ Drug_{AqueousIn}}{Vol_{AqueousIn}}$$

(9) $$EEt = \frac{(4)+(5)+(6)+(7)+(8)}{(1)+(2)+(3)+(4)+(5)+(6)+(7)+(8)}$$

FIGURE S2 | D_P distribution model. The illustration represents all volumetric fractions and theorized drug microspecies distribution in the experimental liposomal system. Equations reporting drug microspecies molecular abundance in all volumetric fractions and respective drug theoretical EE (EEt) are included.

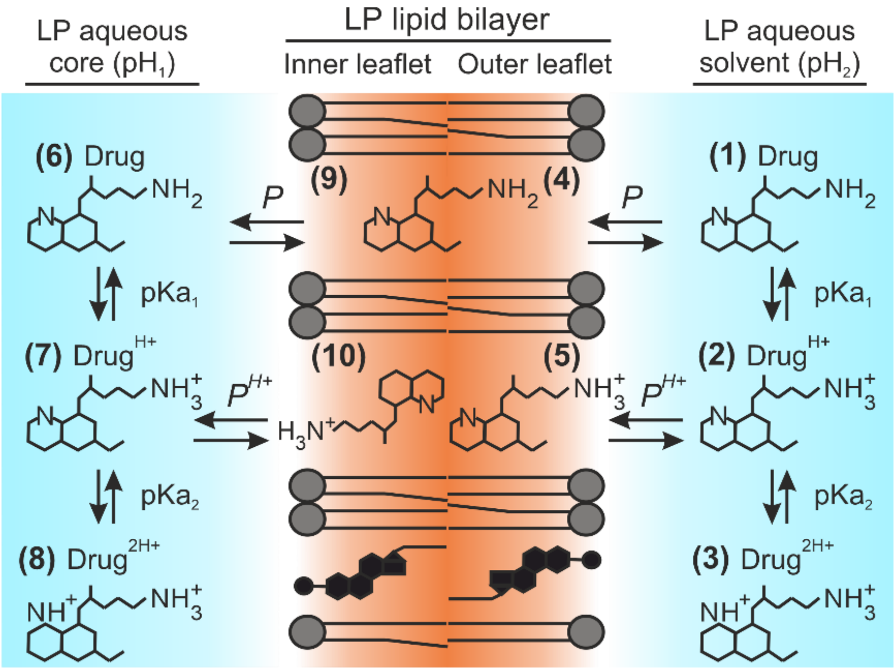

$$(1)\quad mol\ Drug_{AqueousOut} = 1 \times Vol_{AqueousOut}$$

$$(2)\quad mol\ Drug^{H+}_{AqueousOut} = mol\ Drug_{AqueousOut} \times 10^{(pKa_1 - pH_2)}$$

$$(3)\quad mol\ Drug^{2H+}_{AqueousOut} = mol\ Drug^{H+}_{AqueousOut} \times 10^{(pKa_2 - pH_2)}$$

$$(4)\quad mol\ Drug_{OrganicOut} = \frac{P \times Vol_{OrganicOut} \times mol\ Drug_{AqueousOut}}{Vol_{AqueousOut}}$$

$$(5)\quad mol\ Drug^{H+}_{OrganicOut} = \frac{P^{H+} \times Vol_{OrganicOut} \times mol\ Drug^{H+}_{AqueousOut}}{Vol_{AqueousOut}}$$

$$(6)\quad mol\ Drug_{AqueousIn} = 1 \times Vol_{AqueousIn}$$

$$(7)\quad mol\ Drug^{H+}_{AqueousIn} = mol\ Drug_{AqueousIn} \times 10^{(pKa_1 - pH_1)}$$

$$(8)\quad mol\ Drug^{2H+}_{AqueousIn} = mol\ Drug^{H+}_{AqueousIn} \times 10^{(pKa_2 - pH_1)}$$

$$(9)\quad mol\ Drug_{OrganicIn} = \frac{P \times Vol_{OrganicIn} \times mol\ Drug_{AqueousIn}}{Vol_{AqueousIn}}$$

$$(10)\quad mol\ Drug^{H+}_{OrganicIn} = \frac{P^{H+} \times Vol_{OrganicIn} \times mol\ Drug^{H+}_{AqueousIn}}{Vol_{AqueousIn}}$$

$$(11)\quad EEt = \frac{(4)+(5)+(6)+(7)+(8)+(9)+(10)}{(1)+(2)+(3)+(4)+(5)+(6)+(7)+(8)+(9)+(10)}$$

FIGURE S3 | $D_{P,PH+}$ distribution model. The illustration represents all volumetric fractions and theorized drug microspecies distribution in the experimental liposomal system. Equations reporting drug microspecies molecular abundance in all volumetric fractions and respective drug theoretical EE (EEt) are included.

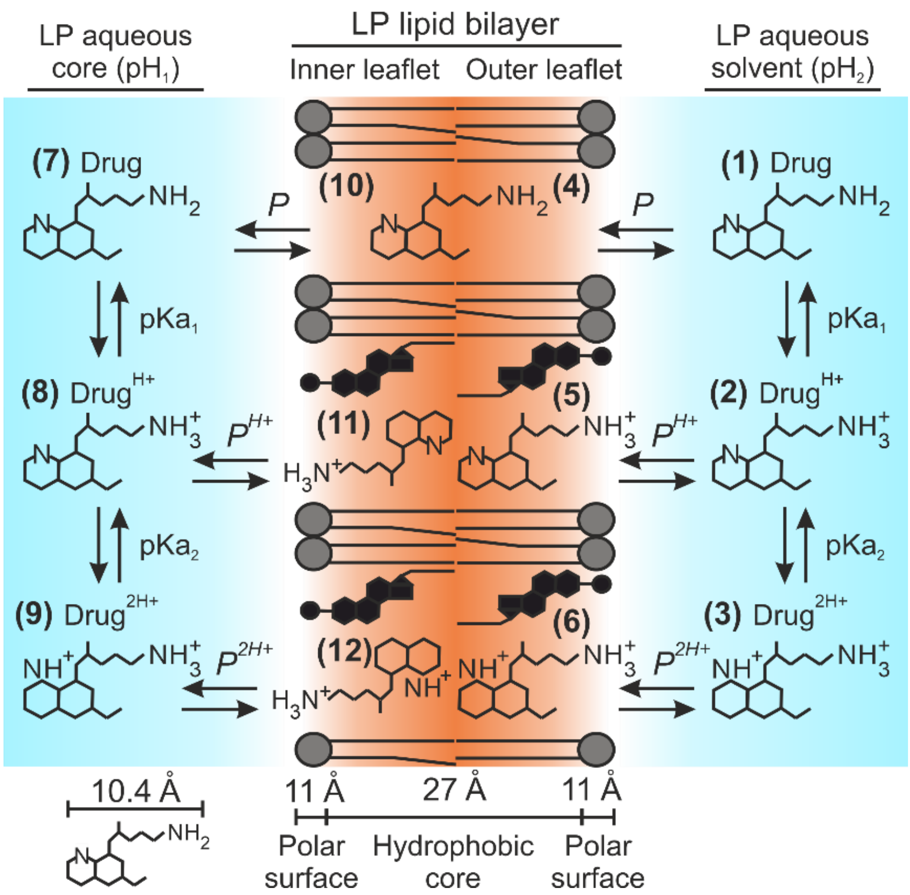

(1) $$mol\ Drug_{AqueousOut} = 1 \times Vol_{AqueousOut}$$

(2) $$mol\ Drug^{H+}_{AqueousOut} = mol\ Drug_{AqueousOut} \times 10^{(pKa_1 - pH_2)}$$

(3) $$mol\ Drug^{2H+}_{AqueousOut} = mol\ Drug^{H+}_{AqueousOut} \times 10^{(pKa_2 - pH_2)}$$

(4) $$mol\ Drug_{OrganicOut} = \frac{P \times Vol_{OrganicOut} \times mol\ Drug_{AqueousOut}}{Vol_{AqueousOut}}$$

(5) $$mol\ Drug^{H+}_{OrganicOut} = \frac{P^{H+} \times Vol_{OrganicOut} \times mol\ Drug^{H+}_{AqueousOut}}{Vol_{AqueousOut}}$$

(6) $$mol\ Drug^{2H+}_{OrganicOut} = \frac{P^{2H+} \times Vol_{OrganicOut} \times mol\ Drug^{2H+}_{AqueousOut}}{Vol_{AqueousOut}}$$

(7) $$mol\ Drug_{AqueousIn} = 1 \times Vol_{AqueousIn}$$

(8) $$mol\ Drug^{H+}_{AqueousIn} = mol\ Drug_{AqueousIn} \times 10^{(pKa_1 - pH_1)}$$

(9) $$mol\ Drug^{2H+}_{AqueousIn} = mol\ Drug^{H+}_{AqueousIn} \times 10^{(pKa_2 - pH_1)}$$

(10) $$mol\ Drug_{OrganicIn} = \frac{P \times Vol_{OrganicIn} \times mol\ Drug_{AqueousIn}}{Vol_{AqueousIn}}$$

(11) $$mol\ Drug^{H+}_{OrganicIn} = \frac{P^{H+} \times Vol_{OrganicIn} \times mol\ Drug^{H+}_{AqueousIn}}{Vol_{AqueousIn}}$$

(12) $$mol\ Drug^{2H+}_{OrganicIn} = \frac{P^{2H+} \times Vol_{OrganicIn} \times mol\ Drug^{2H+}_{AqueousIn}}{Vol_{AqueousIn}}$$

(13) $$EEt = \frac{(4)+(5)+(6)+(7)+(8)+(9)+(10)+(11)+(12)}{(1)+(2)+(3)+(4)+(5)+(6)+(7)+(8)+(9)+(10)+(11)+(12)}$$

FIGURE S4 | $D_{P,PH+,P2H+}$ distribution model. The illustration represents all volumetric fractions and theorized drug microspecies distribution in the experimental liposomal system. Equations reporting drug microspecies molecular abundance in all volumetric fractions and respective drug theoretical EE (EEt) are included.

2. Supplementary Tables

TABLE S1 | LP size and ζ-potential in the presence of antimalarials at pH 7.4 (1:40 drug to lipid ratio). Data are reported as mean ± standard deviation of at least three independent replicates.

Drug	LP formulation	Particle diameter (nm)	Particle ζ-potential (mV)
LP vector	PC	198.0 ± 4.0	−6.49 ± 4.7
	PC:PS	149.3 ± 1.6	−61 ± 1.2
	PC:PS:PE	161.3 ± 2.2	−67.5 ± 3.0
	PC:PS:PE:PEG	153.7 ± 10.8	−25.8 ± 0.7
Quinine	PC	184.2 ± 3.3	−6.8 ± 0.3
	PC:PS	141.6 ± 3.7	−61.5 ± 3.5
	PC:PS:PE	156 ± 4.7	−63.8 ± 4.0
	PC:PS:PE:PEG	155.5 ± 2.5	−25.5 ± 1.8
Primaquine	PC	175.15± 9.3	−7.0 ± 0.2
	PC:PS	139.25 ± 3.9	−61.1 ± 2.7
	PC:PS:PE	143.1 ± 3.2	−63.3 ± 2.6
	PC:PS:PE:PEG	162.5 ± 3.4	−26.9 ± 1.6
Tafenoquine	PC	190.8 ± 10.9	−6.7 ± 0.4
	PC:PS	140.5 ± 4.6	−60.3 ± 2.7
	PC:PS:PE	185.3 ± 3.3	−62.5 ± 3.1
	PC:PS:PE:PEG	138.1 ± 2.3	−26.2 ± 1.4
Quinacrine	PC	165.7 ± 3.4	−5.9 ± 0.2
	PC:PS	135 ± 4.3	−59.4 ± 2.3
	PC:PS:PE	152.5 ± 1.6	−63.4 ± 2.8
	PC:PS:PE:PEG	145.8 ± 3.6	−25.4 ± 2.2
Chloroquine	PC	177.4 ± 1.7	−6.0 ± 0.8
	PC:PS	155.5 ± 1.3	−63.0 ± 2.7
	PC:PS:PE	169.6 ± 0.8	−64.9 ± 2.5
	PC:PS:PE:PEG	158.6 ± 3.9	−24.8 ± 1.5

TABLE S2 | Percentual increase (+) or decrease (−) in drug experimental EE as a function of solution pH and lipid composition. PC-LP composition was used when studying pH-dependent variations in EE (non-highlighted lines). Physiological pH 7.4 was used as physiological condition to retrieve EE as a function of phospholipid composition (grey-highlighted lines). The values reported for each condition pair indicate EE variation (ΔEE, %) taking as reference condition those in the left column. The significance of differences between the studied conditions is expressed as t-test *p* values. Experimental EE was determined as detailed in Supplementary Materials and Methods.

		ΔEE (%)/***p* value**		
Quinine		pH 6.0	pH 7.4	pH 9.0
	pH 4.0	+19.6/**0.16**	+62.3/**4.72×10^{-3}**	+86.4/**3.99×10^{-3}**
	pH 6.0	-	+35.7/**9.78×10^{-3}**	+55.8/**3.62×10^{-3}**
	pH 7.4	-	-	+14.8/**0.03**
		PC:PS	PC:PS:PE	PC:PS:PE:PEG
	PC	+31.3/**6.25×10^{-4}**	+8.5/**0.06**	+6.3/**0.14**
	PC:PS	-	−17.4/**1.31×10^{-3}**	−19.1/**2.03×10^{-3}**
	PC:PS:PE	-	-	−2.1/**0.32**
Primaquine		pH 6.0	pH 7.4	pH 9.0
	pH 4.0	+536.8/**1.51×10^{-3}**	+447.4/**9.47×10^{-4}**	+483.2/**9.95×10^{-4}**
	pH 6.0	-	−14.0/**0.09**	−8.4/**0.20**
	pH 7.4	-	-	+6.5/**0.25**
		PC:PS	PC:PS:PE	PC:PS:PE:PEG
	PC	+68.9/**9.04×10^{-4}**	+52.0/**3.37×10^{-3}**	+55.5/**2.29×10^{-3}**
	PC:PS	-	−10.1/**6.42×10^{-3}**	−8.0/**4.54×10^{-3}**
	PC:PS:PE	-	-	+2.3/**0.24**
Tafenoquine		pH 6.0	pH 7.4	pH 9.0
	pH 4.0	+32.6/**0.12**	+36.9/**0.04**	+59.4/**0.01**
	pH 6.0	-	+3.2/**0.41**	+20.3/**0.11**
	pH 7.4	-	-	+16.5/**0.08**
		PC:PS	PC:PS:PE	PC:PS:PE:PEG
	PC	+8.1/**0.02**	+12.1/**0.01**	+10.6/**0.02**
	PC:PS	-	+3.7/**0.19**	+2.3/**0.30**
	PC:PS:PE	-	-	−1.3/**0.39**
Quinacrine		pH 6.0	pH 7.4	pH 9.0
	pH 4.0	+99.1/**0.06**	+461.9/**1.69×10^{-4}**	+738.9/**2.32×10^{-5}**
	pH 6.0	-	+182.2/**3.94×10^{-4}**	+321.3/**3.68×10^{-5}**
	pH 7.4	-	-	+49.3/**1.25×10^{-4}**
		PC:PS	PC:PS:PE	PC:PS:PE:PEG
	PC	+38.0/**4.49×10^{-4}**	+15.5/**0.02**	+25.0/**3.62×10^{-3}**
	PC:PS	-	−16.3/**1.43×10^{-3}**	−9.4/**4.66×10^{-3}**
	PC:PS:PE	-	-	+8.2/**0.05**
Chloroquine		pH 6.0	pH 7.4	pH 9.0
	pH 4.0	+157.6/**0.02**	+390.2/**0.01**	+727.2/**5.56×10^{-3}**
	pH 6.0	-	+90.3/**0.04**	+221.1/**0.01**
	pH 7.4	-	-	+68.7/**0.04**
		PC:PS	PC:PS:PE	PC:PS:PE:PEG
	PC	+45.2/**5.90×10^{-3}**	+4.6/**0.34**	+23.2/**0.05**
	PC:PS	-	−27.9/**4.35×10^{-3}**	−15.2/**0.03**
	PC:PS:PE	-	-	+17.8/**0.06**

TABLE S3 | Drug distribution example according to the $D_{P,PH+}$ model for a PC-LPs suspension at pH 7.4. Chloroquine (CQ) is used as antimalarial drug of reference. The abundance of CQ microspecies (CQ, CQ^{H+}, CQ^{2H+}) has been calculated considering a single unionized molecule present in the LP external solution (aqueous solvent). CQ partition coefficients (P and P^{H+}) and $pKa_{1,2}$ values used for calculation are summarized in Table 3.

	LPs aqueous core	LPs inner bilayer leaflet	LPs outer bilayer leaflet	Aqueous solvent
Volume (%)	2.40	0.41	0.48	96.71
pH	7.4	-	-	7.4
CQ molecules	2.48×10^{-2}	2.22×10^{2}	2.60×10^{2}	1
CQ^{H+} molecules	15.66	2.12×10^{3}	2.49×10^{3}	6.31×10^{2}
CQ^{2H+} molecules	1.57×10^{2}	-	-	6.31×10^{3}

Drug fractions	Molecules	***% of total molecules***
Total CQ	1.22×10^{4}	***100***
LP-retained CQ	5.26×10^{3}	***43.11***
Free CQ	6.94×10^{3}	***56.89***

TABLE S4 | Drug distribution example according to the $D_{P,PH+,P2H+}$ model for a PC-LPs suspension at pH 7.4. Chloroquine (CQ) is used as antimalarial drug of reference. The abundance of CQ microspecies (CQ, CQ^{H+}, CQ^{2H+}) has been calculated considering a single unionized molecule present in the LP external solution (aqueous solvent). CQ partition coefficients (P, P^{H+} and P^{2H+}) and $pKa_{1,2}$ values used for calculation are summarized in Table 3.

	LPs aqueous core	LPs inner bilayer leaflet	LPs outer bilayer leaflet	Aqueous solvent
Volume (%)	2.40	0.41	0.48	96.71
pH	7.4	-	-	7.4
CQ molecules	2.48×10^{-2}	2.22×10^{2}	2.60×10^{2}	1
CQ^{H+} molecules	15.66	2.12×10^{3}	2.49×10^{3}	6.31×10^{2}
CQ^{2H+} molecules	1.57×10^{2}	4.23×10^{2}	4.96×10^{2}	6.31×10^{3}

Drug fractions	Molecules	***% of total molecules***
Total CQ	1.31×10^{4}	***100***
LP-retained CQ	6.18×10^{3}	***47.10***
Free CQ	6.94×10^{3}	***52.90***

TABLE S5 | Drug distribution example according to the $D_{P,PH+,P2H+}$ model for a PC-LPs suspension holding a pH 4.0-7.4 transmembrane gradient. Chloroquine (CQ) is used as antimalarial drug of reference. The abundance of CQ microspecies (CQ, CQ^{H+}, CQ^{2H+}) has been calculated considering a single unionized molecule present in the LP external solution (aqueous solvent, pH 7.4). CQ partition coefficients (P, P^{H+} and P^{2H+}) and $pKa_{1,2}$ values used for calculation are summarized in Table 3.

	LPs aqueous core	LPs inner bilayer leaflet	LPs outer bilayer leaflet	Aqueous solvent
Volume (%)	2.40	0.41	0.48	96.71
pH	4.0	-	-	7.4
CQ molecules	2.48×10^{-2}	2.22×10^{2}	2.60×10^{2}	1
CQ^{H+} molecules	3.93×10^{4}	5.32×10^{6}	2.49×10^{3}	6.31×10^{2}
CQ^{2H+} molecules	9.88×10^{8}	2.67×10^{9}	4.96×10^{2}	6.31×10^{3}

Drug fractions	Molecules	***% of total molecules***
Total CQ	3.66×10^{9}	***100***
LP-retained CQ	3.66×10^{9}	***100.00***
Aqueous LP core (all CQ species)	9.88×10^{8}	***26.99***
Inner leaflet (CQ^{H+})	5.32×10^{6}	***0.15***
Inner leaflet (CQ^{2H+})	2.67×10^{9}	***72.87***
Lipid bilayer (CQ)	4.82×10^{2}	***1.32×10^{-5}***
Outer leaflet (CQ^{H+})	2.49×10^{3}	***6.79×10^{-5}***
Outer leaflet (CQ^{2H+})	4.96×10^{2}	***1.36×10^{-5}***
Free CQ	6.94×10^{3}	***1.90×10^{-4}***

TABLE S6 | Drug distribution example according to the $D_{P,PH+}$ model for a PC/PS vesicular system simulating a RBCs suspension. Chloroquine (CQ) is used as antimalarial drug of reference. The abundance of CQ microspecies (CQ, CQ^{H+}, CQ^{2H+}) has been calculated considering a single unionized molecule present in the RBC external solution (pH 7.4). CQ partition coefficients (P and P^{H+}) and $pKa_{1,2}$ values used for calculation are summarized in Table 3. [a]PC:PS (70:30%)- and [b]PC(100%)-based lipid formulations were considered replicating RBC inner and outer plasma membrane leaflets. [c]Total unionized CQ molecules distributed throughout inner and outer membrane leaflets

	RBCs cytoplasm	RBCs inner membrane leaflet[a]	RBCs outer membrane leaflet[b]	External solution
Volume (%)	39.80	0.10	0.10	60.00
pH	7.4	-	-	7.4
CQ molecules	0.66	86.60	86.60	1
CQ^{H+} molecules	4.19×10^{2}	1.65×10^{3}	8.27×10^{2}	6.31×10^{2}
CQ^{2H+} molecules	4.19×10^{3}	-	-	6.31×10^{3}

Drug fractions	Molecules	***% of total molecules***
Total CQ	1.42×10^{4}	***100.00***
RBC-retained CQ	7.26×10^{3}	***51.10***
RBC cytoplasm (all CQ species)	4.60×10^{3}	***32.44***
Inner leaflet[a] (CQ^{H+})	1.65×10^{3}	***11.62***
Membrane[c] (CQ)	1.73×10^{2}	***1.22***
Outer leaflet[b] (CQ^{H+})	8.27×10^{2}	***5.83***
Free CQ	6.94×10^{3}	***48.90***

3. Supplementary Materials and Methods

Quantification of drug experimental Encapsulation Efficiency in LP suspensions

Drug encapsulated amounts in LPs were determined after 24 h of incubation (1:40 drug to lipid mole ratio, *i.e.* 0.25 mM drug for 10 mM lipid). Briefly, LPs were pelleted by ultracentrifugation (150,000 *g*, 4 °C, 1 h) and treated with 1% sodium dodecyl sulphate coupled to 60 °C bath sonication. Drug extracts were analysed by UV-visible spectroscopy using an Epoch^{TM} spectrophotometer (BioTek Instruments, Inc., Winooski, VT, USA) in 96-well plate mode. Drug standards for quantification were prepared in 1% sodium dodecyl sulphate and the same solvent was used as blank control for absorbance subtraction. Standard curves were obtained by linear regression from at least three independent measurements (Figure S1). Unencapsulated drug amounts were determined by UV-visible spectroscopy from LP supernatants. Drug Encapsulation Efficiency (EE) was finally determined as the percentual amount of drug retained in LPs relative to the total amount present in the sample (LPs + external solution).

Supplementary Material

Appendix

Mathematical algorithms used in this work
(Wolfram Mathematica code)

1. Analysis of theoretical Encapsulation Efficiency (EEt) fitting to experimental data (EEe) for a PC-LPs suspension at pH 4.0-9.0 range. EEt data estimated in accordance with the DP distribution model (Figure 1). Calculations performed using Chloroquine as diprotic drug example.

a) Definition of the directory and import of pH-dependent experimental Encapsulation Efficiency (EEe) data in PC-LPs (pH 4.0-9.0)

```
SetDirectory["C:\\Mathematica export"];
pHEncValuesRaw = Import["pH dependent partitioning.txt", "Table"]
pHEncValues = Transpose[Delete[Transpose[Delete[pHEncValuesRaw, 1]], 1]];
```

```
{{Drug, pH, 4., pH, 6., pH, 7.4, pH, 9.}, {Quinine, 38.17, 45.66, 62.04, 71.18},
 {Chloroquine, 9.23, 23.69, 45.06, 76.06}, {Primaquine, 9.48, 60.48, 52.02, 55.39},
 {Tafenoquine, 54., 71.57, 73.9, 86.05}, {Quinacrine, 11.35, 22.48, 63.52, 94.8}}
```

b) Definition of parameters for a PC-LPs suspension at 10 mM lipid concentration, 100 nm particles. Chloroquine P coefficient is reported in Table 3. All volumetric fractions comprised within our liposomal system include : (i) LPs aqueous core (VolAqueousIn), (ii) LPs lipid bilayer inner (VolOrganicIn) and outer (VolOrganicOut) leaflets, and (iii) LPs aqueous solvent (VolAqueousOut)

```
pKa1 = 10.2;
pKa2 = 8.4;
logP = 4.72;
DilutionSample = 1;
VolAqueousIn = 2.4 / DilutionSample;
VolOrganicOut = 0.48 / DilutionSample;
VolOrganicIn = 0.408888 / DilutionSample;
VolAqueousOut = 100 - VolAqueousIn - VolOrganicOut - VolOrganicIn;
pH = {4, 6, 7.4, 9};
```

Printed by Wolfram Mathematica Student Edition

c) Determination of the molecular abundance for all drug microspecies present in the liposomal system, together with respective EEt values

```
FunctionEncap[i_] := Module[{Encapsulated, Name,
    MolesAqueousOut, MolesAqueousIn, MolesOrganicOut, MolesOrganicIn},
   MolesAqueousOut =
    (1 * VolAqueousOut) + (1 * VolAqueousOut * 10^ (pKa1 - pH[[i]])) +
     (1 * VolAqueousOut * 10^ (pKa1 + pKa2 - (2 * pH[[i]])));
   MolesAqueousIn =
    (1 * VolAqueousIn) + (1 * VolAqueousIn * 10^ (pKa1 - pH[[i]])) +
     (1 * VolAqueousIn * 10^ (pKa1 + pKa2 - (2 * pH[[i]])));
   MolesOrganicOut =
    (1 * VolAqueousOut * (10^logP) * (VolOrganicOut / VolAqueousOut));
   MolesOrganicIn =
    (1 * VolAqueousIn * (10^logP) * (VolOrganicIn / VolAqueousIn));
   Encapsulated =
    (MolesAqueousIn + MolesOrganicOut + MolesOrganicIn) *
     100 / (MolesAqueousOut + MolesAqueousIn + MolesOrganicOut + MolesOrganicIn);
   Encapsulated
  ]
WorkFunctionEncap = Table[FunctionEncap[i], {i, 1, Dimensions[pH][[1]]}];
Export["Chloroquine_DPModel.dat",
   Insert[{WorkFunctionEncap}, {"pH 4", "pH 6", "pH 7.4", "pH 9"}, 1]];
Insert[{WorkFunctionEncap}, {"pH 4", "pH 6", "pH 7.4", "pH 9"}, 1]
{{pH 4, pH 6, pH 7.4, pH 9}, {2.42153, 2.43301, 8.6178, 95.8647}}
```

2. Theoretical estimation of PH+ coefficient for a PC-LPs suspension at pH 4.0-9.0 range. Calculations performed based on theoretical Encapsulation Efficiency (EEt) fitting to experimental data (EEe). EEt data estimated in accordance with the DP,PH+ distribution model (Figure 4). Chloroquine is used here as diprotic drug example.

a) Definition of the directory and import of pH-dependent experimental Encapsulation Efficiency (EEe) data in PC-LPs (pH 4.0-9.0)

```
SetDirectory["C:\\Mathematica export"];
pHEncValuesRaw = Import["pH dependent partitioning.txt", "Table"]
pHEncValues = Transpose[Delete[Transpose[Delete[pHEncValuesRaw, 1]], 1]];
{{Drug, pH, 4., pH, 6., pH, 7.4, pH, 9.}, {Quinine, 38.17, 45.66, 62.04, 71.18},
 {Chloroquine, 9.23, 23.69, 45.06, 76.06}, {Primaquine, 9.48, 60.48, 52.02, 55.39},
 {Tafenoquine, 54., 71.57, 73.9, 86.05}, {Quinacrine, 11.35, 22.48, 63.52, 94.8}}
```

b) Definition of parameters for a PC-LPs suspension at 10 mM lipid

concentration, 100 nm particles. Chloroquine P coefficient is reported in Table 3. All volumetric fractions comprised within our liposomal system include : (i) LPs aqueous core (VolAqueousIn), (ii) LPs lipid bilayer inner (VolOrganicIn) and outer (VolOrganicOut) leaflets, and (iii) LPs aqueous solvent (VolAqueousOut)

```
pKa1 = 10.2;
pKa2 = 8.4;
logP = 4.72;
DilutionSample = 1;
VolAqueousIn = 2.4 / DilutionSample;
VolOrganicOut = 0.48 / DilutionSample;
VolOrganicIn = 0.408888 / DilutionSample;
VolAqueousOut = 100 - VolAqueousIn - VolOrganicOut - VolOrganicIn;
pH = {4, 6, 7.4, 9};
```

c) Determination of the molecular abundance for all drug microspecies present in the liposomal system as a function of hypothetical PH+ values, 1 to 6 log10 units (0.1 interval), together with respective EEt values

```
FunctionEncap[i_, logPH_] := Module[{Encapsulated, Name,
   MolesAqueousOut, MolesAqueousIn, MolesOrganicOut, MolesOrganicIn},
  MolesAqueousOut =
   (1 * VolAqueousOut) + (1 * VolAqueousOut * 10^ (pKa1 - pH[[i]])) +
    (1 * VolAqueousOut * 10^ (pKa1 + pKa2 - (2 * pH[[i]])));
  MolesAqueousIn =
   (1 * VolAqueousIn) + (1 * VolAqueousIn * 10^ (pKa1 - pH[[i]])) +
    (1 * VolAqueousIn * 10^ (pKa1 + pKa2 - (2 * pH[[i]])));
  MolesOrganicOut =
   (1 * VolAqueousOut * (10^logP) * (VolOrganicOut / VolAqueousOut)) +
    ((1 * VolAqueousOut * 10^ (pKa1 - pH[[i]])) *
      (10^logPH) * (VolOrganicOut / VolAqueousOut));
  MolesOrganicIn =
   (1 * VolAqueousIn * (10^logP) * (VolOrganicIn / VolAqueousIn)) +
    ((1 * VolAqueousIn * 10^ (pKa1 - pH[[i]])) *
      (10^logPH) * (VolOrganicIn / VolAqueousIn));
  Encapsulated =
   (MolesAqueousIn + MolesOrganicOut + MolesOrganicIn) *
    100 / (MolesAqueousOut + MolesAqueousIn + MolesOrganicOut + MolesOrganicIn);
  Encapsulated
 ]

WorkFunctionEncap =
  Table[FunctionEncap[i, logPH], {i, 1, Dimensions[pH][[1]]}, {logPH, 1., 6., 0.1}];
Export["Chloroquine_DP,PH+Model.dat", Transpose[
   Insert[Transpose[WorkFunctionEncap], {"pH 4", "pH 6", "pH 7.4", "pH 9"}, 1]]];
```

```
Transpose[Insert[Transpose[WorkFunctionEncap], {"pH 4", "pH 6", "pH 7.4", "pH 9"}, 1]]
```

```
{{pH 4, 2.42187, 2.42196, 2.42208, 2.42222, 2.4224, 2.42263, 2.42291, 2.42327,
  2.42372, 2.42429, 2.42501, 2.42591, 2.42705, 2.42848, 2.43028, 2.43254,
  2.43539, 2.43898, 2.4435, 2.44919, 2.45635, 2.46537, 2.47671, 2.49099, 2.50896,
  2.53157, 2.56002, 2.59582, 2.64085, 2.69748, 2.76867, 2.85816, 2.97057,
  3.11173, 3.28885, 3.51092, 3.78904, 4.1369, 4.57127, 5.11254, 5.78529, 6.6188,
  7.64737, 8.9105, 10.4524, 12.3208, 14.565, 17.232, 20.3618, 23.9807, 28.0942},
 {pH 6, 2.4677, 2.47667, 2.48797, 2.50219, 2.52008, 2.5426, 2.57094, 2.60659,
  2.65143, 2.70782, 2.77872, 2.86784, 2.97979, 3.12037, 3.29677, 3.51793,
  3.79492, 4.14137, 4.574, 5.11313, 5.78325, 6.61355, 7.63825, 8.89672, 10.4331,
  12.2952, 14.5321, 17.191, 20.3119, 23.9217, 28.0261, 32.6036, 37.5999, 42.9264,
  48.4645, 54.0747, 59.61, 64.9313, 69.9203, 74.4892, 78.5844, 82.1847, 85.2967,
  87.9471, 90.1765, 92.0319, 93.5626, 94.8162, 95.8369, 96.6639, 97.3312},
 {pH 7.4, 9.31017, 9.48773, 9.71029, 9.98892, 10.3373, 10.772, 11.3133, 11.9855,
  12.8174, 13.8426, 15.0995, 16.6306, 18.4814, 20.6977, 23.3222, 26.3891,
  29.918, 33.9069, 38.3261, 43.1146, 48.1797, 53.403, 58.6502, 63.7842, 68.6799,
  73.2348, 77.3769, 81.0657, 84.2905, 87.0641, 89.4165, 91.3881, 93.0241,
  94.3705, 95.4709, 96.3654, 97.0891, 97.6726, 98.1415, 98.5175, 98.8185, 99.059,
  99.251, 99.404, 99.5259, 99.623, 99.7003, 99.7618, 99.8107, 99.8496, 99.8805},
 {pH 9, 95.8766, 95.8797, 95.8835, 95.8884, 95.8945, 95.9021, 95.9117, 95.9237,
  95.9388, 95.9575, 95.9809, 96.0099, 96.0459, 96.0903, 96.1447, 96.2112, 96.2916,
  96.3882, 96.5028, 96.6372, 96.7923, 96.9684, 97.1644, 97.3778, 97.6047, 97.84,
  98.0778, 98.3117, 98.536, 98.7458, 98.9375, 99.1089, 99.2594, 99.3892, 99.4996,
  99.5924, 99.6695, 99.7331, 99.7851, 99.8275, 99.8618, 99.8895, 99.9117,
  99.9296, 99.9438, 99.9553, 99.9644, 99.9717, 99.9775, 99.9821, 99.9857}}
```

d) Calculation of EEe vs. EEt variance as a function of PH+ value

```
logPHlist = Table[i, {i, 1, 6, 0.1}]
DrugEncValues = pHEncValues[[2]]
```

```
{1., 1.1, 1.2, 1.3, 1.4, 1.5, 1.6, 1.7, 1.8, 1.9, 2., 2.1, 2.2, 2.3, 2.4, 2.5, 2.6,
 2.7, 2.8, 2.9, 3., 3.1, 3.2, 3.3, 3.4, 3.5, 3.6, 3.7, 3.8, 3.9, 4., 4.1, 4.2, 4.3,
 4.4, 4.5, 4.6, 4.7, 4.8, 4.9, 5., 5.1, 5.2, 5.3, 5.4, 5.5, 5.6, 5.7, 5.8, 5.9, 6.}
```

```
{9.23, 23.69, 45.06, 76.06}
```

```
FunctionVarianceEnc[i_, j_] := Module[{EncapVar},
  EncapVar = (DrugEncValues[[i]] - WorkFunctionEncap[[i, j]])^2
 ]
```

```
TableFunctionVarianceEnc =
 Transpose[Insert[Transpose[Insert[Table[FunctionVarianceEnc[i, j],
      {i, 1, Dimensions[pH][[1]]}, {j, 1, Dimensions[WorkFunctionEncap][[2]]}],
     logPHlist, 1]], Insert[pH, "logPH+", 1], 1]]
Export["ChloroquineVarianceEnc.dat", TableFunctionVarianceEnc];
```

```
{{logPH+, 1., 1.1, 1.2, 1.3, 1.4, 1.5, 1.6, 1.7, 1.8, 1.9, 2., 2.1, 2.2, 2.3, 2.4, 2.5, 2.6,
  2.7, 2.8, 2.9, 3., 3.1, 3.2, 3.3, 3.4, 3.5, 3.6, 3.7, 3.8, 3.9, 4., 4.1, 4.2, 4.3,
  4.4, 4.5, 4.6, 4.7, 4.8, 4.9, 5., 5.1, 5.2, 5.3, 5.4, 5.5, 5.6, 5.7, 5.8, 5.9, 6.},
 {4, 46.3506, 46.3493, 46.3478, 46.3459, 46.3434, 46.3403, 46.3364, 46.3315,
  46.3254, 46.3176, 46.3079, 46.2956, 46.2802, 46.2607, 46.2362, 46.2054, 46.1667,
  46.1179, 46.0565, 45.9794, 45.8823, 45.7603, 45.6069, 45.4143, 45.1724, 44.869,
  44.4886, 44.0123, 43.4169, 42.6739, 41.7487, 40.6004, 39.1804, 37.4332,
  35.2972, 32.7079, 29.604, 25.9397, 21.7038, 16.9535, 11.866, 6.81839, 2.50471,
  0.102083, 1.4942, 9.55312, 28.4623, 64.0324, 123.917, 217.582, 355.858},
 {6, 450.386, 450.005, 449.526, 448.923, 448.165, 447.212, 446.015, 444.51,
  442.622, 440.252, 437.282, 433.563, 428.913, 423.11, 415.884, 406.913, 395.814,
  382.149, 365.421, 345.1, 320.652, 291.605, 257.659, 218.841, 175.745, 129.842,
  83.8678, 42.2376, 11.4113, 0.0536646, 18.8019, 79.453, 193.484, 370.037,
  613.774, 923.228, 1290.25, 1700.84, 2137.24, 2580.56, 3013.4, 3421.63, 3795.38,
  4128.98, 4420.45, 4670.62, 4882.18, 5058.94, 5205.17, 5325.18, 5423.03},
 {7.4, 1278.05, 1265.39, 1249.6, 1229.98, 1205.67, 1175.67, 1138.84, 1093.92,
  1039.59, 974.526, 897.633, 808.231, 706.424, 593.522, 472.533, 348.602, 229.28,
  124.392, 45.345, 3.78476, 9.73247, 69.6061, 184.692, 350.597, 557.899, 793.821,
  1044.38, 1296.41, 1539.03, 1764.35, 1967.5, 2146.3, 2300.56, 2431.52, 2541.26,
  2632.24, 2707.03, 2768.08, 2817.65, 2857.71, 2889.98, 2915.89, 2936.66,
  2953.27, 2966.54, 2977.13, 2985.56, 2992.29, 2997.64, 3001.9, 3005.28},
 {9, 392.697, 392.819, 392.972, 393.165, 393.406, 393.71, 394.091, 394.568,
  395.165, 395.911, 396.842, 397.999, 399.436, 401.211, 403.396, 406.069, 409.318,
  413.234, 417.908, 423.419, 429.829, 437.161, 445.394, 454.447, 464.173, 474.369,
  484.782, 495.138, 505.171, 514.645, 523.378, 531.252, 538.21, 544.251, 549.415,
  553.773, 557.409, 560.416, 562.882, 564.894, 566.525, 567.843, 568.904,
  569.756, 570.438, 570.984, 571.42, 571.768, 572.045, 572.266, 572.442}}
```

e) Graphical representation of EEe vs. EEt variance as a function of PH+ value

```
SetDirectory["C:\\Mathematica export"];

TableFunctionVarianceEnc = Import["ChloroquineVarianceEnc.dat", "Table"];

CleanTableVarianceEnc =
  Transpose[Delete[Transpose[Delete[TableFunctionVarianceEnc, 1]], 1]];

NormCleanTableVarianceEnc[i_, j_] := Module[{Sorting, NormValue},
  Sorting = Sort[CleanTableVarianceEnc[[i]]];
  NormValue = 100 - ((CleanTableVarianceEnc[[i, j]] - Sorting[[1]]) *
      100 / (Sorting[[-1]] - Sorting[[1]]))
 ]
```

```
NormTableVarianceEnc =
 Table[NormCleanTableVarianceEnc[i, j], {i, 1, Dimensions[CleanTableVarianceEnc][[1]]},
  {j, 1, Dimensions[CleanTableVarianceEnc][[2]]}]
```

```
{{87., 87.0003, 87.0007, 87.0013, 87.002, 87.0028, 87.0039, 87.0053, 87.007,
  87.0092, 87.0119, 87.0154, 87.0197, 87.0252, 87.0321, 87.0407, 87.0516,
  87.0654, 87.0826, 87.1043, 87.1316, 87.1659, 87.209, 87.2631, 87.3311,
  87.4164, 87.5233, 87.6572, 87.8246, 88.0334, 88.2935, 88.6163, 89.0154,
  89.5065, 90.107, 90.8348, 91.7073, 92.7373, 93.928, 95.2632, 96.6933, 98.1121,
  99.3246, 100., 99.6087, 97.3434, 92.0282, 82.0297, 65.1968, 38.8682, 0.},
 {91.6958, 91.7029, 91.7117, 91.7228, 91.7368, 91.7544, 91.7765, 91.8042,
  91.839, 91.8827, 91.9375, 92.0061, 92.0918, 92.1988, 92.3321, 92.4975,
  92.7022, 92.9541, 93.2626, 93.6373, 94.0882, 94.6238, 95.2497, 95.9656,
  96.7602, 97.6067, 98.4545, 99.2221, 99.7906, 100., 99.6543, 98.5359, 96.4331,
  93.1775, 88.683, 82.9766, 76.2087, 68.6374, 60.5902, 52.4153, 44.4338, 36.9059,
  30.014, 23.8624, 18.4876, 13.8746, 9.97336, 6.71384, 4.01729, 1.80433, 0.},
 {57.5457, 57.9676, 58.4935, 59.1472, 59.9572, 60.9567, 62.1837, 63.6802,
  65.4905, 67.6581, 70.2199, 73.1985, 76.5904, 80.3519, 84.3829, 88.5118, 92.4872,
  95.9818, 98.6154, 100., 99.8018, 97.8071, 93.9728, 88.4454, 81.5387, 73.6786,
  65.3308, 56.9339, 48.8506, 41.3439, 34.5754, 28.6186, 23.4791, 19.1157,
  15.4596, 12.4285, 9.93685, 7.90279, 6.25143, 4.91667, 3.84162, 2.9782, 2.28631,
  1.73286, 1.2908, 0.938094, 0.656945, 0.432997, 0.254712, 0.112846, 0.},
 {100., 99.9322, 99.8469, 99.7397, 99.6052, 99.4362, 99.2244, 98.959, 98.6268,
  98.2117, 97.694, 97.0499, 96.2508, 95.2631, 94.0477, 92.5605, 90.7529,
  88.5742, 85.974, 82.9077, 79.3419, 75.2626, 70.6822, 65.6459, 60.2344, 54.5622,
  48.7691, 43.0073, 37.4256, 32.1547, 27.296, 22.9154, 19.0444, 15.6837, 12.8104,
  10.3861, 8.36303, 6.69043, 5.31812, 4.19923, 3.29163, 2.55847, 1.96821,
  1.49429, 1.11458, 0.810906, 0.568366, 0.374872, 0.220641, 0.0977943, 0.}}
```

```
logPHlist = Table[i, {i, 1, 6, 0.1}]
```

```
{1., 1.1, 1.2, 1.3, 1.4, 1.5, 1.6, 1.7, 1.8, 1.9, 2., 2.1, 2.2, 2.3, 2.4, 2.5, 2.6,
 2.7, 2.8, 2.9, 3., 3.1, 3.2, 3.3, 3.4, 3.5, 3.6, 3.7, 3.8, 3.9, 4., 4.1, 4.2, 4.3,
 4.4, 4.5, 4.6, 4.7, 4.8, 4.9, 5., 5.1, 5.2, 5.3, 5.4, 5.5, 5.6, 5.7, 5.8, 5.9, 6.}
```

```
RowTick = {{1, "pH 4.0"}, {2, "pH 6.0"}, {3, "pH 7.4"}, {4, "pH 9.0"}};
ColumnTick = Table[{i, logPHlist[[i]]}, {i, 1, Dimensions[logPHlist][[1]]}];
ClusterG = MatrixPlot[NormTableVarianceEnc, Frame → True,
  ColorFunction → Hue, FrameTicks → {RowTick, ColumnTick, RowTick, ColumnTick}]
```

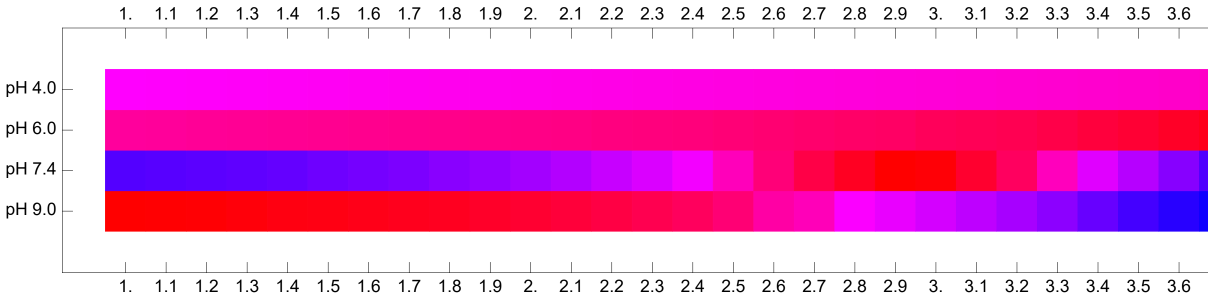

f) Determination of least variant PH+ value across all pH values

```
SetDirectory["C:\\Mathematica export"];
VarpHEncValues = Import["ChloroquineVarianceEnc.dat", "Table"]
```

```
{{logPH+, 1., 1.1, 1.2, 1.3, 1.4, 1.5, 1.6, 1.7, 1.8, 1.9, 2., 2.1, 2.2, 2.3, 2.4, 2.5, 2.6,
  2.7, 2.8, 2.9, 3., 3.1, 3.2, 3.3, 3.4, 3.5, 3.6, 3.7, 3.8, 3.9, 4., 4.1, 4.2, 4.3,
  4.4, 4.5, 4.6, 4.7, 4.8, 4.9, 5., 5.1, 5.2, 5.3, 5.4, 5.5, 5.6, 5.7, 5.8, 5.9, 6.},
 {4, 46.3506, 46.3493, 46.3478, 46.3459, 46.3434, 46.3403, 46.3364, 46.3315,
  46.3254, 46.3176, 46.3079, 46.2956, 46.2802, 46.2607, 46.2362, 46.2054, 46.1667,
  46.1179, 46.0565, 45.9794, 45.8823, 45.7603, 45.6069, 45.4143, 45.1724, 44.869,
  44.4886, 44.0123, 43.4169, 42.6739, 41.7487, 40.6004, 39.1804, 37.4332,
  35.2972, 32.7079, 29.604, 25.9397, 21.7038, 16.9535, 11.866, 6.81839, 2.50471,
  0.102083, 1.4942, 9.55312, 28.4623, 64.0324, 123.917, 217.582, 355.858},
 {6, 450.386, 450.005, 449.526, 448.923, 448.165, 447.212, 446.015, 444.51,
  442.622, 440.252, 437.282, 433.563, 428.913, 423.11, 415.884, 406.913, 395.814,
  382.149, 365.421, 345.1, 320.652, 291.605, 257.659, 218.841, 175.745, 129.842,
  83.8678, 42.2376, 11.4113, 0.0536646, 18.8019, 79.453, 193.484, 370.037,
  613.774, 923.228, 1290.25, 1700.84, 2137.24, 2580.56, 3013.4, 3421.63, 3795.38,
  4128.98, 4420.45, 4670.62, 4882.18, 5058.94, 5205.17, 5325.18, 5423.03},
 {7.4, 1278.05, 1265.39, 1249.6, 1229.98, 1205.67, 1175.67, 1138.84, 1093.92,
  1039.59, 974.526, 897.633, 808.231, 706.424, 593.522, 472.533, 348.602, 229.28,
  124.392, 45.345, 3.78476, 9.73247, 69.6061, 184.692, 350.597, 557.899, 793.821,
  1044.38, 1296.41, 1539.03, 1764.35, 1967.5, 2146.3, 2300.56, 2431.52, 2541.26,
  2632.24, 2707.03, 2768.08, 2817.65, 2857.71, 2889.98, 2915.89, 2936.66,
  2953.27, 2966.54, 2977.13, 2985.56, 2992.29, 2997.64, 3001.9, 3005.28},
 {9, 392.697, 392.819, 392.972, 393.165, 393.406, 393.71, 394.091, 394.568,
  395.165, 395.911, 396.842, 397.999, 399.436, 401.211, 403.396, 406.069, 409.318,
  413.234, 417.908, 423.419, 429.829, 437.161, 445.394, 454.447, 464.173, 474.369,
  484.782, 495.138, 505.171, 514.645, 523.378, 531.252, 538.21, 544.251, 549.415,
  553.773, 557.409, 560.416, 562.882, 564.894, 566.525, 567.843, 568.904,
  569.756, 570.438, 570.984, 571.42, 571.768, 572.045, 572.266, 572.442}}
```

```
Sumatory = Insert[{Table[(VarpHEncValues[[2, j]] +
      VarpHEncValues[[3, j]] + VarpHEncValues[[4, j]] + VarpHEncValues[[5, j]]),
    {j, 2, Dimensions[VarpHEncValues][[2]]}]}, Delete[VarpHEncValues[[1]], 1], 1]
Export["ChloroquineTotalVarlogPH+.dat", Sumatory];
```

```
{{1., 1.1, 1.2, 1.3, 1.4, 1.5, 1.6, 1.7, 1.8, 1.9, 2., 2.1, 2.2, 2.3, 2.4, 2.5, 2.6, 2.7,
  2.8, 2.9, 3., 3.1, 3.2, 3.3, 3.4, 3.5, 3.6, 3.7, 3.8, 3.9, 4., 4.1, 4.2, 4.3, 4.4,
  4.5, 4.6, 4.7, 4.8, 4.9, 5., 5.1, 5.2, 5.3, 5.4, 5.5, 5.6, 5.7, 5.8, 5.9, 6.},
 {2167.48, 2154.56, 2138.45, 2118.41, 2093.58, 2062.93, 2025.28, 1979.33,
  1923.7, 1857.01, 1778.06, 1686.09, 1581.05, 1464.1, 1338.05, 1207.79, 1080.58,
  965.893, 874.731, 818.283, 806.095, 844.132, 933.352, 1069.3, 1242.99, 1442.9,
  1657.52, 1877.8, 2099.03, 2321.72, 2551.43, 2797.6, 3071.43, 3383.25, 3739.75,
  4141.95, 4584.29, 5055.28, 5539.47, 6020.12, 6481.76, 6912.19, 7303.45,
  7652.11, 7958.92, 8228.28, 8467.63, 8687.03, 8898.77, 9116.93, 9356.61}}
```

```
LogPH = Sumatory[[1, Ordering[Sumatory[[2]]][[1]]]]
```

```
3.
```

3. Theoretical estimation of P2H+ coefficient for a PC-LPs

suspension at pH 4.0-9.0. Calculations performed based on theoretical Encapsulation Efficiency (EEt) fitting to experimental data (EEe). EEt data estimated in accordance with the DP,PH+,P2H+ distribution model (Figure 6). Chloroquine is used here as diprotic drug example.

a) Definition of the directory and import of pH-dependent experimental Encapsulation Efficiency (EEe) data in PC-LPs (pH 4.0-9.0)

```
SetDirectory["C:\\Mathematica export"];
pHEncValuesRaw = Import["pH dependent partitioning.txt", "Table"]
pHEncValues = Transpose[Delete[Transpose[Delete[pHEncValuesRaw, 1]], 1]];
```

```
{{Drug, pH, 4., pH, 6., pH, 7.4, pH, 9.}, {Quinine, 38.17, 45.66, 62.04, 71.18},
 {Chloroquine, 9.23, 23.69, 45.06, 76.06}, {Primaquine, 9.48, 60.48, 52.02, 55.39},
 {Tafenoquine, 54., 71.57, 73.9, 86.05}, {Quinacrine, 11.35, 22.48, 63.52, 94.8}}
```

b) Definition of parameters for a PC-LPs suspension at 10 mM lipid concentration, 100 nm particles. Chloroquine P and PH+ coefficients are reported in Table 3. All volumetric fractions comprised within our liposomal system include : (i) LPs aqueous core (VolAqueousIn), (ii) LPs lipid bilayer inner (VolOrganicIn) and outer (VolOrganicOut) leaflets, and (iii) LPs aqueous solvent (VolAqueousOut)

```
pKa1 = 10.2;
pKa2 = 8.4;
logP = 4.72;
logPH = 2.9;
DilutionSample = 1;
VolAqueousIn = 2.4 / DilutionSample;
VolOrganicOut = 0.48 / DilutionSample;
VolOrganicIn = 0.408888 / DilutionSample;
VolAqueousOut = 100 - VolAqueousIn - VolOrganicOut - VolOrganicIn;
pH = {4, 6, 7.4, 9};
```

c) Determination of the molecular abundance for all drug microspecies present in the liposomal system as a function of hypothetical P2H+ values, 0.5 to 4 log10 units (0.1 interval), together with respective theoretical Encapsulation Efficiency (EEt) values

```
FunctionEncap[i_, logP2H_] := Module[{Encapsulated, Name,
   MolesAqueousOut, MolesAqueousIn, MolesOrganicOut, MolesOrganicIn},
  MolesAqueousOut =
   (1 * VolAqueousOut) + (1 * VolAqueousOut * 10^ (pKa1 - pH[[i]])) +
    (1 * VolAqueousOut * 10^ (pKa1 + pKa2 - (2 * pH[[i]])));
  MolesAqueousIn =
   (1 * VolAqueousIn) + (1 * VolAqueousIn * 10^ (pKa1 - pH[[i]])) +
    (1 * VolAqueousIn * 10^ (pKa1 + pKa2 - (2 * pH[[i]])));
  MolesOrganicOut =
   (1 * VolAqueousOut * (10^logP) * (VolOrganicOut / VolAqueousOut)) +
    ((1 * VolAqueousOut * 10^ (pKa1 - pH[[i]])) *
      (10^logPH) * (VolOrganicOut / VolAqueousOut)) +
    ((1 * VolAqueousOut * 10^ (pKa1 + pKa2 - (2 * pH[[i]]))) *
      (10^logP2H) * (VolOrganicOut / VolAqueousOut));
  MolesOrganicIn =
   (1 * VolAqueousIn * (10^logP) * (VolOrganicIn / VolAqueousIn)) +
    ((1 * VolAqueousIn * 10^ (pKa1 - pH[[i]])) *
      (10^logPH) * (VolOrganicIn / VolAqueousIn)) +
    ((1 * VolAqueousIn * 10^ (pKa1 + pKa2 - (2 * pH[[i]]))) *
      (10^logP2H) * (VolOrganicIn / VolAqueousIn));
  Encapsulated =
   (MolesAqueousIn + MolesOrganicOut + MolesOrganicIn) *
    100 / (MolesAqueousOut + MolesAqueousIn + MolesOrganicOut + MolesOrganicIn);
  Encapsulated
 ]
```

```
WorkFunctionEncap =
  Table[FunctionEncap[i, logP2H], {i, 1, Dimensions[pH][[1]]}, {logP2H, 0.5, 4., 0.1}];
Export["Chloroquine_ModelP,PH+,P2H+.dat", Transpose[
   Insert[Transpose[WorkFunctionEncap], {"pH 4", "pH 6", "pH 7.4", "pH 9"}, 1]]];
Transpose[Insert[Transpose[WorkFunctionEncap], {"pH 4", "pH 6", "pH 7.4", "pH 9"}, 1]]
```

```
{{pH 4, 5.1387, 5.81109, 6.64413, 7.67215, 8.9346, 10.4757, 12.3432, 14.5862, 17.2519,
  20.3802, 23.9975, 28.1092, 32.6934, 37.695, 43.0251, 48.5646, 54.1738, 59.7059,
  65.0217, 70.0036, 74.5644, 78.6509, 82.2425, 85.3461, 87.9889, 90.2113, 92.0607,
  93.5863, 94.8356, 95.8526, 96.6765, 97.3414, 97.8763, 98.3055, 98.6492, 98.924},
 {pH 6, 7.64994, 8.28483, 9.07181, 10.0435, 11.2378, 12.6968, 14.4669, 16.5957,
  19.1297, 22.1088, 25.5611, 29.4952, 33.8935, 38.7071, 43.854, 49.222, 54.6773, 60.0768,
  65.2837, 70.1799, 74.6763, 78.7164, 82.2762, 85.359, 87.9891, 90.2043, 92.0502,
  93.5746, 94.824, 95.8419, 96.6671, 97.3333, 97.8694, 98.2998, 98.6445, 98.9201},
 {pH 7.4, 43.9568, 44.1708, 44.438, 44.7707, 45.1839, 45.6953, 46.3259, 47.0991,
  48.0415, 49.1811, 50.5467, 52.1649, 54.0574, 56.2372, 58.7038, 61.44, 64.4087, 67.5536,
  70.8016, 74.0694, 77.2718, 80.3299, 83.1793, 85.7736, 88.0868, 90.1111, 91.8537,
  93.3328, 94.5732, 95.6031, 96.451, 97.1443, 97.708, 98.1641, 98.532, 98.8277},
 {pH 9, 96.6378, 96.6379, 96.6382, 96.6384, 96.6387, 96.6391, 96.6397, 96.6403,
  96.6411, 96.6421, 96.6434, 96.645, 96.6471, 96.6496, 96.6529, 96.6569, 96.662, 96.6683,
  96.6763, 96.6863, 96.6988, 96.7144, 96.7339, 96.758, 96.7879, 96.8247, 96.8699,
  96.925, 96.9917, 97.0717, 97.1665, 97.2774, 97.4053, 97.5502, 97.7112, 97.886}}
```

d) Calculation of EEe vs. EEt variance as a function of P2H + value

```
logP2Hlist = Table[i, {i, 0.5, 4., 0.1}]
DrugEncValues = pHEncValues[[2]]
```

```
{0.5, 0.6, 0.7, 0.8, 0.9, 1., 1.1, 1.2, 1.3, 1.4, 1.5, 1.6, 1.7, 1.8, 1.9, 2., 2.1, 2.2, 2.3,
 2.4, 2.5, 2.6, 2.7, 2.8, 2.9, 3., 3.1, 3.2, 3.3, 3.4, 3.5, 3.6, 3.7, 3.8, 3.9, 4.}
```

```
{9.23, 23.69, 45.06, 76.06}
```

```
FunctionVarianceEnc[i_, j_] := Module[{EncapVar},
  EncapVar = (DrugEncValues[[i]] - WorkFunctionEncap[[i, j]])^2
 ]
```

```
TableFunctionVarianceEnc =
 Transpose[Insert[Transpose[Insert[Table[FunctionVarianceEnc[i, j],
      {i, 1, Dimensions[pH][[1]]}, {j, 1, Dimensions[WorkFunctionEncap][[2]]}],
     logP2Hlist, 1]], Insert[pH, "logP2H+", 1], 1]]
Export["ChloroquineVarianceEncP2H+.dat", TableFunctionVarianceEnc];
```

```
{{logP2H+, 0.5, 0.6, 0.7, 0.8, 0.9, 1., 1.1, 1.2,
  1.3, 1.4, 1.5, 1.6, 1.7, 1.8, 1.9, 2., 2.1, 2.2, 2.3, 2.4, 2.5, 2.6,
  2.7, 2.8, 2.9, 3., 3.1, 3.2, 3.3, 3.4, 3.5, 3.6, 3.7, 3.8, 3.9, 4.},
 {4, 16.7387, 11.689, 6.68671, 2.42688, 0.0872587, 1.55171, 9.69171, 28.689, 64.3514,
  124.327, 218.078, 356.425, 550.53, 810.255, 1142.11, 1547.21, 2019.95, 2547.81,
  3112.71, 3693.43, 4268.58, 4819.26, 5330.82, 5793.66, 6202.96, 6557.98, 6860.93,
  7115.98, 7328.31, 7503.47, 7646.89, 7763.63, 7858.17, 7934.45, 7995.79, 8045.01},
 {6, 257.283, 237.319, 213.692, 186.226, 155.058, 120.85, 85.066, 50.3289, 20.7967,
  2.50006, 3.50115, 33.7, 104.11, 225.513, 406.588, 651.885, 960.21, 1324.,
  1730.04, 2161.32, 2599.6, 3027.9, 3432.34, 3803.06, 4134.37, 4424.16, 4673.12,
  4883.85, 5060.05, 5205.9, 5325.65, 5423.34, 5502.59, 5566.62, 5618.17, 5659.57},
 {7.4, 1.21705, 0.790622, 0.386928, 0.0837215, 0.0153416, 0.403669, 1.6024, 4.15796,
  8.88906, 16.9835, 30.1035, 50.4791, 80.9539, 124.93, 186.154, 268.304, 374.372,
  505.96, 662.628, 841.546, 1037.6, 1243.97, 1453.08, 1657.6, 1851.31, 2029.6, 2189.65,
  2330.26, 2451.56, 2554.6, 2641.03, 2712.77, 2771.81, 2820.05, 2859.25, 2890.96},
 {9, 423.445, 423.452, 423.46, 423.471, 423.484, 423.501, 423.522, 423.549, 423.582,
  423.624, 423.677, 423.744, 423.828, 423.933, 424.066, 424.232, 424.442, 424.704,
  425.033, 425.445, 425.961, 426.605, 427.408, 428.407, 429.645, 431.174, 433.053,
  435.35, 438.137, 441.49, 445.483, 450.179, 455.624, 461.83, 468.773, 476.373}}
```

e) Graphical representation of EEe vs. EEt variance as a function of P2H + value

```
SetDirectory["C:\\Mathematica export"];

TableFunctionVarianceEnc = Import["ChloroquineVarianceEncP2H+.dat", "Table"];

CleanTableVarianceEnc =
  Transpose[Delete[Transpose[Delete[TableFunctionVarianceEnc, 1]], 1]];

NormCleanTableVarianceEnc[i_, j_] := Module[{Sorting, NormValue},
  Sorting = Sort[CleanTableVarianceEnc[[i]]];
  NormValue = 100 - ((CleanTableVarianceEnc[[i, j]] - Sorting[[1]]) *
      100 / (Sorting[[-1]] - Sorting[[1]]))
 ]
```

```
NormTableVarianceEnc =
 Table[NormCleanTableVarianceEnc[i, j], {i, 1, Dimensions[CleanTableVarianceEnc][[1]]},
  {j, 1, Dimensions[CleanTableVarianceEnc][[2]]}]
```

```
{{99.793, 99.8558, 99.918, 99.9709, 100., 99.9818, 99.8806, 99.6445, 99.2012,
  98.4557, 97.2903, 95.5706, 93.1579, 89.9294, 85.8045, 80.7689, 74.8927, 68.3312,
  61.3095, 54.091, 46.9418, 40.0967, 33.7379, 27.9847, 22.897, 18.4841, 14.7183,
  11.548, 8.90867, 6.73139, 4.94861, 3.49761, 2.32239, 1.37426, 0.611722, 0.},
 {95.4962, 95.8491, 96.2668, 96.7523, 97.3032, 97.9079, 98.5405, 99.1545, 99.6766,
  100., 99.9823, 99.4485, 98.2038, 96.0578, 92.8569, 88.5208, 83.0706, 76.6398,
  69.4623, 61.8386, 54.0911, 46.52, 39.3708, 32.8174, 26.961, 21.8383, 17.4374,
  13.7123, 10.5977, 8.01952, 5.90261, 4.17589, 2.77489, 1.64303, 0.731718, 0.},
 {99.9584, 99.9732, 99.9871, 99.9976, 100., 99.9866, 99.9451, 99.8567, 99.6931,
  99.4131, 98.9592, 98.2544, 97.2003, 95.6791, 93.5613, 90.7197, 87.0507, 82.499,
  77.0797, 70.8908, 64.1093, 56.9708, 49.7376, 42.663, 35.9625, 29.7952, 24.259,
  19.3951, 15.1994, 11.635, 8.6453, 6.16382, 4.12176, 2.45298, 1.09692, 0.},
 {100., 99.9874, 99.9714, 99.9514, 99.9262, 99.8945, 99.8545, 99.8042, 99.741,
  99.6614, 99.5612, 99.4352, 99.2768, 99.0776, 98.8272, 98.5125, 98.1174, 97.6216,
  96.9999, 96.2211, 95.2467, 94.0296, 92.5123, 90.6255, 88.2864, 85.3976, 81.8467,
  77.5072, 72.2414, 65.906, 58.3622, 49.4894, 39.2031, 27.4763, 14.3602, 0.}}
```

```
logP2Hlist = Table[i, {i, 0.5, 4, 0.1}]
```

```
{0.5, 0.6, 0.7, 0.8, 0.9, 1., 1.1, 1.2, 1.3, 1.4, 1.5, 1.6, 1.7, 1.8, 1.9, 2., 2.1, 2.2, 2.3,
 2.4, 2.5, 2.6, 2.7, 2.8, 2.9, 3., 3.1, 3.2, 3.3, 3.4, 3.5, 3.6, 3.7, 3.8, 3.9, 4.}
```

```
RowTick = {{1, "pH 4.0"}, {2, "pH 6.0"}, {3, "pH 7.4"}, {4, "pH 9.0"}};
ColumnTick = Table[{i, logP2Hlist[[i]]}, {i, 1, Dimensions[logP2Hlist][[1]]}];
ClusterG = MatrixPlot[NormTableVarianceEnc, Frame → True,
  ColorFunction → Hue, FrameTicks → {RowTick, ColumnTick, RowTick, ColumnTick}]
```

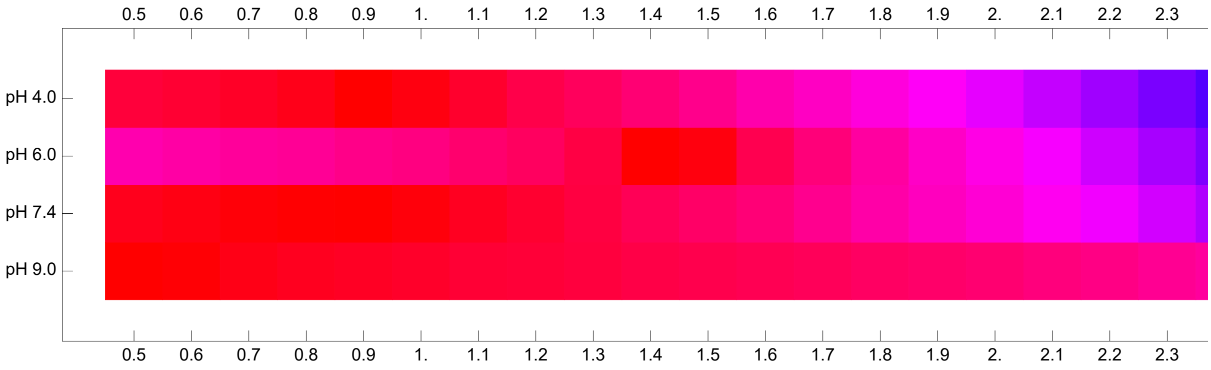

f) Determination of least variant P2H + value across all pH values

```
SetDirectory["C:\\Mathematica export"];
VarpHEncValues = Import["ChloroquineVarianceEncP2H+.dat", "Table"]
```

```
{{logP2H+, 0.5, 0.6, 0.7, 0.8, 0.9, 1., 1.1, 1.2,
  1.3, 1.4, 1.5, 1.6, 1.7, 1.8, 1.9, 2., 2.1, 2.2, 2.3, 2.4, 2.5, 2.6,
  2.7, 2.8, 2.9, 3., 3.1, 3.2, 3.3, 3.4, 3.5, 3.6, 3.7, 3.8, 3.9, 4.},
 {4, 16.7387, 11.689, 6.68671, 2.42688, 0.0872587, 1.55171, 9.69171, 28.689, 64.3514,
  124.327, 218.078, 356.425, 550.53, 810.255, 1142.11, 1547.21, 2019.95, 2547.81,
  3112.71, 3693.43, 4268.58, 4819.26, 5330.82, 5793.66, 6202.96, 6557.98, 6860.93,
  7115.98, 7328.31, 7503.47, 7646.89, 7763.63, 7858.17, 7934.45, 7995.79, 8045.01},
 {6, 257.283, 237.319, 213.692, 186.226, 155.058, 120.85, 85.066, 50.3289, 20.7967,
  2.50006, 3.50115, 33.7, 104.11, 225.513, 406.588, 651.885, 960.21, 1324.,
  1730.04, 2161.32, 2599.6, 3027.9, 3432.34, 3803.06, 4134.37, 4424.16, 4673.12,
  4883.85, 5060.05, 5205.9, 5325.65, 5423.34, 5502.59, 5566.62, 5618.17, 5659.57},
 {7.4, 1.21705, 0.790622, 0.386928, 0.0837215, 0.0153416, 0.403669, 1.6024, 4.15796,
  8.88906, 16.9835, 30.1035, 50.4791, 80.9539, 124.93, 186.154, 268.304, 374.372,
  505.96, 662.628, 841.546, 1037.6, 1243.97, 1453.08, 1657.6, 1851.31, 2029.6, 2189.65,
  2330.26, 2451.56, 2554.6, 2641.03, 2712.77, 2771.81, 2820.05, 2859.25, 2890.96},
 {9, 423.445, 423.452, 423.46, 423.471, 423.484, 423.501, 423.522, 423.549, 423.582,
  423.624, 423.677, 423.744, 423.828, 423.933, 424.066, 424.232, 424.442, 424.704,
  425.033, 425.445, 425.961, 426.605, 427.408, 428.407, 429.645, 431.174, 433.053,
  435.35, 438.137, 441.49, 445.483, 450.179, 455.624, 461.83, 468.773, 476.373}}
```

```
Sumatory = Insert[{Table[(VarpHEncValues[[2, j]] +
       VarpHEncValues[[3, j]] + VarpHEncValues[[4, j]] + VarpHEncValues[[5, j]]),
     {j, 2, Dimensions[VarpHEncValues][[2]]}]}, Delete[VarpHEncValues[[1]], 1], 1]
Export["ChloroquineTotalVarlogP2H+.dat", Sumatory];
```

```
{{0.5, 0.6, 0.7, 0.8, 0.9, 1., 1.1, 1.2, 1.3, 1.4, 1.5, 1.6, 1.7, 1.8, 1.9, 2., 2.1, 2.2,
  2.3, 2.4, 2.5, 2.6, 2.7, 2.8, 2.9, 3., 3.1, 3.2, 3.3, 3.4, 3.5, 3.6, 3.7, 3.8, 3.9, 4.},
 {698.684, 673.251, 644.226, 612.207, 578.645, 546.306, 519.882, 506.725, 517.619,
  567.435, 675.36, 864.349, 1159.42, 1584.63, 2158.92, 2891.63, 3778.97, 4802.48,
  5930.41, 7121.74, 8331.74, 9517.73, 10643.6, 11682.7, 12618.3, 13442.9, 14156.8,
  14765.4, 15278.1, 15705.5, 16059.1, 16349.9, 16588.2, 16782.9, 16942., 17071.9}}
```

```
LogP2H = Sumatory[[1, Ordering[Sumatory[[2]]][[1]]]]
```

```
1.2
```

4. Theoretical estimation of PH+ coefficient for RBC-like LP formulations at pH 7.4. Calculations performed based on theoretical Encapsulation Efficiency (EEt) fitting to experimental data (EEe). EEt data estimated in accordance with the DP,PH+ distribution model (Figure 4). Chloroquine is used here as diprotic drug example.

Printed by Wolfram Mathematica Student Edition

a) Definition of the directory and import of experimental encapsulation efficiency (EEe) data for RBC-like LP formulations

```
SetDirectory["C:\\Mathematica export"];
ChargeEncValuesRaw = Import["charge dependent partitioning.txt", "Table"]
ChargeEncValues = Transpose[Delete[Transpose[Delete[ChargeEncValuesRaw, 1]], 1]];
```

```
{{Drug, PC, PC/PS, PC/PS/PE, PC/PS/PE/PEG}, {Quinine, 66.74, 87.62, 72.4, 70.88},
 {Chloroquine, 40.94, 59.42, 42.78, 50.36}, {Primaquine, 51.2, 86.5, 77.77, 79.62},
 {Tafenoquine, 80.13, 86.65, 89.81, 88.59}, {Quinacrine, 66.91, 92.32, 77.34, 83.58}}
```

b) Definition of parameters for LP suspensions at 10 mM lipid concentration, 100 nm particles. Chloroquine P coefficient is reported in Table 3. All volumetric fractions comprised within our liposomal system include : (i) LPs aqueous core (VolAqueousIn), (ii) LPs lipid bilayer inner (VolOrganicIn) and outer (VolOrganicOut) leaflets, and (iii) LPs aqueous solvent (VolAqueousOut)

```
pKa1 = 10.2;
pKa2 = 8.4;
logP = 4.72;
DilutionSample = 1;
pH = 7.4;
VolAqueousIn = 2.4 / DilutionSample;
VolOrganicOut = 0.48 / DilutionSample;
VolOrganicIn = 0.408888 / DilutionSample;
VolAqueousOut = 100 - VolAqueousIn - VolOrganicOut - VolOrganicIn;
LPformulation = {"PC", "PC/PS", "PC/PS/PE", "PC/PS/PE/PEG"};
```

c) Determination of the molecular abundance for all drug microspecies present in the liposomal system as a function of hypothetical PH+ values, 1 to 6 log10 units (0.1 interval), together with respective theoretical Encapsulation Efficiency (EEt) values

```
FunctionEncap[logPH_] := Module[{Encapsulated, Name,
   MolesAqueousOut, MolesAqueousIn, MolesOrganicOut, MolesOrganicIn},
  MolesAqueousOut =
   (1 * VolAqueousOut) + (1 * VolAqueousOut * 10^ (pKa1 - pH)) +
    (1 * VolAqueousOut * 10^ (pKa1 + pKa2 - (2 * pH)));
  MolesAqueousIn =
   (1 * VolAqueousIn) + (1 * VolAqueousIn * 10^ (pKa1 - pH)) +
    (1 * VolAqueousIn * 10^ (pKa1 + pKa2 - (2 * pH)));
  MolesOrganicOut =
   (1 * VolAqueousOut * (10^logP) * (VolOrganicOut / VolAqueousOut)) +
    ((1 * VolAqueousOut * 10^ (pKa1 - pH)) *
      (10^logPH) * (VolOrganicOut / VolAqueousOut));
  MolesOrganicIn =
   (1 * VolAqueousIn * (10^logP) * (VolOrganicIn / VolAqueousIn)) +
    ((1 * VolAqueousIn * 10^ (pKa1 - pH)) * (10^logPH) * (VolOrganicIn / VolAqueousIn));
  Encapsulated =
   (MolesAqueousIn + MolesOrganicOut + MolesOrganicIn) *
    100 / (MolesAqueousOut + MolesAqueousIn + MolesOrganicOut + MolesOrganicIn);
  Encapsulated
 ]
```

```
WorkFunctionEncap = Table[FunctionEncap[logPH], {logPH, 1., 6., 0.1}];
Export["Chloroquine_ModelPH.dat", WorkFunctionEncap];
WorkFunctionEncap
```

```
{9.31017, 9.48773, 9.71029, 9.98892, 10.3373, 10.772, 11.3133, 11.9855,
 12.8174, 13.8426, 15.0995, 16.6306, 18.4814, 20.6977, 23.3222, 26.3891,
 29.918, 33.9069, 38.3261, 43.1146, 48.1797, 53.403, 58.6502, 63.7842, 68.6799,
 73.2348, 77.3769, 81.0657, 84.2905, 87.0641, 89.4165, 91.3881, 93.0241,
 94.3705, 95.4709, 96.3654, 97.0891, 97.6726, 98.1415, 98.5175, 98.8185, 99.059,
 99.251, 99.404, 99.5259, 99.623, 99.7003, 99.7618, 99.8107, 99.8496, 99.8805}
```

d) Calculation of EEe vs. EEt variances as a function of PH + value and determination of least variant PH + value for all LP formulations

```
logPHlist = Table[i, {i, 1, 6, 0.1}]
DrugEncValues = ChargeEncValues[[2]]
```

```
{1., 1.1, 1.2, 1.3, 1.4, 1.5, 1.6, 1.7, 1.8, 1.9, 2., 2.1, 2.2, 2.3, 2.4, 2.5, 2.6,
 2.7, 2.8, 2.9, 3., 3.1, 3.2, 3.3, 3.4, 3.5, 3.6, 3.7, 3.8, 3.9, 4., 4.1, 4.2, 4.3,
 4.4, 4.5, 4.6, 4.7, 4.8, 4.9, 5., 5.1, 5.2, 5.3, 5.4, 5.5, 5.6, 5.7, 5.8, 5.9, 6.}
```

```
{40.94, 59.42, 42.78, 50.36}
```

```
FunctionVarianceEnc[i_, j_] := Module[{EncapVar},
  EncapVar = (DrugEncValues[[i]] - WorkFunctionEncap[[j]])^2
 ]
```

```
TableFunctionVarianceEnc =
 Transpose[Insert[Transpose[Insert[Table[FunctionVarianceEnc[i, j], {i, 1,
       Dimensions[LPformulation][[1]]}, {j, 1, Dimensions[WorkFunctionEncap][[1]]}],
     logPHlist, 1]], Insert[LPformulation, "logPH+", 1], 1]]
Export["ChloroquineVarianceEnc.dat", TableFunctionVarianceEnc];
```

```
{{logPH+, 1., 1.1, 1.2, 1.3, 1.4, 1.5, 1.6, 1.7, 1.8, 1.9, 2., 2.1, 2.2, 2.3, 2.4, 2.5, 2.6,
  2.7, 2.8, 2.9, 3., 3.1, 3.2, 3.3, 3.4, 3.5, 3.6, 3.7, 3.8, 3.9, 4., 4.1, 4.2, 4.3,
  4.4, 4.5, 4.6, 4.7, 4.8, 4.9, 5., 5.1, 5.2, 5.3, 5.4, 5.5, 5.6, 5.7, 5.8, 5.9, 6.},
 {PC, 1000.45, 989.245, 975.295, 957.97, 936.528, 910.11, 877.742, 838.364,
  790.881, 734.269, 667.732, 590.947, 504.39, 409.751, 310.387, 211.729, 121.485,
  49.4646, 6.83231, 4.72868, 52.4131, 155.327, 313.65, 521.859, 769.501, 1042.96,
  1327.64, 1610.07, 1879.27, 2127.44, 2349.98, 2545.01, 2712.76, 2854.82,
  2973.62, 3071.97, 3152.72, 3218.58, 3272.01, 3315.17, 3349.92, 3377.82,
  3400.17, 3418.04, 3432.31, 3443.7, 3452.78, 3460., 3465.76, 3470.34, 3473.98},
 {PC/PS, 2511., 2493.23, 2471.06, 2443.43, 2409.12, 2366.63, 2314.26, 2250.03,
  2171.8, 2077.3, 1964.31, 1830.93, 1675.97, 1499.42, 1303.05, 1091.04, 870.368,
  650.919, 444.951, 265.868, 126.345, 36.204, 0.592663, 19.0465, 85.7454, 190.85,
  322.449, 468.537, 618.542, 764.198, 899.793, 1021.96, 1129.24, 1221.54,
  1299.67, 1364.96, 1418.96, 1463.26, 1499.36, 1528.62, 1552.24, 1571.25, 1586.5,
  1598.72, 1608.49, 1616.28, 1622.5, 1627.46, 1631.41, 1634.55, 1637.05},
 {PC/PS/PE, 1120.23, 1108.37, 1093.61, 1075.26, 1052.53, 1024.51, 990.154,
  948.302, 897.758, 837.373, 766.211, 683.791, 590.423, 487.628, 378.607, 268.662,
  165.431, 78.732, 19.8369, 0.111926, 29.1567, 112.849, 251.862, 441.177,
  670.804, 927.497, 1196.94, 1465.8, 1723.12, 1961.08, 2174.97, 2362.75, 2524.47,
  2661.58, 2776.33, 2871.39, 2949.48, 3013.19, 3064.9, 3106.67, 3140.31, 3167.33,
  3188.97, 3206.28, 3220.1, 3231.13, 3239.92, 3246.92, 3252.5, 3256.93, 3260.46},
 {PC/PS/PE/PEG, 1685.09, 1670.54, 1652.4, 1629.82, 1601.82, 1567.21, 1524.65,
  1472.6, 1409.45, 1333.52, 1243.3, 1137.67, 1016.25, 879.853, 731.043, 574.604,
  417.876, 270.705, 144.814, 52.4965, 4.75375, 9.26, 68.7267, 180.21, 335.618,
  523.258, 729.911, 942.841, 1151.28, 1347.19, 1525.41, 1683.31, 1820.23, 1936.92,
  2035., 2116.5, 2183.61, 2238.48, 2283.07, 2319.15, 2348.23, 2371.59, 2390.33,
  2405.31, 2417.29, 2426.85, 2434.47, 2440.54, 2445.37, 2449.22, 2452.28}}
```

```
RetrievelogPH[i_] := Module[{LeastPosition, leastlogPHvalue},
  LeastPosition = Ordering[Delete[TableFunctionVarianceEnc[[i]], 1]] + 1;
  leastlogPHvalue = TableFunctionVarianceEnc[[1, LeastPosition[[1]]]];
  leastlogPHvalue
 ]
```

```
TableleastlogPHvalues = Insert[
  {Insert[Table[RetrievelogPH[i], {i, 2, Dimensions[TableFunctionVarianceEnc][[1]]}],
    "logPH+", 1]}, Insert[LPformulation, "LP formulation", 1], 1]
Export["ChloroquineLeastlogPH.dat", TableleastlogPHvalues];
```

```
{{LP formulation, PC, PC/PS, PC/PS/PE, PC/PS/PE/PEG}, {logPH+, 2.9, 3.2, 2.9, 3.}}
```

e) Graphical representation of EEe vs. EEt variance as a function of PH + value

```
CleanTableVarianceEnc =
 Transpose[Delete[Transpose[Delete[TableFunctionVarianceEnc, 1]], 1]]
{{1000.45, 989.245, 975.295, 957.97, 936.528, 910.11, 877.742, 838.364, 790.881,
  734.269, 667.732, 590.947, 504.39, 409.751, 310.387, 211.729, 121.485,
  49.4646, 6.83231, 4.72868, 52.4131, 155.327, 313.65, 521.859, 769.501, 1042.96,
  1327.64, 1610.07, 1879.27, 2127.44, 2349.98, 2545.01, 2712.76, 2854.82,
  2973.62, 3071.97, 3152.72, 3218.58, 3272.01, 3315.17, 3349.92, 3377.82,
  3400.17, 3418.04, 3432.31, 3443.7, 3452.78, 3460., 3465.76, 3470.34, 3473.98},
 {2511., 2493.23, 2471.06, 2443.43, 2409.12, 2366.63, 2314.26, 2250.03, 2171.8,
  2077.3, 1964.31, 1830.93, 1675.97, 1499.42, 1303.05, 1091.04, 870.368,
  650.919, 444.951, 265.868, 126.345, 36.204, 0.592663, 19.0465, 85.7454, 190.85,
  322.449, 468.537, 618.542, 764.198, 899.793, 1021.96, 1129.24, 1221.54,
  1299.67, 1364.96, 1418.96, 1463.26, 1499.36, 1528.62, 1552.24, 1571.25, 1586.5,
  1598.72, 1608.49, 1616.28, 1622.5, 1627.46, 1631.41, 1634.55, 1637.05},
 {1120.23, 1108.37, 1093.61, 1075.26, 1052.53, 1024.51, 990.154, 948.302,
  897.758, 837.373, 766.211, 683.791, 590.423, 487.628, 378.607, 268.662,
  165.431, 78.732, 19.8369, 0.111926, 29.1567, 112.849, 251.862, 441.177,
  670.804, 927.497, 1196.94, 1465.8, 1723.12, 1961.08, 2174.97, 2362.75, 2524.47,
  2661.58, 2776.33, 2871.39, 2949.48, 3013.19, 3064.9, 3106.67, 3140.31, 3167.33,
  3188.97, 3206.28, 3220.1, 3231.13, 3239.92, 3246.92, 3252.5, 3256.93, 3260.46},
 {1685.09, 1670.54, 1652.4, 1629.82, 1601.82, 1567.21, 1524.65, 1472.6, 1409.45,
  1333.52, 1243.3, 1137.67, 1016.25, 879.853, 731.043, 574.604, 417.876,
  270.705, 144.814, 52.4965, 4.75375, 9.26, 68.7267, 180.21, 335.618, 523.258,
  729.911, 942.841, 1151.28, 1347.19, 1525.41, 1683.31, 1820.23, 1936.92,
  2035., 2116.5, 2183.61, 2238.48, 2283.07, 2319.15, 2348.23, 2371.59, 2390.33,
  2405.31, 2417.29, 2426.85, 2434.47, 2440.54, 2445.37, 2449.22, 2452.28}}

NormCleanTableVarianceEnc[i_, j_] := Module[{Sorting, NormValue},
  Sorting = Sort[CleanTableVarianceEnc[[i]]];
  NormValue = 100 - (CleanTableVarianceEnc[[i, j]] * 100 / Sorting[[-1]])
 ]
```

```
NormTableVarianceEnc =
 Table[NormCleanTableVarianceEnc[i, j], {i, 1, Dimensions[CleanTableVarianceEnc][[1]]},
  {j, 1, Dimensions[CleanTableVarianceEnc][[2]]}]
```

```
{{71.2017, 71.5241, 71.9257, 72.4244, 73.0416, 73.8021, 74.7338, 75.8673,
  77.2341, 78.8637, 80.779, 82.9893, 85.4809, 88.2051, 91.0654, 93.9053, 96.503,
  98.5761, 99.8033, 99.8639, 98.4913, 95.5288, 90.9715, 84.9781, 77.8496, 69.978,
  61.7832, 53.6533, 45.9045, 38.7608, 32.3549, 26.7407, 21.9121, 17.8228,
  14.4029, 11.5719, 9.24747, 7.35167, 5.81366, 4.57124, 3.57103, 2.76799,
  2.12467, 1.6102, 1.19933, 0.871564, 0.610325, 0.402253, 0.23662, 0.104828, 0.},
 {0., 0.707446, 1.59059, 2.69069, 4.05731, 5.74927, 7.83509, 10.3928, 13.5083,
  17.2719, 21.7717, 27.0834, 33.2547, 40.2859, 48.1061, 56.5495, 65.3377, 74.0773,
  82.2799, 89.4119, 94.9683, 98.5582, 99.9764, 99.2415, 96.5852, 92.3994,
  87.1585, 81.3406, 75.3667, 69.5659, 64.1659, 59.3006, 55.0283, 51.3525,
  48.2408, 45.6406, 43.4901, 41.726, 40.2884, 39.123, 38.1822, 37.4252, 36.8177,
  36.3312, 35.9422, 35.6317, 35.384, 35.1866, 35.0294, 34.9043, 34.8048},
 {65.642, 66.0056, 66.4586, 67.0214, 67.7183, 68.5776, 69.6315, 70.9151, 72.4653,
  74.3174, 76.4999, 79.0278, 81.8914, 85.0442, 88.3879, 91.76, 94.9261, 97.5852,
  99.3916, 99.9966, 99.1058, 96.5389, 92.2753, 86.4689, 79.4261, 71.5532,
  63.2892, 55.0433, 47.151, 39.8526, 33.2927, 27.5333, 22.5732, 18.3681,
  14.8484, 11.9329, 9.538, 7.5839, 5.99811, 4.71677, 3.68502, 2.85654, 2.19276,
  1.66187, 1.23786, 0.899586, 0.629961, 0.415202, 0.24424, 0.108205, 0.},
 {31.2847, 31.8779, 32.6177, 33.5383, 34.6803, 36.0915, 37.8273, 39.9495,
  42.5249, 45.6211, 49.3, 53.6075, 58.559, 64.121, 70.1892, 76.5685, 82.9597,
  88.9611, 94.0947, 97.8593, 99.8061, 99.6224, 97.1974, 92.6513, 86.314, 78.6624,
  70.2354, 61.5524, 53.0526, 45.0636, 37.796, 31.3573, 25.7739, 21.0153, 17.016,
  13.6926, 10.9558, 8.71836, 6.89986, 5.4287, 4.24297, 3.29014, 2.52626, 1.91502,
  1.42665, 1.03693, 0.726212, 0.478679, 0.281599, 0.124763, -1.42109 × 10^-14}}
```

```
RowTick = {{1, "PC"}, {2, "PC/PS"}, {3, "PC/PS/PE"}, {4, "PC/PS/PE/PEG"}};
ColumnTick = Table[{i, logPHlist[[i]]}, {i, 1, Dimensions[logPHlist][[1]]}];
ClusterG = MatrixPlot[NormTableVarianceEnc, Frame → True,
  ColorFunction → Hue, FrameTicks → {RowTick, ColumnTick, RowTick, ColumnTick}]
```

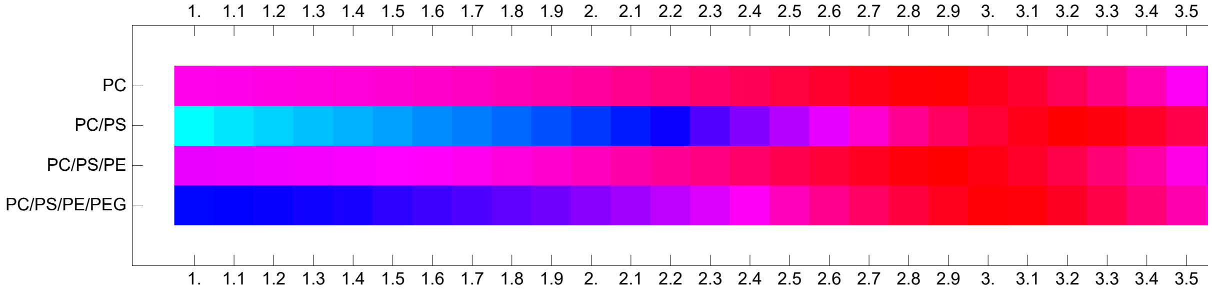

5. Modelling the Encapsulation Efficiency of drugs in a PC-LPs suspension and in response to transmembrane pH gradients (LPs aqueous core pH range 4.0-7.4; LPs suspended in solution at pH 7.4). Data estimated in accordance with all three distribution models reported in this work: DP (Figure 1), DP,PH+ (Figure 4), and DP,PH+,P2H+ (Figure 6). Chloroquine is used as diprotic drug

example.

a) Definition of parameters considering a LPs suspension at 10 mM lipid concentration, 100 nm particles. Chloroquine P, PH+, P2H+ coefficients are reported in Table 3. All volumetric fractions comprised within our liposomal system include: (i) LPs aqueous core (VolAqueousIn), (ii) LPs lipid bilayer inner (VolOrganicIn) and outer (VolOrganicOut) leaflets, and (iii) LPs aqueous solvent (VolAqueousOut)

```
SetDirectory["C:\\Mathematica export"];
pKa1 = 10.2;
pKa2 = 8.4;
logP = 4.72;
logPH = 2.9;
logP2H = 1.2;
DilutionSample = 1;
VolAqueousIn = 2.4 / DilutionSample;
VolOrganicOut = 0.48 / DilutionSample;
VolOrganicIn = 0.408888 / DilutionSample;
VolAqueousOut = 100 - VolAqueousIn - VolOrganicOut - VolOrganicIn;
pHOut = 7.4;
```

b) Determination of (i) the molecular abundance for all drug microspecies present in the liposomal system, and (ii) respective theoretical Encapsulation Efficiency (EEt) values. Data estimated in accordance with DP distribution model

```
FunctionEncap[iin_] := Module[{Encapsulated, Name, MolesAqueousOut,
   MolesAqueousIn, MolesOrganicPOut, MolesOrganicPIn, TotalMolesEncap},
  MolesAqueousOut =
   (1 * VolAqueousOut) + (1 * VolAqueousOut * 10^(pKa1 - pHOut)) +
    (1 * VolAqueousOut * 10^(pKa1 + pKa2 - (2 * pHOut)));
  MolesAqueousIn =
   (1 * VolAqueousIn) + (1 * VolAqueousIn * 10^(pKa1 - iin)) +
    (1 * VolAqueousIn * 10^(pKa1 + pKa2 - (2 * iin)));
  MolesOrganicPOut =
   1 * VolAqueousOut * (10^logP) * (VolOrganicOut / VolAqueousOut);
  MolesOrganicPIn =
   1 * VolAqueousIn * (10^logP) * (VolOrganicIn / VolAqueousIn);
  Encapsulated =
   (MolesAqueousIn + MolesOrganicPOut + MolesOrganicPIn) *
    100 / (MolesAqueousOut + MolesAqueousIn + MolesOrganicPOut + MolesOrganicPIn);
  TotalMolesEncap = MolesAqueousIn + MolesOrganicPOut + MolesOrganicPIn;
  {iin, Encapsulated, ((MolesOrganicPOut * 100 / TotalMolesEncap) +
      (MolesOrganicPIn * 100 / TotalMolesEncap)),
   (MolesAqueousIn * 100 / TotalMolesEncap)}
 ]
```

```
WorkFunctionEncap = Table[FunctionEncap[i], {i, 4., 7.5, 0.1}];
Export["Chloroquine_pHdepEE_P.dat", WorkFunctionEncap];
WorkFunctionEncap
```

```
{{4., 99.9993, 0.0000488223, 100.}, {4.1, 99.9989, 0.0000773773, 99.9999},
 {4.2, 99.9982, 0.000122633, 99.9999}, {4.3, 99.9972, 0.000194357, 99.9998},
 {4.4, 99.9956, 0.000308029, 99.9997}, {4.5, 99.993, 0.000488179, 99.9995},
 {4.6, 99.9889, 0.000773684, 99.9992}, {4.7, 99.9824, 0.00122615, 99.9988},
 {4.8, 99.972, 0.0019432, 99.9981}, {4.9, 99.9557, 0.00307953, 99.9969},
 {5., 99.9298, 0.00488025, 99.9951}, {5.1, 99.8888, 0.00773365, 99.9923},
 {5.2, 99.824, 0.0122549, 99.9877}, {5.3, 99.7213, 0.0194181, 99.9806},
 {5.4, 99.5592, 0.0307658, 99.9692}, {5.5, 99.3035, 0.0487391, 99.9513},
 {5.6, 98.9012, 0.0771992, 99.9228}, {5.7, 98.2712, 0.122247, 99.8778},
 {5.8, 97.2907, 0.193511, 99.8065}, {5.9, 95.7802, 0.306149, 99.6939},
 {6., 93.489, 0.483953, 99.516}, {6.1, 90.0937, 0.76407, 99.2359},
 {6.2, 85.2317, 1.20405, 98.796}, {6.3, 78.6001, 1.89193, 98.1081},
 {6.4, 70.1291, 2.95982, 97.0402}, {6.5, 60.1698, 4.59992, 95.4001},
 {6.6, 49.5391, 7.0782, 92.9218}, {6.7, 39.2983, 10.7335, 89.2665},
 {6.8, 30.3609, 15.9387, 84.0613}, {6.9, 23.2066, 22.9947, 77.0053},
 {7., 17.8633, 31.9514, 68.0486}, {7.1, 14.0741, 42.4246, 57.5754},
 {7.2, 11.4831, 53.5651, 46.4349}, {7.3, 9.75401, 64.2925, 35.7075},
 {7.4, 8.6178, 73.6852, 26.3148}, {7.5, 7.87797, 81.2577, 18.7423}}
```

c) Determination of (i) the molecular abundance for all drug microspecies present in the liposomal system, and (ii) respective theoretical Encapsulation Efficiency (EEt) values. Data estimated in accordance with DP,PH+ distribution model

```
FunctionEncap[i_] :=
 Module[{Encapsulated, Name, MolesAqueousOut, MolesAqueousIn, MolesOrganicPOut,
   MolesOrganicPHOut, MolesOrganicPIn, MolesOrganicPHIn, TotalMolesEncap},
  MolesAqueousOut =
   (1 * VolAqueousOut) + (1 * VolAqueousOut * 10^(pKa1 - pHOut)) +
    (1 * VolAqueousOut * 10^(pKa1 + pKa2 - (2 * pHOut)));
  MolesAqueousIn =
   (1 * VolAqueousIn) + (1 * VolAqueousIn * 10^(pKa1 - i)) +
    (1 * VolAqueousIn * 10^(pKa1 + pKa2 - (2 * i)));
  MolesOrganicPOut =
   1 * VolAqueousOut * (10^logP) * (VolOrganicOut / VolAqueousOut);
  MolesOrganicPHOut =
   (1 * VolAqueousOut * 10^(pKa1 - pHOut)) * (10^logPH) * (VolOrganicOut / VolAqueousOut);
  MolesOrganicPIn =
   1 * VolAqueousIn * (10^logP) * (VolOrganicIn / VolAqueousIn);
  MolesOrganicPHIn =
   (1 * VolAqueousIn * 10^(pKa1 - i)) * (10^logPH) * (VolOrganicIn / VolAqueousIn);
  Encapsulated =
   (MolesAqueousIn + MolesOrganicPOut + MolesOrganicPHOut + MolesOrganicPIn +
      MolesOrganicPHIn) * 100 / (MolesAqueousOut + MolesAqueousIn +
       MolesOrganicPOut + MolesOrganicPHOut + MolesOrganicPIn + MolesOrganicPHIn);
  TotalMolesEncap = MolesAqueousIn + MolesOrganicPOut + MolesOrganicPHOut +
    MolesOrganicPIn + MolesOrganicPHIn;
  {i, Encapsulated, ((MolesOrganicPOut * 100 / TotalMolesEncap) +
     (MolesOrganicPIn * 100 / TotalMolesEncap)),
   (MolesOrganicPHOut * 100 / TotalMolesEncap),
   (MolesOrganicPHIn * 100 / TotalMolesEncap), (MolesAqueousIn * 100 / TotalMolesEncap)}
 ]
```

```
WorkFunctionEncap = Table[FunctionEncap[i], {i, 4., 7.5, 0.1}];
Export["Chloroquine_pHdepEE_P,PH.dat", WorkFunctionEncap];
WorkFunctionEncap
```

```
{{4., 99.9993, 0.0000485606, 0.000250425, 0.535847, 99.4639},
 {4.1, 99.9989, 0.0000768558, 0.000396342, 0.673649, 99.3259},
 {4.2, 99.9983, 0.000121594, 0.000627057, 0.846584, 99.1527},
 {4.3, 99.9972, 0.000192288, 0.000991624, 1.06343, 98.9354},
 {4.4, 99.9956, 0.000303911, 0.00156726, 1.33507, 98.6631},
 {4.5, 99.9931, 0.00047999, 0.00247529, 1.6749, 98.3221},
 {4.6, 99.9891, 0.000757411, 0.00390594, 2.09937, 97.896},
 {4.7, 99.9828, 0.00119385, 0.00615662, 2.62849, 97.3642},
 {4.8, 99.973, 0.00187915, 0.00969073, 3.28639, 96.702},
 {4.9, 99.9575, 0.00295275, 0.0152272, 4.10189, 95.8799},
 {5., 99.9334, 0.00462976, 0.0238755, 5.10876, 94.8627},
 {5.1, 99.8959, 0.00723999, 0.0373364, 6.34593, 93.6095},
 {5.2, 99.8379, 0.0112849, 0.0581957, 7.85695, 92.0736},
 {5.3, 99.7485, 0.0175192, 0.0903457, 9.68882, 90.2033},
 {5.4, 99.612, 0.0270649, 0.139573, 11.8895, 87.9438},
 {5.5, 99.4054, 0.0415655, 0.214352, 14.5041, 85.24},
 {5.6, 99.0961, 0.063384, 0.326869, 17.5686, 82.0411},
 {5.7, 98.6395, 0.0958459, 0.494274, 21.1024, 78.3075},
 {5.8, 97.9766, 0.143511, 0.740081, 25.0982, 74.0182},
 {5.9, 97.0335, 0.212444, 1.09556, 29.5122, 69.1798},
 {6., 95.7237, 0.310429, 1.60087, 34.2547, 63.834},
 {6.1, 93.9554, 0.447055, 2.30545, 39.1849, 58.0626},
 {6.2, 91.6442, 0.633577, 3.26733, 44.112, 51.9871},
 {6.3, 88.7315, 0.882478, 4.55091, 48.8047, 45.762},
 {6.4, 85.2043, 1.20667, 6.22278, 53.0087, 39.5618},
 {6.5, 81.1099, 1.61836, 8.34582, 56.4719, 33.564},
 {6.6, 76.5597, 2.12754, 10.9717, 58.9706, 27.9302},
 {6.7, 71.717, 2.74044, 14.1323, 60.3361, 22.7912},
 {6.8, 66.773, 3.45785, 17.832, 60.4733, 18.2368},
 {6.9, 61.9174, 4.27395, 22.0406, 59.3727, 14.3127},
 {7., 57.3127, 5.17563, 26.6905, 57.1111, 11.0228},
 {7.1, 53.0786, 6.14281, 31.6783, 53.8424, 8.33654},
 {7.2, 49.2872, 7.14989, 36.8717, 49.7802, 6.19815},
 {7.3, 45.9675, 8.16809, 42.1225, 45.1729, 4.5365},
 {7.4, 43.1146, 9.16838, 47.281, 40.2763, 3.27425},
 {7.5, 40.7002, 10.1245, 52.2115, 35.3288, 2.33523}}
```

d) Determination of (i) the molecular abundance for all drug microspecies present in the liposomal system, and (ii) respective theoretical Encapsulation Efficiency (EEt) values. Data estimated in accordance with DP,PH+,P2H+ distribution model

```
FunctionEncap[i_] := Module[{Encapsulated, Name, MolesAqueousOut,
    MolesAqueousIn, MolesOrganicPOut, MolesOrganicPHOut, MolesOrganicP2HOut,
    MolesOrganicPIn, MolesOrganicPHIn, MolesOrganicP2HIn, TotalMolesEncap},
  MolesAqueousOut =
   (1 * VolAqueousOut) + (1 * VolAqueousOut * 10^(pKa1 - pHOut)) +
    (1 * VolAqueousOut * 10^(pKa1 + pKa2 - (2 * pHOut)));
  MolesAqueousIn =
   (1 * VolAqueousIn) + (1 * VolAqueousIn * 10^(pKa1 - i)) +
    (1 * VolAqueousIn * 10^(pKa1 + pKa2 - (2 * i)));
  MolesOrganicPOut =
   1 * VolAqueousOut * (10^logP) * (VolOrganicOut / VolAqueousOut);
  MolesOrganicPHOut =
   (1 * VolAqueousOut * 10^(pKa1 - pHOut)) * (10^logPH) * (VolOrganicOut / VolAqueousOut);
  MolesOrganicP2HOut = (1 * VolAqueousOut * 10^(pKa1 + pKa2 - (2 * pHOut))) *
    (10^logP2H) * (VolOrganicOut / VolAqueousOut);
  MolesOrganicPIn =
   1 * VolAqueousIn * (10^logP) * (VolOrganicIn / VolAqueousIn);
  MolesOrganicPHIn =
   (1 * VolAqueousIn * 10^(pKa1 - i)) * (10^logPH) * (VolOrganicIn / VolAqueousIn);
  MolesOrganicP2HIn = (1 * VolAqueousIn * 10^(pKa1 + pKa2 - (2 * i))) *
    (10^logP2H) * (VolOrganicIn / VolAqueousIn);
  Encapsulated =
   (MolesAqueousIn + MolesOrganicPOut + MolesOrganicPHOut +
      MolesOrganicP2HOut + MolesOrganicPIn + MolesOrganicPHIn + MolesOrganicP2HIn) *
    100 / (MolesAqueousOut + MolesAqueousIn + MolesOrganicPOut + MolesOrganicPHOut +
       MolesOrganicP2HOut + MolesOrganicPIn + MolesOrganicPHIn + MolesOrganicP2HIn);
  TotalMolesEncap = MolesAqueousIn + MolesOrganicPOut + MolesOrganicPHOut +
    MolesOrganicP2HOut + MolesOrganicPIn + MolesOrganicPHIn + MolesOrganicP2HIn;
  {i, Encapsulated, ((MolesOrganicPOut * 100 / TotalMolesEncap) +
      (MolesOrganicPIn * 100 / TotalMolesEncap)),
   (MolesOrganicPHOut * 100 / TotalMolesEncap),
   (MolesOrganicP2HOut * 100 / TotalMolesEncap),
   (MolesOrganicPHIn * 100 / TotalMolesEncap),
   (MolesOrganicP2HIn * 100 / TotalMolesEncap), (MolesAqueousIn * 100 / TotalMolesEncap)}
 ]
```

```
WorkFunctionEncap = Table[FunctionEncap[i], {i, 4., 7.5, 0.1}];
Export["Chloroquine_pHdepEE_P2H.dat", WorkFunctionEncap];
WorkFunctionEncap
```

```
{{4., 99.9998, 0.0000131758, 0.0000679469, 0.0000135572, 0.145389, 72.8674, 26.9872},
 {4.1, 99.9997, 0.0000208742, 0.000107648, 0.0000214786, 0.182965, 72.8397, 26.9772},
 {4.2, 99.9995, 0.0000330676, 0.000170529, 0.0000340249, 0.230229, 72.8049, 26.9646},
 {4.3, 99.9992, 0.0000523771, 0.000270107, 0.0000538935, 0.289667, 72.7611, 26.9489},
 {4.4, 99.9988, 0.0000829493, 0.000427767, 0.0000853507, 0.364393, 72.706, 26.929},
 {4.5, 99.9981, 0.000131341, 0.000677319, 0.000135143, 0.458307, 72.6367, 26.904},
 {4.6, 99.997, 0.000207911, 0.00107219, 0.00021393, 0.576282, 72.5496, 26.8727},
 {4.7, 99.9953, 0.000329019, 0.00169674, 0.000338544, 0.7244, 72.44, 26.8332},
 {4.8, 99.9925, 0.00052047, 0.00268404, 0.000535537, 0.910234, 72.3024, 26.7836},
 {4.9, 99.9882, 0.000822917, 0.00424376, 0.000846741, 1.14318, 72.1296, 26.7213},
 {5., 99.9813, 0.00130031, 0.00670567, 0.00133796, 1.43485, 71.9127, 26.6431},
 {5.1, 99.9705, 0.00205306, 0.0105876, 0.0021125, 1.79953, 71.6406, 26.5451},
 {5.2, 99.9534, 0.0032384, 0.0167003, 0.00333216, 2.2547, 71.2998, 26.4222},
 {5.3, 99.9266, 0.00510183, 0.0263099, 0.00524952, 2.82152, 70.8734, 26.2685},
 {5.4, 99.8846, 0.00802507, 0.041385, 0.00825739, 3.52538, 70.3406, 26.0764},
 {5.5, 99.819, 0.0125988, 0.0649714, 0.0129635, 4.39628, 69.6764, 25.8368},
 {5.6, 99.7169, 0.019731, 0.101752, 0.0203022, 5.46898, 68.8504, 25.5388},
 {5.7, 99.5586, 0.0308065, 0.158868, 0.0316984, 6.78266, 67.8266, 25.1694},
 {5.8, 99.3152, 0.0479153, 0.247098, 0.0493024, 8.37976, 66.5628, 24.7131},
 {5.9, 98.9439, 0.0741701, 0.382492, 0.0763173, 10.3035, 65.0109, 24.1526},
 {6., 98.3841, 0.114129, 0.588557, 0.117433, 12.5937, 63.1178, 23.4684},
 {6.1, 97.5528, 0.174319, 0.898958, 0.179366, 15.2793, 60.8279, 22.6402},
 {6.2, 96.3422, 0.26383, 1.36056, 0.271467, 18.3688, 58.0873, 21.6481},
 {6.3, 94.6234, 0.394845, 2.0362, 0.406276, 21.8366, 54.8509, 20.4752},
 {6.4, 92.2607, 0.582912, 3.00606, 0.599787, 25.6071, 51.0929, 19.1113},
 {6.5, 89.1402, 0.846568, 4.36572, 0.871076, 29.5406, 46.8187, 17.5574},
 {6.6, 85.2124, 1.20589, 6.21875, 1.2408, 33.4246, 42.0791, 15.8308},
 {6.7, 80.5338, 1.67964, 8.66186, 1.72827, 36.9806, 36.9806, 13.969},
 {6.8, 75.2868, 2.281, 11.763, 2.34704, 39.8917, 31.6871, 12.0301},
 {6.9, 69.7561, 3.01281, 15.537, 3.10003, 41.8532, 26.4076, 10.0894},
 {7., 64.2656, 3.86388, 19.9259, 3.97574, 42.6365, 21.3689, 8.22911},
 {7.1, 59.1036, 4.80825, 24.796, 4.94745, 42.1448, 16.7782, 6.52538},
 {7.2, 54.4706, 5.80826, 29.953, 5.9764, 40.4393, 12.788, 5.0351},
 {7.3, 50.4649, 6.82087, 35.175, 7.01833, 37.7222, 9.47539, 3.78826},
 {7.4, 47.0991, 7.80487, 40.2494, 8.03082, 34.2865, 6.84105, 2.78731},
 {7.5, 44.3293, 8.72672, 45.0034, 8.97936, 30.4515, 4.82623, 2.01284}}
```

6. Modelling the Encapsulation Efficiency of drugs in a RBCs suspension at pH 7.4. Data estimated using Chloroquine as diprotic drug example and in accordance with the DP,PH+ distribution model (Figure 4).

a) Definition of parameters simulating a RBCs suspension with asymmetric lipid bilayer, *i.e.* PC and PC/PS lipid composition as outer and inner lipid bilayer leaflets. RBCs concentration replicates human haematocrit. All

Printed by Wolfram Mathematica Student Edition

volumetric fractions comprised within the RBC-like system include: (i) RBCs aqueous core (VolAqueousIn), (ii) RBCs lipid bilayer inner (VolOrganicIn) and outer (VolOrganicOut) leaflets, and (iii) RBCs aqueous solvent (VolAqueousOut). Chloroquine P, PH+ coefficients are reported in Table 3.

```
SetDirectory["C:\\Mathematica export"];
pKa1 = 10.2;
pKa2 = 8.4;
logP = 4.72;
logPHPC = 2.9;
logPHPCPS = 3.2;
DilutionSample = 1;
VolAqueousIn = 39.80197759 / DilutionSample;
VolOrganicOut = 0.099011203 / DilutionSample;
VolOrganicIn = 0.099011203 / DilutionSample;
VolAqueousOut = 100 - VolAqueousIn - VolOrganicOut - VolOrganicIn;
pHOut = 7.4;
pHIn = 7.4;
```

b) Determination of (i) the molecular abundance for all drug microspecies present in the liposomal system, and (ii) respective theoretical Encapsulation Efficiency (EEt) values

```
MolesAqueousOut =
  (1 * VolAqueousOut) + (1 * VolAqueousOut * 10^ (pKa1 - pHOut)) +
   (1 * VolAqueousOut * 10^ (pKa1 + pKa2 - (2 * pHOut)));
MolesAqueousIn =
  (1 * VolAqueousIn) + (1 * VolAqueousIn * 10^ (pKa1 - pHIn)) +
   (1 * VolAqueousIn * 10^ (pKa1 + pKa2 - (2 * pHIn)));
MolesOrganicPOut =
  1 * VolAqueousOut * (10^logP) * (VolOrganicOut / VolAqueousOut);
MolesOrganicPHOut = (1 * VolAqueousOut * 10^ (pKa1 - pHOut)) *
   (10^logPHPC) * (VolOrganicOut / VolAqueousOut);
MolesOrganicPIn =
  1 * VolAqueousIn * (10^logP) * (VolOrganicIn / VolAqueousIn);
MolesOrganicPHIn =
  (1 * VolAqueousIn * 10^ (pKa1 - pHIn)) * (10^logPHPCPS) * (VolOrganicIn / VolAqueousIn);
Encapsulated =
  (MolesAqueousIn + MolesOrganicPOut + MolesOrganicPHOut + MolesOrganicPIn +
     MolesOrganicPHIn) * 100 / (MolesAqueousOut + MolesAqueousIn +
      MolesOrganicPOut + MolesOrganicPHOut + MolesOrganicPIn + MolesOrganicPHIn);
TotalMolesEncap = MolesAqueousIn + MolesOrganicPOut + MolesOrganicPHOut +
   MolesOrganicPIn + MolesOrganicPHIn;
ListPS = {Encapsulated, ((MolesOrganicPOut * 100 / TotalMolesEncap) +
     (MolesOrganicPIn * 100 / TotalMolesEncap)),
  (MolesOrganicPHOut * 100 / TotalMolesEncap), (MolesOrganicPHIn * 100 / TotalMolesEncap),
  (MolesAqueousIn * 100 / TotalMolesEncap)}

{51.1048, 2.38733, 11.3994, 22.7448, 63.4685}
```
